# Theme discovery from gene lists for identification and viewing of multiple functional groups

**DOI:** 10.1186/1471-2105-6-162

**Published:** 2005-06-29

**Authors:** Petri Pehkonen, Garry Wong, Petri Törönen

**Affiliations:** 1 Department of Neurobiology, A.I. Virtanen-Institute, University of Kuopio P.O. Box 1627, FIN-70211 Kuopio, Finland; 2 Department of Computer Science, University of Kuopio P.O. Box 1627, FIN-70211 Kuopio, Finland; 3 Bioinformatics Group, Institute of Biotechnology, P.O. Box 56, 00014 University of Helsinki, Finland

## Abstract

**Background:**

High throughput methods of the genome era produce vast amounts of data in the form of gene lists. These lists are large and difficult to interpret without advanced computational or bioinformatic tools. Most existing methods analyse a gene list as a single entity although it is comprised of multiple gene groups associated with separate biological functions. Therefore it is imperative to define and visualize gene groups with unique functionality within gene lists.

**Results:**

In order to analyse the functional heterogeneity within a gene list, we have developed a method that clusters genes to groups with homogenous functionalities. The method uses Non-negative Matrix Factorization (NMF) to create several clustering results with varying numbers of clusters. The obtained clustering results are combined into a simple graphical presentation showing the functional groups over-represented in the analyzed gene list. We demonstrate its performance on two data sets and show results that improve upon existing methods. The comparison also shows that our method creates a more simplified view that aids in discovery of biological themes within the list and discards less informative classes from the results.

**Conclusion:**

The presented method and associated software are useful for the identification and interpretation of biological functions associated with gene lists and are especially useful for the analysis of large lists.

## Background

Recent developments in biosciences have created a dramatic change from the analysis of a few genes to large gene lists. These lists are usually selected at the genomic level by criteria such as activity in a stress treatment [1], importance to cell survival in a specific growth condition [2], or as a result of clustering genes by expression profiles [3]. As current high throughput methods produce a vast amount of data as gene lists, the subsequent analysis tends to be a bottleneck due the size of the data set and the high probability of false positive genes among the lists.

One solution to analyse a gene list is to draw information either from the existing literature or from the databases representing whole genome [4,5] or proteome annotations [6,7], and then using these to guide the analysis. Most of these databases simplify the analysis by classifying genes to the biological categories or classes that present their function, localization, or partnership in some protein complex. A further step is to estimate the statistical significance of associations between the classes and genes of the obtained list. Several applications have been recently reported for such analysis [8,9]. Most of these applications compare the frequency of gene classes in the user supplied gene list, obtained by various criteria, to the remaining genes that did not fulfill the criteria. The latter often includes the rest of the genes from the whole genome. The usual outcome from these methods is a sorted list of biological classes considered important. These methods have been beneficial to data analysis by guiding the process towards the most important features in the gene list [10-13]. In addition, the observation of multiple genes from the same functional class increases confidence in results obtained from high throughput methods.

While these methods are useful, several weaknesses are associated with this approach. A gene list can have a heterogeneous structure with multiple dissimilar gene groups such as stress response, a specific metabolic pathway, and protein degradation. The basic statistics used by the previously mentioned methods are often insufficient to reveal this kind of heterogeneity from the associated functional classes. Rather, they have a tendency to be biased toward the gene sub-group associated with the most over-represented functional classes within the analyzed list of genes. This overwhelms many important, but less over-represented, classes that are associated with the rest of the genes in the list. Therefore, it could be hypothesized that there exists other interesting biological functions among the genes that are not members of the best scoring classes. As such, the existing methods do not address this question and thus there is a need for an approach that would concentrate on the possible heterogeneity in the gene list. In the current work, we propose the clustering of a gene list for finding gene groups that differ in functional class annotations.

## Results

### Principle of the method

**Figure 1:**
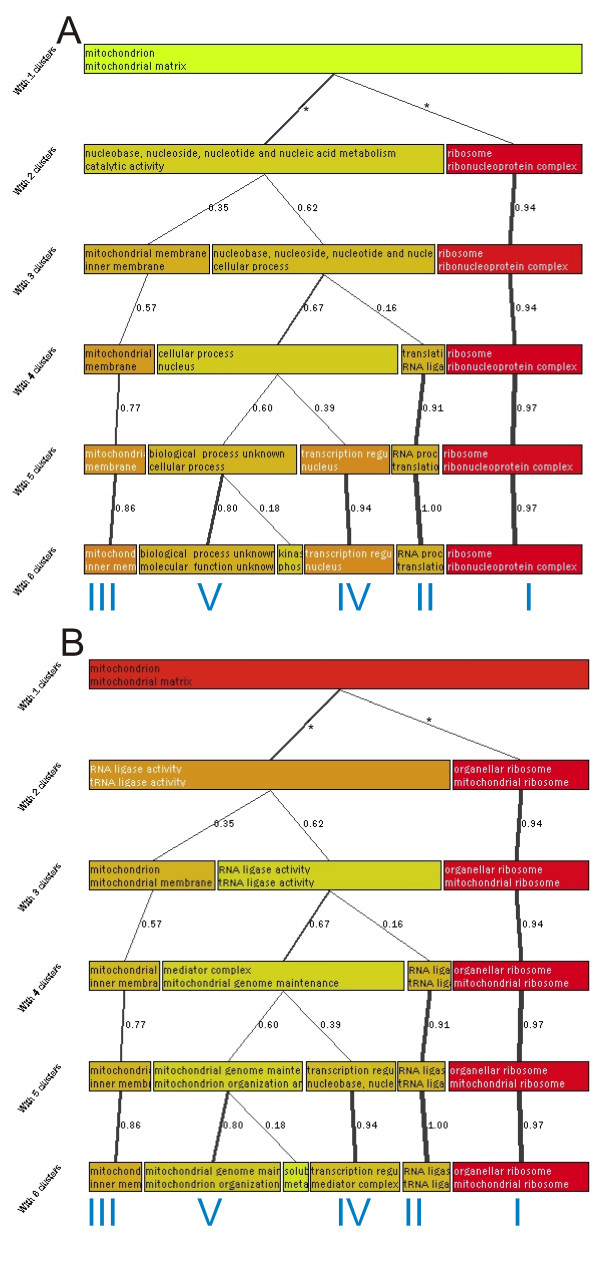
**Graphical results from the analysis of H_2_O_2 _dataset**. The figure shows the non-nested hierarchical clustering tree obtained from GENERATOR with the H_2_O_2 _dataset. Each layer presents one clustering solution and each box a single cluster. Boxes show the two best scoring functional classes and the colour of the box corresponds to the over-representation of the best scoring functional class. Best correlating clusters between the consecutive clustering layers are connected with lines. A thicker line indicates a stronger correlation. The correlation value is indicated beside each line. The lines between the first and second level (marked with asterisks) do not present any value as the correlation measure is not defined here. Section A presents a view where two functional classes that contributed most to the cluster formation are shown for each cluster. Section B shows more informative visualization, the default view of GENERATOR, where two classes that were most over-represented in both the original sample list and in the cluster in question are shown. Note the conserved clusters across the different clustering results. We have marked them with Roman numerals.

**Figure 2:**
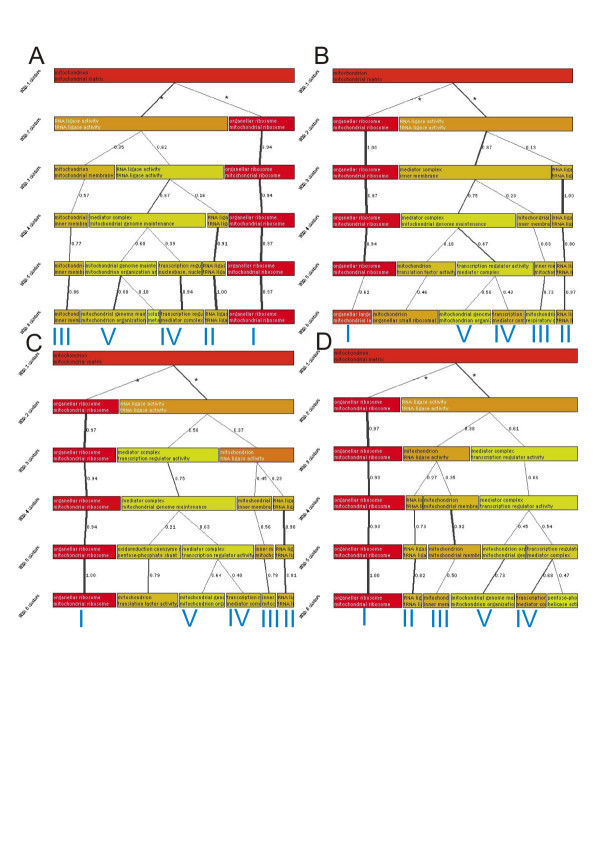
**Replications of non-nested hierarchical clustering tree with H_2_O_2 _dataset**. The figure presents the four replications for the non-nested hierarchical clustering graph for H_2_O_2 _dataset. We have marked the conserved gene clusters with the same Roman numerals as in figure 1. Notice that most clusters (especially I, II and III) can be observed over several levels in each cluster tree.

**Figure 3:**
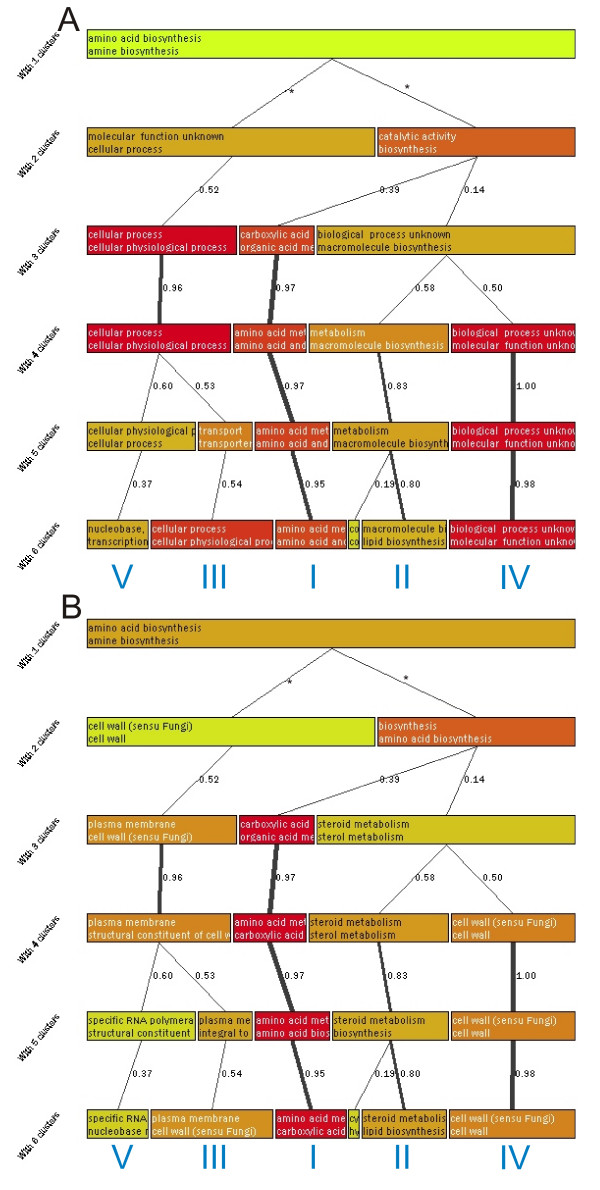
**Graphical results from the analysis of itraconanzole dataset**. The figure shows the non-nested hierarchical clustering tree obtained from GENERATOR with the itraconanzole dataset. Section A shows the tree with functional classes that contributed most to the formation of each cluster. Section B shows the default view of GENERATOR with the highest over-represented functional classes in the original list and in the cluster in question. The details of the presentation are explained in text for figure 1. Also in this figure we highlight some conserved clusters with roman numbers.

Our method takes, as input, the user given gene list chosen by some selection criteria. The selected list is referred to as a sample gene list, and the gene list that did not meet the criteria is referred to as a reference gene list. The aim is then clustering the sample gene list for finding gene groups with different functional class annotations. The clustering is solely based on the gene associations with functional classes obtained from Gene Ontology (GO) database [14], and the measurements like gene expression level or sequence similarity are not used. As a clustering method, we use Non-negative Matrix Factorization (NMF) [15] to create a k-means like partition. The well known weakness with this type of clustering approach is the requirement to select the number of clusters and the initialization for the algorithm. We circumvent this weakness by using a non-nested hierarchical clustering scheme, which allows parallel visualization of several different clustering results. Here, a gene list is repeatedly divided into a growing number of clusters by clustering from random starting initializations. The different clustering results are presented in consecutive levels ordered with the number of clusters, with the first level presenting the gene list without any clustering. Strongly correlating clusters between the consecutive levels are connected by edges forming a non-nested hierarchy (see figures 1, 2, 3). The output graph highlights the clusters that stay similar through the different clustering levels despite the varying number of divisions and different random starting initializations. The resulting visualization can be used either for obtaining suitable grouping for a gene list, or identifying individual clusters that are of interest.

In the non-nested clustering hierarchy, the cluster contents are described with the most representative functional classes. For this, a combination of three different measures was used to show over-represented classes within each cluster. The measures are positive/negative signed ten based logarithmic transforms [10] of p-values calculated with Fisher's test [16,17], which compares class frequencies between two sets of genes. The first measure, "Original log(p)" (denoted by O.log(p)), makes a comparison between the whole user given sample and reference gene lists. It reports class over-representation that was observed before any clustering. Because of the wide usage of this measure reported in the literature [10,11,18], it is suitable for method comparison. As a comparison, the second measure, "Sample log(p)" (denoted by S.log(p)), concentrates fully on clustered sample gene list by comparing a single cluster against the other genes in the sample list. It highlights the classes that contributed most to the formation of the cluster. The third measure, "Complete log(p)" (denoted by C.log(p)), compares a single cluster against the other genes of the sample gene list and reference. It takes into account both the contribution to the formation of a cluster and the over-representation in the sample list before clustering, and thus we use it for reporting the contents of a cluster. C.log(p) is partly dependent on the preceding clustering, and thus can report some classes that are not over-represented in the whole user given sample gene list, which we are aiming to analyze. Therefore, such hits are filtered by excluding the classes with weak O.log(p) from the report. Similarly, classes that have not contributed to the formation of the analyzed cluster are removed by discarding the classes that do not show even slight over-representation with S.log(p). As a result of filtering, the remaining classes are over-represented in both the analyzed cluster and in the original sample list. In this description, only O.log(p) gives statistically analyzable results because C.log(p) and S.log(p) are both based on the same data with the preceding clustering. Nevertheless, the latter two are suitable for highlighting the classes that are over-represented within the clusters. A more detailed description of the non-nested clustering scheme is given in the *Methods *section.

### Software implementation

In order to make the method applicable for others, we have developed an end-user program called GENERATOR (GENElist Research Aimed Theme-discovery executOR) for the Windows 2000/XP environments. It takes, as input, the sample and reference lists of genes that can be comprised of gene names or identifiers supported by GO database. The list of available species and allowable naming systems are described more in GENERATOR user manual [21] and in GO web site [22]. Alternatively, GENERATOR can be used to analyze existing binary data matrices like in-house created functional gene classifications or other similar binary data analysis problems consisting of sample and reference groups. The first outcome from the program is a non-nested hierarchical clustering tree, which shows the discovered gene sub-groups from the user given sample gene list. The content of each cluster is described by the two most over-represented classes. A more detailed analysis is also possible for each cluster by viewing the sorted list of over-represented classes or by viewing the clustered genes. The program can create multiple cluster trees, produce statistical evaluations for clustering divisions and single clusters, and provide flexibility in changing the parameters for clustering execution and visualizations. Results can be saved as graph figures and tab-delimited files describing different gene groups or class contents within them. These functions are further described in the program manual. GENERATOR will be updated twice in a year including the GO database within it and is freely available [21].

### Analysis with GENERATOR

#### Gene list from yeast under H_2_O_2 _stress

We have analyzed the data obtained from growing yeast deletion strains during oxidative stress [2]. Yeast deletion strains have deletions in genes not needed in normal growth conditions (non-essential genes). The research aims to find new genes and functionalities that are important for the cells to survive and grow in the presence of oxidative stress. We limit the analysis to the gene list obtained from hydrogen peroxide stress (H_2_O_2 _stress). This was used as a sample list for GENERATOR and it included 117 genes of which 109 were recognized by the GO database. The remaining 4589 non-essential yeast genes were used as a reference list of which 4115 were recognized by the database. The use of a whole genome as a reference here might cause some error in the results as it is natural to assume that different functional groups have different proportions of non-essential genes. The principal observation when analyzing the results as one group in the original article is the clear association with mitochondrion [2].

Clustering was done with 2 to 6 groups. In the first step the obtained clusters were analyzed against the other clusters using S.log(p) values to determine which functional classes contributed most to the formation of each cluster. The obtained graphical view is shown in figure 1A. The figure shows a cluster of ribosome genes that forms the clearest separate group (marked with I) and remains although the number of clusters changes from 2 to 6. The strong link between the different clustering results (thick lines showing correlations higher than 0.9) highlights this. Similarly, a cluster of genes with RNA associated function (marked with II) is clearly separated and is shown on several levels. Also, a small cluster of 'mitochondrial inner membrane' genes (marked with III), a cluster of genes with unknown function (marked with V), and a cluster associated with 'transcription regulation' and 'nucleus' (marked with IV) can be seen. All of these five clusters stay similar over many levels of the visualization despite the changing number of clusters and different random starting points. The whole cluster tree step was also replicated four times, each showing similar results. These replications are detailed below.

The previous information obtained by S.log(p) explains the clustering, but it does little to help understand the original sample list. This is due to the exclusion of the reference list from the analysis. For example, the previous results do not provide emphasis on mitochondrial functions although it is the most significant theme when analyzing the data as one group (see table 2). Figure 1A also presents 'molecular function unknown' class, although it is under-represented in the original sample list. Therefore, the second step of the analysis is to take the reference gene list into account. Here, classes are sorted with C.log(p) values and O.log(p) and S.log(p) are used as cut-offs to remove non-relevant information. The rationale of using the cut-offs and the purpose of the different values is discussed more in the *Methods *(*Description of the cluster contents*). This is also the default view of GENERATOR. The resulting graph is presented in figure 1B. Now the obtained view is different showing 'mitochondrial ribosome' cluster (I, previous ribosome cluster), 'tRNA ligase' cluster (II, previous RNA associated cluster), 'mitochondrial inner membrane' cluster (III) and 'transcription regulation' cluster (IV, previous transcription and nucleus cluster) and a 'mitochondrial genome maintenance' cluster (V, previous cluster of unknown genes). The clusters are the same as the ones shown in the figure 1A but now each one of the clusters shows the functional classes, over-represented in the original sample list, that are associated with the clustered genes. The over-represented classes for clustering with 5 clusters from figure 1 are shown in table 1. In order to see how robust the results are, the non-nested hierarchical clustering was replicated four times. The replications are in figure 2 and show that similar clusters can be obtained with each.

**Table 1:** Results from GENERATOR with H_2_O_2 _dataset using five clusters. The table shows the reported classes for five clusters from GENERATOR clustering shown in figure 1. The three over-representation values described in *Methods *section and figure 5 are reported for each class. Results are also compared to graphical output from SGD GO term finder (figure 8 [see Additional file 3]). Abbreviations in this column are: MF, molecular function; CC, cellular component; BP, biological process. Classes reported as 'missing' were not observed in the SGD GO term finder graphs. A more detailed view of the data is available in table 5 [see Additional file 5]. Classes with *S.log(p) *< 1 (marked with -) are not included to analysis although they are still shown here.

**CLUSTER**	**FUNCTIONAL CLASS**	**C.log(p)**	**O.log(p)**	**S.log(p)**	**SGD**
**I**	organellar ribosome	46.7	26.1	21	CC
	mitochondrial ribosome	46.7	26.1	21	CC
	mitochondrial matrix	41.6	30.5	14.9	CC
	Ribosome	36.9	14.5	23.7	CC
	structural constituent of ribosome	35.9	14.8	22.3	MF

**II**	RNA ligase activity	13.3	6.68	6.64	MF
	tRNA ligase activity	13.3	6.68	6.64	MF
	ligase activity, forming aminoacyl-tRNA and related compounds	13.3	6.68	6.64	MF
	ligase activity, forming carbon-oxygen bonds	13.3	6.68	6.64	MF
	ligase activity, forming phosphoric ester bonds	13	6.42	6.64	MF

**III**	mitochondrial membrane	13.9	3.66	11	CC
	inner membrane	13.1	3.99	9.6	CC
	mitochondrial inner membrane	13.1	3.99	9.6	CC
	Mitochondrion	8.91	40.1	1.03	-
	respiratory chain complex III	8.03	4.32	3.74	CC

**IV**	transcription regulator activity	12.3	2.52	11.5	MF
	nucleobase, nucleoside, nucleotide and nucleic acid metabolism	8.99	2.15	7.51	BP
	mediator complex	8.97	5.15	3.88	Missing
	general RNA polymerase II transcription factor activity	7.94	4.16	3.88	MF
	DNA-directed RNA polymerase II, holoenzyme	7.81	4.04	3.88	Missing

**V**	mitochondrial genome maintenance	7.22	5.97	2.02	Missing
	mitochondrion organization and biogenesis	6.74	4.06	3.05	Missing
	mitochondrial chromosome	4.23	3.18	1.05	- (CC)
	soluble fraction	3.1	2.52	1.09	-
	helicase activity	3	3.35	0.79	-

**Table 2:** Comparison of sorted class list against GENERATOR clustering with H_2_O_2 _dataset. The table shows the sorted list of over-represented functional classes for H_2_O_2 _dataset. Only the 25 best scoring classes are shown to limit the size. Columns indicate the obtained log-p-values (O.log(p)), class names and its rank in the list, and the corresponding GENERATOR cluster number, if the class was included into the obtained GENERATOR result. Notice that the most of the functional classes are associated with mitochondrial ribosome proteins. Detailed results are shown in table 7 [see Additional file 7].

**Rank**	**Class name**	**O.log(p)**	**In cluster**
1	Mitochondrion	40.06	first level
2	mitochondrial matrix	30.45	II
3	organellar ribosome	26.09	II
4	mitochondrial ribosome	26.09	II
5	protein biosynthesis	21.69	II
6	organellar large ribosomal subunit	18.61	II
7	mitochondrial large ribosomal subunit	18.61	II
8	macromolecule biosynthesis	15.6	not reported
9	structural constituent of ribosome	14.84	II
10	Ribosome	14.53	II
11	Biosynthesis	13.15	not reported
12	structural molecule activity	12.87	not reported
13	ribonucleoprotein complex	12.52	II
14	protein metabolism	12.34	not reported
15	Metabolism	11.75	not reported
16	large ribosomal subunit	11.33	not reported
17	organellar small ribosomal subunit	7.94	not reported
18	mitochondrial small ribosomal subunit	7.94	not reported
19	aerobic respiration	7.3	IV
20	cellular respiration	7.03	not reported
21	Cytoplasm	7.02	not reported
22	RNA ligase activity	6.68	III
23	tRNA ligase activity	6.68	III
24	ligase activity, forming aminoacyl-tRNA and related compounds	6.68	III
25	ligase activity, forming carbon-oxygen bonds	6.68	III

Analysis of the results in figures 1 and 2 (result summary shown in tables 1 and 5 [see Additional file 5]) shows that within the group of genes that first seem homogeneous, there are sub-groups differently associated with the mitochondrial functionality. The strongest feature in the obtained results is the group of mitochondrial ribosome clusters that stays similar whether clustering from 2 to 6 clusters. Analysis of this cluster actually reveals that there are two genes (YNR036C and YPL183W-A) that are reported as hypothetical mitochondrial ribosome proteins. The fact that the mitochondrial ribosome proteins are strongly over-represented in the dataset support the notion that they are mitochondrial ribosomal proteins.

One small group, not mentioned in the original analysis [2], is the group of tRNA ligases (cluster II). Although this group only includes 6 members, its O.log(p) was 6.64 making the over-representation significant. A more detailed analysis reveals that the genes in question are mitochondrion associated tRNA ligases and one of them is a hypothetical mitochondrial tRNA ligase. Again its importance for the growth of yeast cells in oxidative stress further confirms its association with mitochondrial function. The rank of these ligase associated categories starts at 23 in the sorted class list for the original sample gene list (see table 2) and therefore this group can go easily unnoticed if the sample list is analyzed without clustering. The rest of the cluster II (in fig. 1, when using five clusters) includes proteins that link to RNA processing and to translation, for example, NAM1, two mitochondrial elongation factors, and YDR194C.

Cluster V shows 'mitochondrion organization' and 'genome maintenance' (5 and 7 genes, with O.log(p) 4.06 and 5.97) but the analysis of the cluster content shows no clear common theme. Instead, most of the genes have no known function, and therefore this cluster does not seem to contribute to the analysis. Indeed the unknown function was associated to this cluster in figure 1A. A separate cluster of unknown genes is an expected behavior for our method as these genes have highly different GO classification profiles from the known genes. We have also observed it regularly with other datasets. Still, this cluster was able to highlight the small group of genes associated with mitochondrion genome maintenance.

Cluster IV shows nucleus-associated functionalities ('transcription regulator activity', 'regulator complex', 'general RNA polymerase II transcription factor activity'). When the actual cluster content is analyzed, the cluster includes: RNA polymerase II holoenzymes, transcription factors, and transcription regulators. This cluster of genes was unexpected and seems to show a link from nucleus driven functionalities to mitochondrial functionalities. Clusters II and IV show nicely mitochondrion linked functions elsewhere in the cell, but at the same time these groups are harder to detect when analyzing the data as one group (see tables 2 and 7 [see Additional file 7]). In summary, GENERATOR has shown that within the mitochondrion associated gene list, the main members are mitochondrion ribosomal proteins, mitochondrion membrane genes, tRNA ligases, unknown genes, and genes associated with transcription regulation.

#### Gene list from drug treated yeast

Another dataset that was analyzed includes the gene expression differences in yeast during itraconanzole treatment, a drug known to affect sterol biosynthesis and normal growth [20]. Both up and down regulated genes were used for the analysis. These contained 255 genes of which 248 were recognized by the GENERATOR GO database. The remaining 6102 non-regulated yeast genes constituted the reference list of which 5369 were recognized by our database. When the obtained gene list is analyzed normally with the sorted class list, the most significant feature observed is the massive over-representation of the 'aminoacid biosynthesis' and related functional classes (table 4).

**Table 4:** Comparison of sorted class list against GENERATOR clustering with itraconanzole dataset. The table shows the sorted list of over-represented functional classes for itraconanzole dataset. Only the 25 best scoring classes are shown to limit the size. Columns are same as in table 2. Notice that most of the functional classes are associated with amino acid biosynthesis. Detailed results are shown in table 8 [see Additional file 8].

**Rank**	**Class**	**O.log(p)**	**In cluster**
1	amino acid biosynthesis	19.07	I
2	amine biosynthesis	18.08	I
3	carboxylic acid metabolism	14.77	I
4	organic acid metabolism	14.77	I
5	amino acid metabolism	14.70	I
6	amino acid and derivative metabolism	13.75	I
7	amine metabolism	13.02	I
8	arginine biosynthesis	11.28	I
9	steroid metabolism	8.89	II
10	nitrogen metabolism	8.79	not reported
11	urea cycle intermediate metabolism	8.66	not reported
12	arginine metabolism	8.66	not reported
13	Biosynthesis	8.52	I, II
14	transaminase activity	8.32	not reported
15	transferase activity, transferring nitrogenous groups	8.32	not reported
16	glutamine family amino acid biosynthesis	8.16	not reported
17	sterol metabolism	8.02	II
18	glutamine family amino acid metabolism	7.99	I
19	sterol biosynthesis	7.77	II
20	steroid biosynthesis	7.69	II
21	ergosterol biosynthesis	6.22	II
22	ergosterol metabolism	6.22	II
23	aromatic compound metabolism	5.85	not reported
24	branched chain family amino acid metabolism	5.61	not reported
25	cyclohydrolase activity	5.50	not reported

Similar to the previous analysis, two steps were used and the classes that contributed most to the clustering were monitored first. The results show the 'carboxylic acid biosynthesis' associated cluster (marked with I) a cluster associated with 'cellular process' class (III); a 'macromolecule biosynthesis' associated cluster (II); and a cluster associated with unknown functionality (IV). With a larger number of clusters, 'nucleobase metabolism' and 'transcription' associated cluster (V) can be seen.

When the clustering view is changed to show the over-representation reported by C.log(p) (figure 3B), the previous clusters obtain different annotations (result summary shown in tables 3 and 6 [see Additional file 6]). This analysis step was again repeated four times to see how similar the results remained (figure 6 [see Additional file 1]). Cluster I, that showed in fig. 3A carboxylic acid biosynthesis, is now associated with amino acid and carboxylic acid biosynthesis. It forms the most stable cluster and it is seen regularly on several clustering levels also in the replications. Cluster II (macromolecule biosynthesis) is now associated with steroid biosynthesis. Genes in the cluster represent sterol biosynthesis associated functions and other macromolecule biosynthesis functions (for example synthesis of phospholipids). Steroid synthesis is a known target of the drug and that it is now nicely separated from other functionalities that are likely more secondary responses to the drug. Third, a regularly seen cluster is one enriching the plasma membrane and cell wall associated functionalities (III). The genes in this cluster show many membrane associated functions, like transporting activities. Unexpectedly, another cluster, associated with cell wall (cluster IV) can be regularly observed. A detailed analysis of these clusters still reveals that they are different. Cluster III is associated strongly with 'plasma membrane' and 'cell wall'. The other cell wall cluster (cluster IV) is more connected to unknown cellular component than to cell wall and the connection to cell wall is also very weak. Even a slight raise of the cut-off for S.log(p) would filter this link. A more detailed analysis of the cluster IV reveals that 55 out of the 65 genes in the cluster have biological process unknown. Moreover, molecular function is unknown for 58 of these genes. Therefore this cluster does not contribute to the analysis of the gene list. Cluster V does not seem as stable as the earlier clusters. Still, it is observed in most of the replications (figure 6 [see Additional file 1]). It groups together genes associated with nucleobase metabolism and transcription. Detailed analysis shows transcription factors associated with regulation of transcription from the Pol II promoter. Among these genes, some of them are reported to be important for drug resistance (YLR266C, YCR106W) and to stress response (YFL031W, YMR037C) and to two associated with copper uptake (YGL166W, YMR021C). In summary, we observed with GENERATOR an amino acid biosynthesis associated group, steroid and lipid biosynthesis associated group, a group of unknown genes, and genes associated to membrane and transport.

**Table 3:** Results from GENERATOR with itraconanzole dataset using five clusters. The table presents the reported classes from GENERATOR clustering with six clusters shown in figure 3. Columns and abbreviations are the same as in table 1. A more detailed view is presented in table 6 [see Additional file 6]. We omit the classes with S.log(p) smaller than 1 from the comparison with SGD (-). One outlier cluster is also omitted (shown in more detailed view).

**CLUSTER**	**FUNCTIONAL CLASS**	**C.log(p)**	**O.log(p)**	**S.log(p)**	**SGD**
**I**	amino acid metabolism	51.48	14.7	38.32	BP
	carboxylic acid metabolism	50.64	14.77	36.64	BP
	organic acid metabolism	50.64	14.77	36.64	BP
	amino acid and derivative metabolism	50.36	13.75	38.32	BP
	amino acid biosynthesis	49.98	19.07	31.6	BP

**II**	steroid metabolism	19.83	8.89	11.36	BP
	lipid biosynthesis	17.88	5.24	14.07	BP
	lipid metabolism	17.87	5.05	13.81	BP
	sterol metabolism	17.3	8.02	9.61	BP
	steroid biosynthesis	16.95	7.69	9.61	BP

**III**	plasma membrane	9.08	2.58	7.5	Missing
	cell wall (sensu Fungi)	4.18	4.37	1.43	CC
	cell wall	4.18	4.37	1.43	CC
	external encapsulating structure	4.18	4.37	1.43	CC
	structural constituent of cell wall	3.97	2.23	1.8	Missing

**IV**	cell wall (sensu Fungi)	2.4	4.37	0.54	-
	cell wall	2.4	4.37	0.54	-
	external encapsulating structure	2.4	4.37	0.54	-
	acid phosphatase activity	1.25	3.1	0.22	-

**V**	specific RNA polymerase II transcription factor activity	10.18	3.24	7.48	MF
	nucleobase metabolism	3.26	2.85	1.52	Missing
	purine base metabolism	2.56	2.58	1.09	Missing
	aromatic compound metabolism	2.54	5.85	0.72	-
	heterocycle metabolism	2.22	2.65	1.02	Missing

### Comparison with competing methods

#### Sorted class list

GENERATOR was also compared to existing methods. One of the simplest ways of analyzing a gene list is to take it as one single group, analyze how over-represented different classes are, and to report the results as a sorted list. Sorting is based on the p-values calculated for the observed over-representation in order to show the best results at the top of the list. This method does not take into consideration the heterogeneity in the list, but otherwise it is similar to analysis done with each of the GENERATOR clusters. Actually, the first level of the GENERATOR cluster tree graph does this analysis. Therefore we compared GENERATOR clustering to the sorted class list using the results from the first level. We changed the default settings so that the number of reported functional classes was not limited.

The comparison used the two previously analyzed data sets. The results from sorted class list were compared to GENERATOR clustering summaries shown in tables 1 and 3. When the number of classes was limited only by the p-value, an immediately observed drawback of the sorted list method was the amount of information (number of classes) obtained. For the H_2_O_2 _dataset, we obtained 75 classes and for itraconanzole 76 with -log(p-value) > 2 (55 and 43 with -log(p-value) > 3). The resulting sorted lists are shown in tables 7 [see Additional file 7] and 8 [see Additional file 8]. This can be corrected by raising the cut-off for the included genes. This is also reasonable as we have not used here any correction for increased risk of false positives due to multiple testing. Strong filtering with p-values or limiting the number of reported classes leaves the most over-represented functional classes. In the example datasets, the most over-represented functional classes were all associated with the same gene group. With H_2_O_2_, the first 18 functional classes were associated with mitochondrial ribosome proteins (see table 2). With itraconanzole, the first 19 classes (except classes 9 and 17) show functions associated with amino acid biosynthesis (see table 4).

When the GENERATOR results were compared to a sorted class list, many classes were omitted from the results. With default settings, GENERATOR shows at maximum ten classes for each cluster in the output text file. This filters out the repetitive occurrences of functional classes associated with the same gene group. In the H_2_O_2 _dataset, classes like macromolecule biosynthesis, protein metabolism, and large ribosomal subunit were excluded in this way. This seems acceptable as many similar classes are shown in the results by cluster I. The omitted classes can be still viewed with the sorted list available for each cluster. Another group of classes that are not reported by GENERATOR with H_2_O_2 _were very broad classes, such as intracellular, cell, or physiological process. These contribute very little information to the analysis. Similar observations were also seen with the itraconanzole dataset, where many amino acid biosynthesis associated classes were excluded from GENERATOR clustering results. As an exception, itraconanzole showed some broad classes in the results (plasma membrane, cell wall).

#### Direct acyclic graph

Another way to analyze the obtained gene list is to map the over-represented functional classes into a tree like structure that is behind the GO classes and visualize the results as a graph structure. The benefit to the sorted list presentation is that the hierarchical structures are now visible, highlighting the over-represented functional classes occurring repetitively in the same part of the GO graph. Also, if there are different branches showing over-represented functional classes in the GO structure, they are clearly separated. The major drawback is the large size of the obtained visualization. The graph obtained from AMIGO server [23] using the whole list of over-represented classes from H_2_O_2 _dataset was simply too large for analysis (figure 7 [see Additional file 2]). Instead we selected a graphical output from GO term finder at Saccharomyces Genome Database [8] for comparison. The GO term finder adds color coding to show which of the classes showed strongest over-representation. It also tries to make the obtained graph smaller by discarding some branches. As the graph for each ontology is obtained separately, we combined the obtained three graphs to the same picture for a better view. We used GENERATOR clustering summaries shown in tables 1 and 3 for comparison.

In order to compare the obtained GO graphs with the GENERATOR results, we flagged each class that was reported significant if it was included in the GENERATOR result table (figures 8 [see Additional file 3] and 9 [see Additional file 4]). We first observed, in the comparison, that the graphs obtained from SGD GO term finder are still large for analysis. Also, the important features are scattered over three graphs, in comparison to the single table from GENERATOR. It was observed that some classes in the H_2_O_2 _data were not shown in the SGD GO graph even though their log-p-value results were highly significant (tables 1 and 3, classes marked as 'missing'). Some of these classes were: aerobic respiration (O.log(p) 7.3), cellular respiration (7.03), and mitochondrial genome maintenance (5.97). This might be an artifact caused by the limited size of the GO graph. SGD graph, on the other hand, showed classes that were not reported by GENERATOR. These classes were the same classes discussed when comparing GENERATOR with the sorted lists. Some of the differences between the results might be explained by the usage of binomial test for calculating significance of the functional classes in GO term finder. It should be noted that the Fisher's exact test used by GENERATOR is a more correct method [8], although we observed similar p-values with both methods. Also the whole genome is always used as a population by GO term finder, which might also cause bias in the results with some datasets (see analysis of H_2_O_2 _dataset above).

#### Comparison to GOToolBox

During the preparation of this manuscript, we also observed another method that performs similar GO clustering. GO-Proxy in GOToolBox [19], takes the user given sample gene list, creates the GO classifications for each gene and clusters the obtained matrix by using czekanowski-dice distance and hierarchical clustering. The reported clusters (called classes) are selected from the different levels of tree with two parameters, defined by the user. One parameter defines how similar genes have to be inside the cluster and the other defines the minimum size for the cluster. The principal difference between the methods is that GENERATOR (with default parameters) reports only the GO-classes that display over-representation in both the original sample gene list and in the obtained cluster, whereas GOToolBox concentrates its analysis to the obtained cluster. Also, GENERATOR gives an overview of the clustered data with visualization.

In the analysis for H_2_O_2 _and itraconanzole datasets, GOToolBox, with default parameters, created more and smaller clusters when compared to GENERATOR (tables 9 [see Additional file 9] and 10 [see Additional file 10] for results with each ontology). The cluster number is probably larger because the same clusters with minor changes are selected from different levels of the hierarchical clustering tree which causes repetition in the results. The small clusters in GOToolBox results tend to give a scattered view of the data but could be also useful when analyzing details from the obtained gene list. However, by setting a larger minimum cluster size they can be filtered. With larger clusters GOToolBox reported nonspecific functional classes like cellular process, cell, or metabolism in addition to the same GO-classes that were previously reported by GENERATOR (mitochondrial ribosome classes, tRNA classes etc.). With the default settings, GOToolBox found also some small clusters that were not reported by GENERATOR (clusters associated with 'abiotic stress', 'RNA metabolism' etc.). These clusters were quite small and the most associated functional classes did not show any over-representation in the original sample list (see table 7 [see Additional file 7]) as GOToolBox does not filter the results with O.log(p). GENERATOR could be also run with a larger maximum cluster number in order to obtain similar smaller clusters.

## Discussion

We have presented a method that groups a user provided gene list into functionally dissimilar gene clusters. The grouping is done with varying numbers of clusters, which are used to create a tree-like graphic visualization. Despite the emphasis on clustering, our method also analyzes the gene list as a single entity (result with one cluster). The obtained graph presents the main output of the method showing the most important simultaneous gene groups that occur in the data in a single figure. The graph can be created multiple times to see how stable it remains when different random initializations are used for clustering. Our results from clustering replications show that the most visible gene groups remain, thus increasing confidence in the method.

There are two alternative methods previously used to obtain an overview of the over-represented functional categories. Methods like EASE analyze the gene list as one entity and output the functional categories as a sorted list according to the significance of the over-representation. Other methods, like SGD GO term finder, give the over-represented functional categories as a directed tree-like graph by using the hierarchical structure of GO. Graph methods create a much more complex representation with the danger of overwhelming the user with unimportant details. The sorted list gives an impression of a homogenous gene group. As an example, we showed the results from SGD GO term finder, AMIGO visualization, and the sorted list of functional classes for the gene list as one entity. These methods do not group the gene list before analyzing it. A positive unexpected observation was that results from the other methods seemed more informative after we marked them with the corresponding GENERATOR clusters. For example classes in a sorted list can be marked according to which cluster they belong to (see tables 2 and 4). Marking the corresponding clusters enables the opportunity to combine GENERATOR clustering results and results from other methods.

We also compared the GENERATOR results to another gene clustering tool, GOToolBox. The principal difference in methods is that GENERATOR provides the cluster description by using filtering procedure which discards the GO-classes with no over-representation in the original sample gene list and with weak association to the genes of the cluster. GENERATOR includes also visualization for viewing the optional clustering results. Despite the differences we were able to obtain also similar GO-classes with both methods when analyzing the H2O2 and itraconanzole datasets.

Since partitive clustering has an inherent weakness in the initialization, we present a novel solution. Instead of selecting a single clustering number, we monitor the results with a range of clustering numbers. As a result, we obtain correlations between the clusters that highlight those features that can be obtained even though the cluster number would change. The replication of the whole cluster tree visualization was done in order to further highlight those features that are conserved. It should be noted that these ideas could also be used with other clustering applications. Similar work was done by Heger and Holm [24] by replicating NMF many times and looking for the conserved features in the obtained matrix factorizations and by Brunet et al [25] where optimal cluster number was selected by replicating NMF clustering many times.

We analyzed the obtained clusters by concentrating on those functional categories that were over-represented in the cluster when compared to the rest of the gene list and also in the original list of genes when compared to a reference list of genes. If the over-representation in the cluster only would be monitored, the obtained cluster would be well explained, but the drawback would be that the obtained categories could at worst be such that they were under-represented in the original gene list and therefore produce erroneous conclusions. If the over-representation in the original list would be only monitored, the clustering would not be informative to the analysis. The current way of combining these two over-representations highlights those features that are common between the original list and the obtained cluster. As the data is grouped to separate clusters, each of them will represent different features from the list of over-represented functional classes for the original gene list. The reporting method therefore separates those functional categories from the original gene list that are not associated to the same genes and groups together those functional categories from the original gene list that are connected to the same genes. A good example of genes that were associated to the same function were the members of the same protein complex that were often seen as a separate cluster.

The selection of the reported functional categories requires the definition of the cut-off for the significant over-representation. Here the threshold was purposely selected to be liberal (p-value < 0.01, O.log(p) > 2.0). This is known to be too weak a threshold when the analysis includes multiple testing as it increases the possibility of the false positives. Therefore the emphasis was placed in the later analysis on those functional categories that showed clearly stronger over-representation than what the cut-off was and the p-values larger than 0.001 were monitored with caution. Similarly we also discarded classes with S.log(p) < 1 from our analysis. The P-value borders could be selected more precisely by doing repetitive testing with a similar sized sample list with randomly selected members (permutation analysis). The evaluation of the results using runs with randomized samples from the analyzed data is one of the planned additions to the GENERATOR software.

The associated software uses a reference list to calculate over-representation for the original cluster. Although the whole genome for the organism could be used, the reference list will ensure that the biases towards some functional groups in the test situation do not affect the analysis.

The method demonstrates that a drugs primary target can be identified within a separate group among different regulated genes and different cellular functions. Work shown here was done with yeast allowing the use of detailed annotation of the yeast genome. Still, we have also obtained encouraging results from human cell line and *C. elegans *gene expression datasets (manuscript in preparation). As more information is being gathered from the gene functions, this method should be able to perform even better. Nonetheless, accuracy in the used gene annotations is the weak link for our method. This should not necessarily be a hindrance, as the randomly classified genes should distribute randomly also among the observations. Another limitation is the recognition of the analyzed genes. Gene identifiers can be problematic when working with different naming systems that originate from various databases or high throughput methods, like gene chips. These are also the problems faced by other methods.

The presented software includes the possibility of using it also with binary matrices. The reference group can be given as a binary matrix or as a vector that represents a number of members of each category and also the size of the reference group. This should enable the analysis of other similar binary data sets, like SNP datasets, word occurrences in abstract texts etc. These are being currently tested as future applications.

## Conclusion

We have presented an analysis method and associated software, GENERATOR, for analysis of large gene lists. Our aim has been to fulfill the need for an analysis tool to separate and identify functional gene groups from gene lists that would otherwise be difficult to find. The method should be useful especially as larger and more complex gene lists are produced due to the increased use of high throughput genomic methods.

## Methods

### Data representation

**Figure 4:**
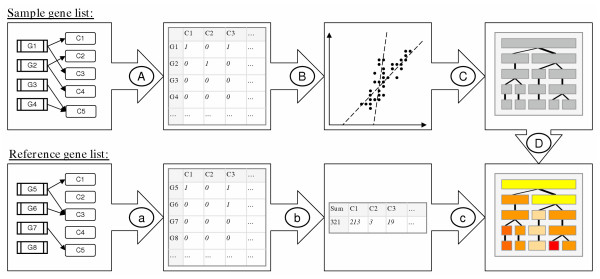
**Flow diagram of the method**. The gene associations with the GO functional classes in the sample and reference gene lists are transformed into binary matrices (A and a) and a sum vector (b). The sample set is clustered with NMF based method (B) into a varying number of sub-groups producing a non-nested hierarchical tree (C). Contents of the clusters are described with the over-represented classes within them (c and D).

The associations between genes and functional classes in the sample and reference gene lists must be represented as a binary matrix to enable the analysis (see figure 4, steps A and a). As functional classes, we use annotations from the April 2004 delivery of Gene Ontology (GO) database [14]. GO includes three principal sub-hierarchies, representing *biological processes*, *cellular components *and *molecular functions *for a gene. We combine the information from all these three hierarchies in the clustering process.

The gene and functional class associations are transformed into binary matrix where rows represent genes and columns represent classes. Association between gene and class is denoted by one and lack of association with zero in a matrix cell. In addition to directly associated classes, a gene is also denoted to associate with its ancestors in the hierarchical GO structure to assure maximal information for analysis. The obtained matrix for sample gene list is inputted for the clustering process whereas the matrix for the reference list is summed into an occurrence vector (figure 4, step b) which is used later for analyzing the over-represented classes within the obtained clusters.

### Clustering technique for binary data

A binary matrix is used as data when clustering the user given sample gene list into a fixed number of groups (see figure 4, step B). Many traditional clustering methods obtain weak results with such data due to its non-continuous nature (see for example [26,27]) and the small proportion of non-zero entries (sparse matrix). Therefore we have selected a clustering procedure based on Non-negative Matrix Factorization (NMF, [15]) that has shown good performance with binary data in the 'topic finding' literature ([15,28]).

NMF aims to reduce the dimensions of multivariate data by factorization *X *≈ *WH *where *X *represents the binary matrix obtained from the associations of *n *genes and *m *classes in the user given sample gene list. Given the fixed number for *r*, two matrices *W *(size *n *× *r*) and *H *(size *r *× *m*) are produced as a result, representing the input data *X *in compressed form of *r *factors. The first of the matrices describes the loadings of the genes on *r *factors and is further used in clustering. In the clustering process, the genes are deposited into clusters by using a winner-takes-all approach that finds the factor with the highest loading for each gene from matrix *W*. The relation between the highest loading and sum of all loadings is used to measure the fitness of a gene in a cluster. In the visualization (see next chapter) the fitness is used to present genes in a sorted order for each cluster. More detailed descriptions concerning clustering binary data with NMF are given in [15,28]. We use the NMF algorithm presented in [29] which minimizes the least squares error (LSE) between the input data and resulting factorization.

### Non-nested hierarchical clustering scheme

The core of the proposed method is a non-nested hierarchical clustering tree, which is shown in figure 4, step C. There the user given sample gene list is repeatedly clustered into *r *number of groups, where *r *grows gradually from two into a user given number. Each partitive clustering is executed from a random starting initialization using NMF, producing an independent division level to the visualization. The levels are placed consecutively in the growing order of *r *starting from *r = 1*, which represents the sample gene list without any clustering. In the visualization, each level is shown with a bar of constant size that is split into *r *sections. Each section represents a single cluster, the size of which is indicated by the width of the section. Correlations between each cluster in level *r *and all clusters of previous level *r-1 *are calculated by comparing cluster memberships of genes with a correlation measure between two binary classifications presented in [10]. The strongest correlation for each cluster is denoted by a line between the corresponding clusters. The width of the line indicates the magnitude of the correlation. The lines between the first and second levels present only the proportions of the genes, as the binary correlation with the first level can not be defined. Together the edges and sections form a non-nested hierarchical tree that visualizes the underlying heterogeneity in the gene and class association data.

### Description of cluster contents

We have developed a procedure for describing the contents of gene clusters (figure 4, steps c and D) resulting from the non-nested hierarchical clustering scheme introduced above. There, a combination of three measures is applied to find informative classes by studying their over-representation in the sample gene list with and without clustering. By definition, the over-representation means a greater frequency of classes in the collected set of genes than in the rest of the population. A robust way to test this is the calculation of p-values from a hypergeometric distribution with Fisher's test [16,17], that we apply. Classes with low p-values are highly over- or under-represented in the gene set and thus interesting. Nevertheless, the significant p-values are small numbers that are difficult to handle and visualize. They neither distinguish the over- and under-represented classes. Thus, we use signed logarithmic transform of p-value introduced before [10] which has negative or positive sign depending on the under- or over-representation and suitable scale for visualization.

**Figure 5:**
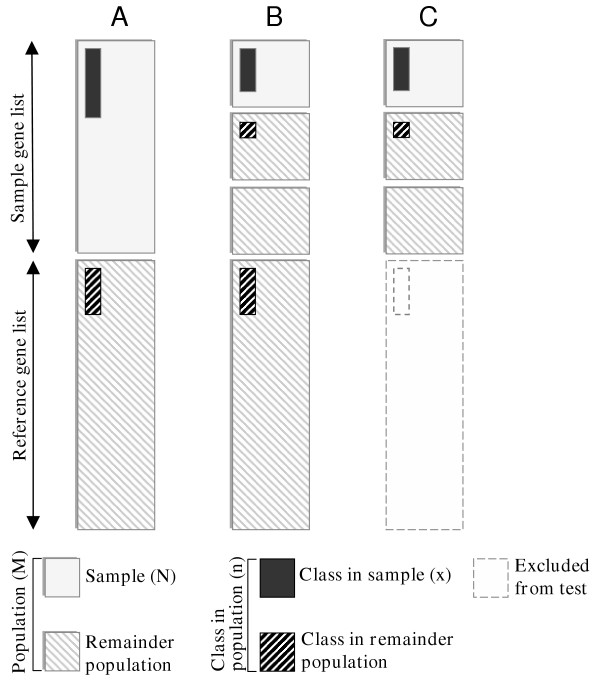
**The measures for studying over-representation of classes**. Over-representation of classes is measured by using A) the whole sample gene list as a sample and the reference gene list as a remainder population, *O.log(p)*; B) a single cluster as a sample and the rest of the sample gene list and the reference gene list as a remainder population, *C.log(p)*; and C) a single cluster as a sample and the rest of the sample gene list as a remainder population excluding the reference gene list, *S.log(p)*. In each situation, Fisher's exact test *f(x, M, n, k) *[16] is used to determine the over-representation. *O.log(p) *presents the original over-representation of sample gene list without clustering. *C.log(p) *highlights the classes that are over-represented in the original sample gene list and in individual cluster. *S.log(p) *reports the contribution to the formation of cluster structure.

In our method, we study the over-representation for multiple purposes. We calculate the p-values for each biological class (description in figure 5):

A) From original sample gene list without any clustering using user given reference gene list as a rest of the population. This is denoted by *O.log(p)*.

B) From each individual cluster using other clusters of sample gene list and reference gene list as a rest of the population. This is denoted by *C.log(p)*.

C) From each individual cluster using other clusters of the sample gene list as a rest of the population and excluding the user given reference gene list. This is denoted by *S.log(p)*.

In the default view of GENERATOR, these measures are used to show the over-represented functional classes in the clustering tree. In each cluster description, the basic over-representation measure C.log(p) is used to sort the classes. As C.log(p) is dependent on the clustering, it ranks high in some classes that are over-represented when measured from the cluster, but not over-represented when measured from the sample gene list without clustering. This is caused by the clustering process, when for example a tight group of genes is associated with the classes that are under-represented in the non clustered list. Since we aim at interpreting the whole list, such classes would be misleading and have to be removed. Therefore we filter them by using O.log(p), which is fully independent on the clustering. Another problem is that C.log(p) can rank high in some classes that have not contributed to formation of the analyzed cluster. These classes are under-represented in the cluster when comparing only to the rest of the sample gene list. Still they are so strongly over-represented in the whole sample gene list that C.log(p) shows over-representation. As these classes are uninformative for the analysis of the cluster, we filter them by using S.log(p). By default, the classes with O.log(p) < 2.0 or S.log(p) < 0.0 are filtered, although we encourage also the use of stricter cut-offs like 3 and 1, which we have found to work better especially with S.log(p). In the non-nested tree visualization, the two best classes from this filtered list are shown to describe cluster contents, and the longer list is available through the user interface (see *Software Implementation *in *Results*). The cluster is coloured according to the C.log(p) value of its most over-represented class with the strongest red for largest over-representation.

### Analysis protocol

We study two clustering views for each data set in our analysis. In addition to previously discussed default view, which shows the over-represented classes in the clustering tree, we first study the cluster formation. For that, we sort the classes by S.log(p) within the clusters, which excludes the reference gene list and fully concentrates on clustered data. The outcomes from this setting are shown in fig. 1A and fig. 3A in *Results*. Similarly, the outcomes from the default view are shown in fig. 1(B) and fig. 3B. In our manual analysis (results shown in tables), we further filtered the results from default view by emphasizing the classes with O.log(p) > 3.0 and S.log(p) > 1.0. In addition to two different clustering views, we also study the stability of our clustering scheme with both datasets in figures 2 and 6 [see Additional file 1]. This helps us to detect random and non-random outcomes in similar way as with single clustering levels explained above.

## List of abbreviations

GENERATOR GENElist Research Aimed Theme-discovery executOR

NMF Non-negative Matrix Factorization

GO Gene Ontology

SGD Saccharomyces Genome Database

MIPS Munich Information center for Protein Sequences

DAG Direct Acyclic Graph

LSE Least Squares Error

log(p) Signed 10 based Logarithmic Transform of p-value

O.log(p) Original log(p)

C.log(p) Complete log(p)

S.log(p) Sample log(p)

## Authors' contributions

The original idea of the approach was introduced by PT. Design of the method and the associated software was contributed equally by both PT and PP. PP implemented and tested the software and executed the analysis for biological datasets. GW reviewed the results and provided advice and guidance on improving the analysis and performing comparison to other methods. The obtained results were interpreted by PT.

## Supplementary Material

Additional File 5 **GENERATOR detailed class output for H_2_O_2 _dataset. **Table 5 The table presents a detailed view for the results obtained from GENERATOR with H_2_O_2 _data using five clusters. The table has two components. The first columns present the reported functional classes. The presented functional classes are selected with GENERATOR default view so that they highlight functional classes over-represented in the original list of genes and also in the cluster in question. In addition this part also shows the three reported p-values. These are the same as in the table 1. The table also includes number of class members in the cluster (inner size), number of class members in the original sample list (original inner size) and the number of class members in both sample and reference list (total size). These values can be used to analyse the proportion of the class that was included to cluster and the proportion of the class from the reported cluster. The reported classes can be also viewed as DAG with the included link to AMIGO www server. This enables the analysis of the hierarchy structure of the reported classes. The second part of table presents the list of genes for each cluster. Genes are presented in the sorted order so that the ones at the top of the list have always the strongest membership to the analyzed cluster. Click here for file

Additional File 7 **Detailed sorted class list for H_2_O_2 _dataset. **Table 7 The table presents a detailed sorted list of over-represented classes obtained with H_2_O_2 _dataset. Classes with p-value < 0.01 (O.log(p) > 2.0) are shown. Click here for file

Additional File 6 **GENERATOR detailed class output for itraconanzole dataset. **Table 6 The table presents a detailed view for the results obtained from GENERATOR with itraconanzole data using six clusters. One outlier cluster has not been taken in our analysis. Table has three main components. These are similar to the first three components in the table 5 [see Additional file 5]. Click here for file

Additional File 1 **Four replications of non-nested hierarchical cluster tree with itraconanzole dataset. **Figure 6 The figure shows four replications for the non-nested hierarchical clustering graph for itraconanzole dataset. We have marked the conserved gene clusters with the same Roman numerals as in figure 1. Notice again the conserved clusters observed over several levels in each cluster tree. Click here for file

Additional File 8 **Detailed sorted class list for itraconanzole dataset. **Table 8 The table presents a detailed sorted list of over-represented classes obtained with itraconanzole dataset. Classes with p-value < 0.01 (O.log(p) > 2.0) are shown. Click here for file

Additional File 2 **H_2_O_2 _dataset analysed with Amigo DAG View. **Figure 7 The figure presents the DAG view of all the reported classes shown in table 7 [see Additional file 7]. These classes had p-value < 0.01 (O.log(p) > 2.0). Figure was obtained from AMIGO server. The obtained figure was considered too complex for manual analysis. Click here for file

Additional File 3 **H_2_O_2 _dataset analysed with SGD DAG View. **Figure 8 The figure presents the three DAG tree figures obtained from SGD GO term finder with the H_2_O_2 _data. The reported classes are colour coded according the reported p-value. We have marked the classes that were reported by some cluster in GENERATOR results by adding the number of corresponding cluster. Note that many classes that were not reported by GENERATOR are usually close in the hierarchy to already reported classes. Click here for file

Additional File 4 **Itraconanzole data analysed with SGD DAG View. **Figure 9 Figure presents the three DAG tree figures obtained from SGD GO term finder using itraconanzole data. The reported classes are colour coded according the reported p-value. We have marked the classes that were reported by some cluster in GENERATOR results by adding the number of corresponding cluster. Note that many classes that were not reported by GENERATOR are usually close in the hierarchy to already reported classes. Click here for file

Additional File 9 **Summary of GOToolBox results with H_2_O_2 _dataset. **Table 9 The table shows a summary of H_2_O_2 _dataset analysis with GOToolBox. Results are shown separately for three sub-ontologies of GO. Columns show GOToolBox cluster number (Cluster nb), number of gene products within each cluster (Number of genes), and obtained class description (Class names). The raw output from GOToolBox is available in table 10 [see Additional file 10]. Click here for file

Additional File 10 **GOToolBox outputs from analysis with H_2_O_2 _and itraconanzole datasets. **Table 10 Files include the clustering results for H_2_O_2 _and itraconanzole datasets from GOToolBox. Click here for file
