# Supplementary figures and images for: Theme discovery from gene lists for identification and viewing of multiple functional groups
Source: BMC Bioinformatics. 2005 Jun 29;6:162. doi: 10.1186/1471-2105-6-162 (PMC1190153; doi:10.1186/1471-2105-6-162)

A

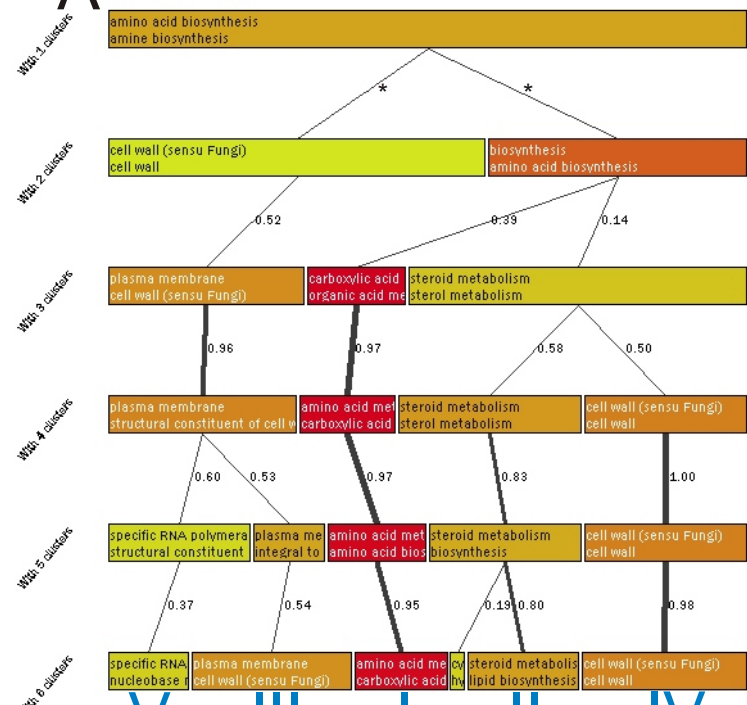

B

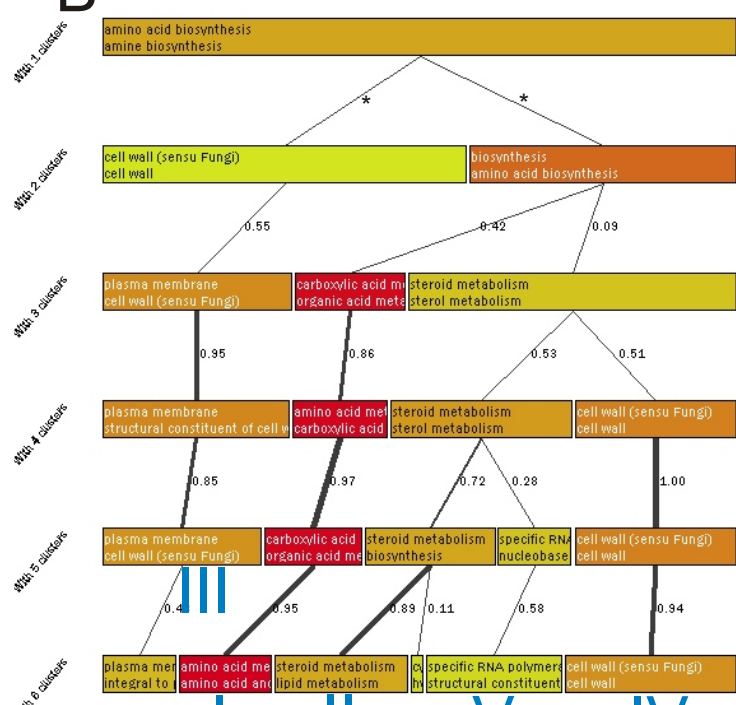

C

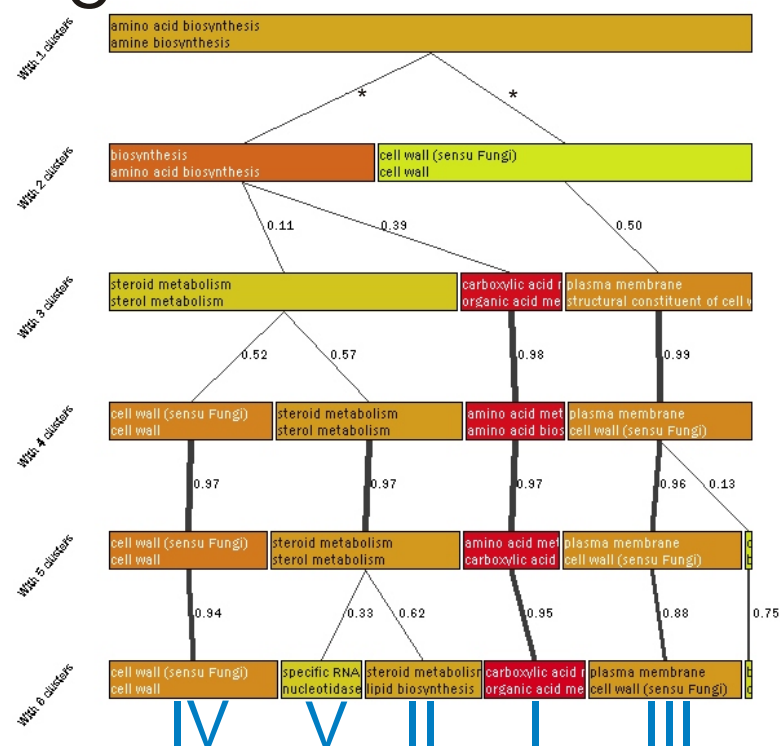

D

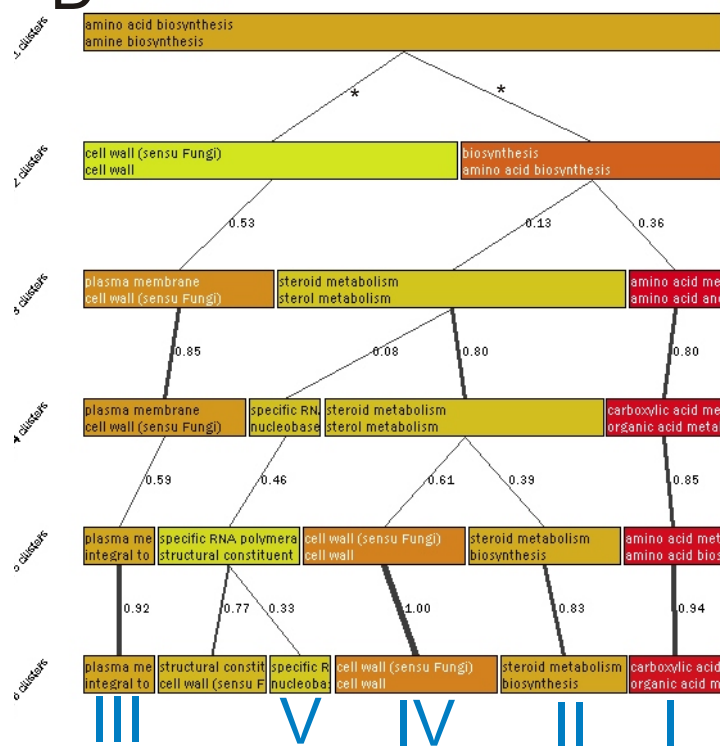

Supplement: Additional File 1 — Four replications of non-nested hierarchical cluster tree with itraconanzole dataset. Figure 6 The figure shows four replications for the non-nested hierarchical clustering graph for itraconanzole dataset. We have marked the conserved gene clusters with the same Roman numerals as in figure 1. Notice again the conserved clusters observed over several levels in each cluster tree. [file 1471-2105-6-162-S1.pdf]

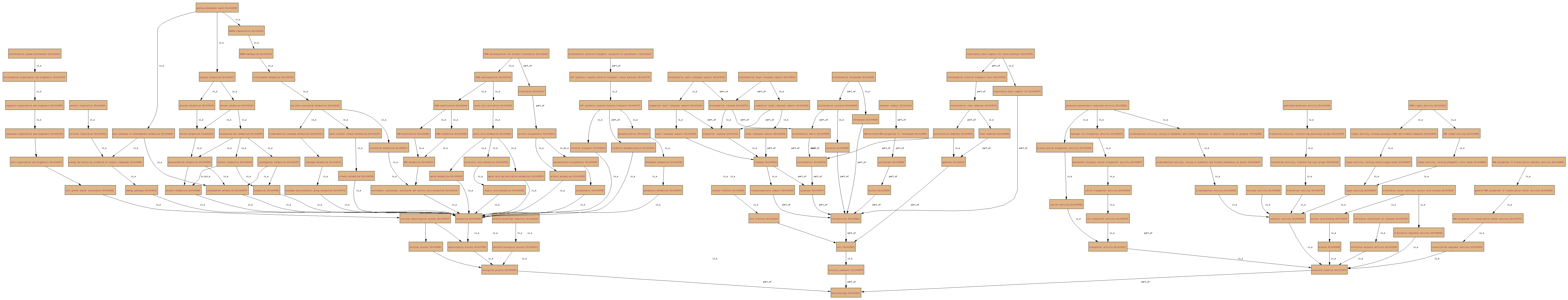

Supplement: Additional File 2 — H2O2 dataset analysed with Amigo DAG View. Figure 7 The figure presents the DAG view of all the reported classes shown in table 7 [see Additional file 7]. These classes had p-value < 0.01 (O.log(p) > 2.0). Figure was obtained from AMIGO server. The obtained figure was considered too complex for manual analysis. [file 1471-2105-6-162-S2.png]

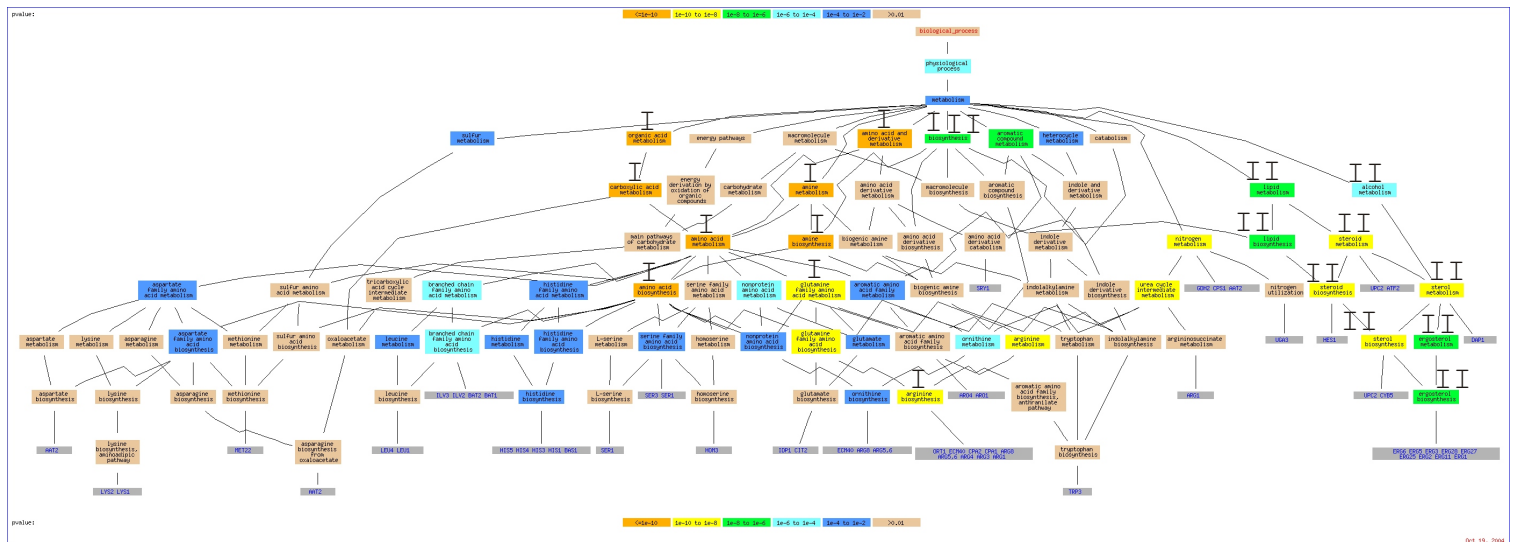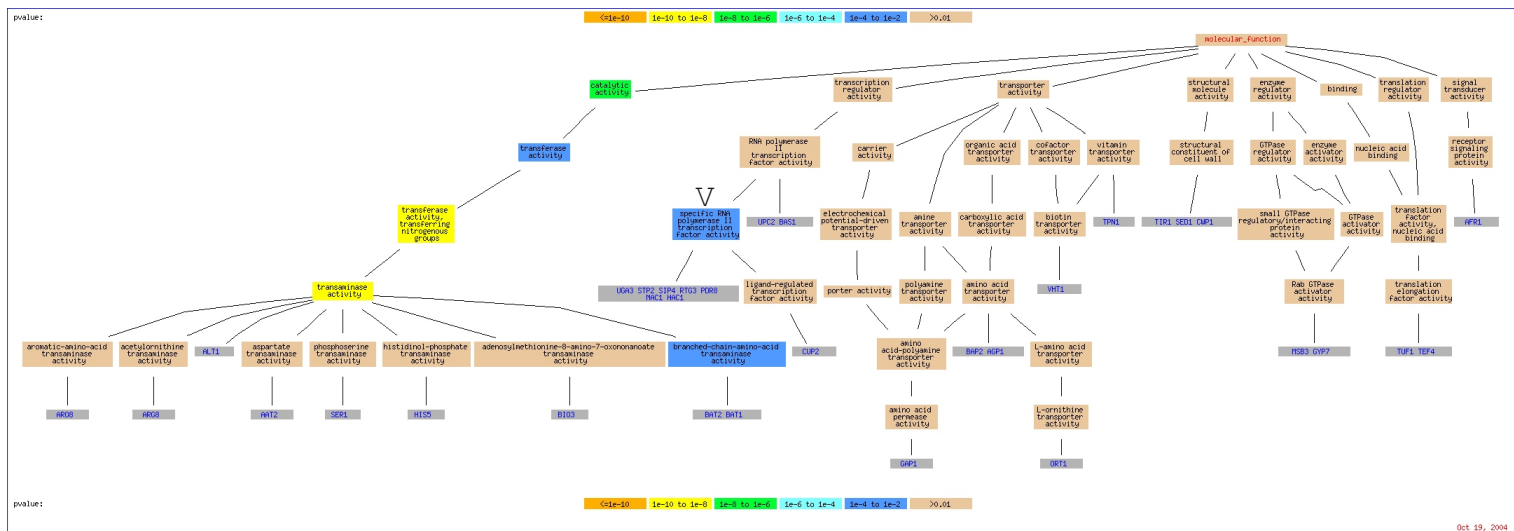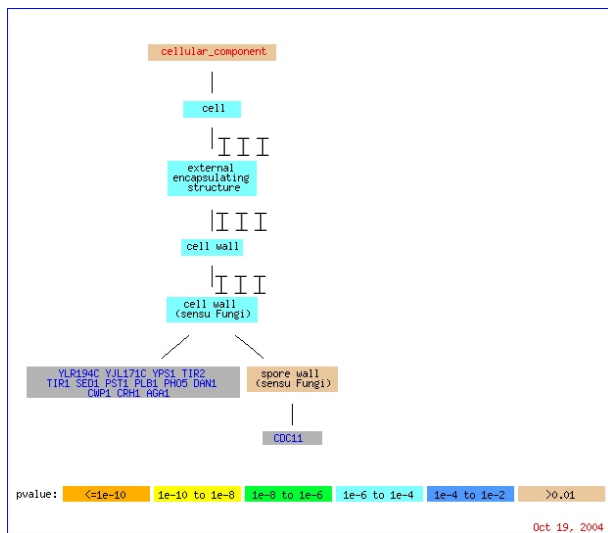

Supplement: Additional File 4 — Itraconanzole data analysed with SGD DAG View. Figure 9 Figure presents the three DAG tree figures obtained from SGD GO term finder using itraconanzole data. The reported classes are colour coded according the reported p-value. We have marked the classes that were reported by some cluster in GENERATOR results by adding the number of corresponding cluster. Note that many classes that were not reported by GENERATOR are usually close in the hierarchy to already reported classes. [file 1471-2105-6-162-S4.pdf]
