# Supplementary material for: Theme discovery from gene lists for identification and viewing of multiple functional groups
Source: BMC Bioinformatics. 2005 Jun 29;6:162. doi: 10.1186/1471-2105-6-162 (PMC1190153; doi:10.1186/1471-2105-6-162)
Supplement: Additional File 10 — GOToolBox outputs from analysis with H2O2 and itraconanzole datasets. Table 10 Files include the clustering results for H2O2 and itraconanzole datasets from GOToolBox. [file 1471-2105-6-162-S10.zip › gotbx-itra-data-MF-default.htm]

GOToolBox


|  |
| --- |
| GO-Proxy : GO-based Gene Clustering |
| Home | Create-Dataset | Store-Ref | GO-Stats | GO-Proxy | GO-Family | Help | |

**The program has found 29 Classes**

MATRIX\_FILE

|  |  |  |  |
| --- | --- | --- | --- |
| Class 1 | size: 6 gene products | | | |
| PCL5 |  SLI15 |  GYP7 |  MSB3 |  GIC2 |  YHR138C | | | |
| GO:0030234 | enzyme regulator activity | 2.302e-11 | E |

  
  

|  |  |  |  |
| --- | --- | --- | --- |
| Class 2 | size: 249 gene products | | | |
| GPI11 |  ERG6 |  ARG3 |  HIS5 |  ALT1 |  BAT2 |  BAT1 |  AAT2 |  ARO8 |  BIO3 |  SER1 |  ARG8 |  STR2 |  RIB5 |  ARO4 |  YGR043C |  ILV2 |  LEU4 |  CIT2 |  MNT4 |  CHS1 |  FUR1 |  HIS1 |  URA10 |  SWE1 |  PRR2 |  HOM3 |  ARG5 |  6 |  POL30 |  PSA1 |  CHO1 |  ELO1 |  SUR4 |  ATF2 |  ECM40 |  LYS1 |  IDP1 |  YPL088W |  AAD16 |  SER3 |  ERG27 |  ADH5 |  UGA2 |  LYS2 |  ERG3 |  SUR2 |  GCV2 |  PDX3 |  GDH2 |  MET16 |  BNA1 |  TSA2 |  ERG25 |  ERG11 |  ERG1 |  YJL200C |  HIS3 |  LEU1 |  ILV3 |  NCE103 |  YFR055W |  ARG4 |  TRP3 |  ARO10 |  SRY1 |  PYC1 |  CPA2 |  CPA1 |  ARG1 |  ULA1 |  ERG5 |  RIB3 |  ERG2 |  ARO1 |  ENA5 |  RSB1 |  AUS1 |  CAF16 |  SGE1 |  YPT53 |  SSB1 |  NIT1 |  FOL2 |  RIB1 |  AAH1 |  ADE17 |  HIS4 |  ADE3 |  YJL113W |  AAP1prime |  YPS3 |  YPS1 |  HSP33 |  CPS1 |  PPT1 |  MET22 |  SDT1 |  PHO3 |  PHO5 |  PHO12 |  PLB1 |  AMS1 |  NTH1 |  YBR147W |  YHR127W |  ICY1 |  YLR152C |  KRR1 |  YJL206C |  YOR385W |  SPT21 |  PRM4 |  SCM4 |  YLR194C |  HUA1 |  YIL056W |  BOP2 |  BSD2 |  STF1 |  RCR1 |  YJR111C |  YPL033C |  BCP1 |  YGR110W |  MCD1 |  YET1 |  YNR068C |  YNL115C |  YDR222W |  TIR2 |  YIL158W |  PET10 |  YIR041W |  PIN3 |  COS111 |  YOR289W |  YJL171C |  DIA1 |  YGL117W |  YJL213W |  FMP23 |  YNL208W |  YKL121W |  ECM1 |  IRS4 |  YER158C |  FMP12 |  YPL282C |  YOR394W |  YJL016W |  ERP3 |  CRH1 |  FYV7 |  FMP43 |  BOP1 |  YER189W |  BNI5 |  DRE2 |  YEH1 |  YKL224C |  YPL272C |  DAP1 |  FAP7 |  ZTA1 |  SNO1 |  GIR2 |  HAS1 |  PDH1 |  YOR338W |  YDR542W |  YHR029C |  DAN1 |  YET2 |  YMR009W |  SRL3 |  HBT1 |  SRO77 |  UBX6 |  PST1 |  BSC5 |  YOR302W |  SIP4 |  RTG3 |  STP2 |  UGA3 |  HAC1 |  MAC1 |  CUP2 |  BAS1 |  UPC2 |  RIM101 |  MSB2 |  AFR1 |  UTP4 |  UTP30 |  IST3 |  MSN2 |  RDS1 |  PIP2 |  TEA1 |  HTA2 |  PDR8 |  CTF13 |  TEF4 |  TUF1 |  TIF35 |  HSP26 |  SSZ1 |  NVJ1 |  ISU1 |  YNL311C |  ERG28 |  AGA1 |  REC8 |  PEX21 |  HES1 |  WTM1 |  TIR1 |  SED1 |  CWP1 |  PDS5 |  SPC42 |  MRPL49 |  CDC11 |  PCL5 |  SLI15 |  GYP7 |  MSB3 |  GIC2 |  YHR138C |  AGP1 |  BAP2 |  ORT1 |  MCH1 |  GAP1 |  TPN1 |  VHT1 |  CYB5 |  OPT1 |  NCR1 |  ATR1 |  GGC1 |  ENB1 |  ZRT1 |  ATP15 |  SSU1 |  PHO89 |  HXT2 | | | |
| GO:0003674 | molecular\_function | 0.000000 | E |

  
  

|  |  |  |  |
| --- | --- | --- | --- |
| Class 3 | size: 7 gene products | | | |
| MSN2 |  RDS1 |  PIP2 |  TEA1 |  HTA2 |  PDR8 |  CTF13 | | | |
| GO:0003677 | DNA binding | 9.514e-14 | E |

  
  

|  |  |  |  |
| --- | --- | --- | --- |
| Class 4 | size: 20 gene products | | | |
| LYS1 |  IDP1 |  YPL088W |  AAD16 |  SER3 |  ERG27 |  ADH5 |  UGA2 |  LYS2 |  ERG3 |  SUR2 |  GCV2 |  PDX3 |  GDH2 |  MET16 |  BNA1 |  TSA2 |  ERG25 |  ERG11 |  ERG1 | | | |
| GO:0016491 | oxidoreductase activity | 7.333e-26 | E |

  
  

|  |  |  |  |
| --- | --- | --- | --- |
| Class 5 | size: 6 gene products | | | |
| YJL200C |  HIS3 |  LEU1 |  ILV3 |  NCE103 |  YFR055W | | | |
| GO:0016836 | hydro-lyase activity | 3.289e-12 | E |
| GO:0016835 | carbon-oxygen lyase activity | 3.289e-12 | E |

  
  

|  |  |  |  |
| --- | --- | --- | --- |
| Class 6 | size: 29 gene products | | | |
| ENA5 |  RSB1 |  AUS1 |  CAF16 |  SGE1 |  YPT53 |  SSB1 |  NIT1 |  FOL2 |  RIB1 |  AAH1 |  ADE17 |  HIS4 |  ADE3 |  YJL113W |  AAP1prime |  YPS3 |  YPS1 |  HSP33 |  CPS1 |  PPT1 |  MET22 |  SDT1 |  PHO3 |  PHO5 |  PHO12 |  PLB1 |  AMS1 |  NTH1 | | | |
| GO:0016787 | hydrolase activity | 1.767e-38 | E |

  
  

|  |  |  |  |
| --- | --- | --- | --- |
| Class 7 | size: 31 gene products | | | |
| UTP4 |  UTP30 |  IST3 |  MSN2 |  RDS1 |  PIP2 |  TEA1 |  HTA2 |  PDR8 |  CTF13 |  TEF4 |  TUF1 |  TIF35 |  HSP26 |  SSZ1 |  NVJ1 |  ISU1 |  YNL311C |  ERG28 |  AGA1 |  REC8 |  PEX21 |  HES1 |  WTM1 |  TIR1 |  SED1 |  CWP1 |  PDS5 |  SPC42 |  MRPL49 |  CDC11 | | | |
| GO:0005488 | binding | 0.000000 | E |

  
  

|  |  |  |  |
| --- | --- | --- | --- |
| Class 8 | size: 5 gene products | | | |
| AGP1 |  BAP2 |  ORT1 |  MCH1 |  GAP1 | | | |
| GO:0005275 | amine transporter activity | 0.000000 | E |
| GO:0015171 | amino acid transporter activity | 0.000000 | E |
| GO:0005342 | organic acid transporter activity | 1.332e-10 | E |
| GO:0046943 | carboxylic acid transporter activity | 1.332e-10 | E |

  
  

|  |  |  |  |
| --- | --- | --- | --- |
| Class 9 | size: 10 gene products | | | |
| SIP4 |  RTG3 |  STP2 |  UGA3 |  HAC1 |  MAC1 |  CUP2 |  BAS1 |  UPC2 |  RIM101 | | | |
| GO:0003702 | RNA polymerase II transcription factor activity | 4.955e-18 | E |
| GO:0030528 | transcription regulator activity | 1.488e-14 | E |

  
  

|  |  |  |  |
| --- | --- | --- | --- |
| Class 10 | size: 90 gene products | | | |
| YBR147W |  YHR127W |  ICY1 |  YLR152C |  KRR1 |  YJL206C |  YOR385W |  SPT21 |  PRM4 |  SCM4 |  YLR194C |  HUA1 |  YIL056W |  BOP2 |  BSD2 |  STF1 |  RCR1 |  YJR111C |  YPL033C |  BCP1 |  YGR110W |  MCD1 |  YET1 |  YNR068C |  YNL115C |  YDR222W |  TIR2 |  YIL158W |  PET10 |  YIR041W |  PIN3 |  COS111 |  YOR289W |  YJL171C |  DIA1 |  YGL117W |  YJL213W |  FMP23 |  YNL208W |  YKL121W |  ECM1 |  IRS4 |  YER158C |  FMP12 |  YPL282C |  YOR394W |  YJL016W |  ERP3 |  CRH1 |  FYV7 |  FMP43 |  BOP1 |  YER189W |  BNI5 |  DRE2 |  YEH1 |  YKL224C |  YPL272C |  DAP1 |  FAP7 |  ZTA1 |  SNO1 |  GIR2 |  HAS1 |  PDH1 |  YOR338W |  YDR542W |  YHR029C |  DAN1 |  YET2 |  YMR009W |  SRL3 |  HBT1 |  SRO77 |  UBX6 |  PST1 |  BSC5 |  YOR302W |  SIP4 |  RTG3 |  STP2 |  UGA3 |  HAC1 |  MAC1 |  CUP2 |  BAS1 |  UPC2 |  RIM101 |  MSB2 |  AFR1 | | | |
| GO:0005554 | molecular\_function unknown | 0.000000 | E |

  
  

|  |  |  |  |
| --- | --- | --- | --- |
| Class 11 | size: 7 gene products | | | |
| TIR1 |  SED1 |  CWP1 |  PDS5 |  SPC42 |  MRPL49 |  CDC11 | | | |
| GO:0005198 | structural molecule activity | 9.514e-14 | E |

  
  

|  |  |  |  |
| --- | --- | --- | --- |
| Class 12 | size: 7 gene products | | | |
| PPT1 |  MET22 |  SDT1 |  PHO3 |  PHO5 |  PHO12 |  PLB1 | | | |
| GO:0016791 | phosphoric monoester hydrolase activity | 0.000000 | E |
| GO:0042578 | phosphoric ester hydrolase activity | 0.000000 | E |
| GO:0016788 | hydrolase activity, acting on ester bonds | 7.611e-13 | E |

  
  

|  |  |  |  |
| --- | --- | --- | --- |
| Class 13 | size: 10 gene products | | | |
| YJL200C |  HIS3 |  LEU1 |  ILV3 |  NCE103 |  YFR055W |  ARG4 |  TRP3 |  ARO10 |  SRY1 | | | |
| GO:0016829 | lyase activity | 5.451e-17 | E |

  
  

|  |  |  |  |
| --- | --- | --- | --- |
| Class 14 | size: 5 gene products | | | |
| ENA5 |  RSB1 |  AUS1 |  CAF16 |  SGE1 | | | |
| GO:0042623 | ATPase activity, coupled | 1.332e-10 | E |
| GO:0042626 | ATPase activity, coupled to transmembrane movement of substances | 1.332e-10 | E |
| GO:0016820 | hydrolase activity, acting on acid anhydrides, catalyzing transmembrane movement of substances | 1.332e-10 | E |

  
  

|  |  |  |  |
| --- | --- | --- | --- |
| Class 15 | size: 5 gene products | | | |
| AAP1prime |  YPS3 |  YPS1 |  HSP33 |  CPS1 | | | |
| GO:0008233 | peptidase activity | 7.993e-10 | E |

  
  

|  |  |  |  |
| --- | --- | --- | --- |
| Class 16 | size: 7 gene products | | | |
| NIT1 |  FOL2 |  RIB1 |  AAH1 |  ADE17 |  HIS4 |  ADE3 | | | |
| GO:0016814 | hydrolase activity, acting on carbon-nitrogen (but not peptide) bonds, in cyclic amidines | 0.000000 | E |
| GO:0016810 | hydrolase activity, acting on carbon-nitrogen (but not peptide) bonds | 9.514e-14 | E |

  
  

|  |  |  |  |
| --- | --- | --- | --- |
| Class 17 | size: 7 gene products | | | |
| ENA5 |  RSB1 |  AUS1 |  CAF16 |  SGE1 |  YPT53 |  SSB1 | | | |
| GO:0016887 | ATPase activity | 0.000000 | E |
| GO:0017111 | nucleoside-triphosphatase activity | 9.514e-14 | E |
| GO:0016818 | hydrolase activity, acting on acid anhydrides, in phosphorus-containing anhydrides | 7.611e-13 | E |
| GO:0016817 | hydrolase activity, acting on acid anhydrides | 7.611e-13 | E |
| GO:0016462 | pyrophosphatase activity | 7.611e-13 | E |

  
  

|  |  |  |  |
| --- | --- | --- | --- |
| Class 18 | size: 5 gene products | | | |
| MNT4 |  CHS1 |  FUR1 |  HIS1 |  URA10 | | | |
| GO:0016757 | transferase activity, transferring glycosyl groups | 1.332e-10 | E |

  
  

|  |  |  |  |
| --- | --- | --- | --- |
| Class 19 | size: 13 gene products | | | |
| UTP4 |  UTP30 |  IST3 |  MSN2 |  RDS1 |  PIP2 |  TEA1 |  HTA2 |  PDR8 |  CTF13 |  TEF4 |  TUF1 |  TIF35 | | | |
| GO:0003676 | nucleic acid binding | 8.943e-21 | E |

  
  

|  |  |  |  |
| --- | --- | --- | --- |
| Class 20 | size: 5 gene products | | | |
| ENB1 |  ZRT1 |  ATP15 |  SSU1 |  PHO89 | | | |
| GO:0008324 | cation transporter activity | 7.993e-10 | E |
| GO:0015075 | ion transporter activity | 2.797e-09 | E |

  
  

|  |  |  |  |
| --- | --- | --- | --- |
| Class 21 | size: 18 gene products | | | |
| AGP1 |  BAP2 |  ORT1 |  MCH1 |  GAP1 |  TPN1 |  VHT1 |  CYB5 |  OPT1 |  NCR1 |  ATR1 |  GGC1 |  ENB1 |  ZRT1 |  ATP15 |  SSU1 |  PHO89 |  HXT2 | | | |
| GO:0005215 | transporter activity | 1.272e-24 | E |

  
  

|  |  |  |  |
| --- | --- | --- | --- |
| Class 22 | size: 104 gene products | | | |
| GPI11 |  ERG6 |  ARG3 |  HIS5 |  ALT1 |  BAT2 |  BAT1 |  AAT2 |  ARO8 |  BIO3 |  SER1 |  ARG8 |  STR2 |  RIB5 |  ARO4 |  YGR043C |  ILV2 |  LEU4 |  CIT2 |  MNT4 |  CHS1 |  FUR1 |  HIS1 |  URA10 |  SWE1 |  PRR2 |  HOM3 |  ARG5 |  6 |  POL30 |  PSA1 |  CHO1 |  ELO1 |  SUR4 |  ATF2 |  ECM40 |  LYS1 |  IDP1 |  YPL088W |  AAD16 |  SER3 |  ERG27 |  ADH5 |  UGA2 |  LYS2 |  ERG3 |  SUR2 |  GCV2 |  PDX3 |  GDH2 |  MET16 |  BNA1 |  TSA2 |  ERG25 |  ERG11 |  ERG1 |  YJL200C |  HIS3 |  LEU1 |  ILV3 |  NCE103 |  YFR055W |  ARG4 |  TRP3 |  ARO10 |  SRY1 |  PYC1 |  CPA2 |  CPA1 |  ARG1 |  ULA1 |  ERG5 |  RIB3 |  ERG2 |  ARO1 |  ENA5 |  RSB1 |  AUS1 |  CAF16 |  SGE1 |  YPT53 |  SSB1 |  NIT1 |  FOL2 |  RIB1 |  AAH1 |  ADE17 |  HIS4 |  ADE3 |  YJL113W |  AAP1prime |  YPS3 |  YPS1 |  HSP33 |  CPS1 |  PPT1 |  MET22 |  SDT1 |  PHO3 |  PHO5 |  PHO12 |  PLB1 |  AMS1 |  NTH1 | | | |
| GO:0003824 | catalytic activity | 0.000000 | E |

  
  

|  |  |  |  |
| --- | --- | --- | --- |
| Class 23 | size: 4 gene products | | | |
| ELO1 |  SUR4 |  ATF2 |  ECM40 | | | |
| GO:0008415 | acyltransferase activity | 6.501e-09 | E |
| GO:0016747 | transferase activity, transferring groups other than amino-acyl groups | 6.501e-09 | E |
| GO:0016746 | transferase activity, transferring acyl groups | 9.751e-08 | E |

  
  

|  |  |  |  |
| --- | --- | --- | --- |
| Class 24 | size: 6 gene products | | | |
| IDP1 |  YPL088W |  AAD16 |  SER3 |  ERG27 |  ADH5 | | | |
| GO:0016614 | oxidoreductase activity, acting on CH-OH group of donors | 9.210e-11 | E |
| GO:0016616 | oxidoreductase activity, acting on the CH-OH group of donors, NAD or NADP as acceptor | 9.210e-11 | E |

  
  

|  |  |  |  |
| --- | --- | --- | --- |
| Class 25 | size: 4 gene products | | | |
| PYC1 |  CPA2 |  CPA1 |  ARG1 | | | |
| GO:0016874 | ligase activity | 3.250e-08 | E |

  
  

|  |  |  |  |
| --- | --- | --- | --- |
| Class 26 | size: 9 gene products | | | |
| HIS5 |  ALT1 |  BAT2 |  BAT1 |  AAT2 |  ARO8 |  BIO3 |  SER1 |  ARG8 | | | |
| GO:0008483 | transaminase activity | 1.184e-16 | E |
| GO:0016769 | transferase activity, transferring nitrogenous groups | 1.184e-16 | E |

  
  

|  |  |  |  |
| --- | --- | --- | --- |
| Class 27 | size: 7 gene products | | | |
| SIP4 |  RTG3 |  STP2 |  UGA3 |  HAC1 |  MAC1 |  CUP2 | | | |
| GO:0003704 | specific RNA polymerase II transcription factor activity | 7.611e-13 | E |

  
  

|  |  |  |  |
| --- | --- | --- | --- |
| Class 28 | size: 36 gene products | | | |
| GPI11 |  ERG6 |  ARG3 |  HIS5 |  ALT1 |  BAT2 |  BAT1 |  AAT2 |  ARO8 |  BIO3 |  SER1 |  ARG8 |  STR2 |  RIB5 |  ARO4 |  YGR043C |  ILV2 |  LEU4 |  CIT2 |  MNT4 |  CHS1 |  FUR1 |  HIS1 |  URA10 |  SWE1 |  PRR2 |  HOM3 |  ARG5 |  6 |  POL30 |  PSA1 |  CHO1 |  ELO1 |  SUR4 |  ATF2 |  ECM40 | | | |
| GO:0016740 | transferase activity | 2.384e-41 | E |

  
  

|  |  |  |  |
| --- | --- | --- | --- |
| Class 29 | size: 11 gene products | | | |
| HSP26 |  SSZ1 |  NVJ1 |  ISU1 |  YNL311C |  ERG28 |  AGA1 |  REC8 |  PEX21 |  HES1 |  WTM1 | | | |
| GO:0005515 | protein binding | 2.748e-18 | E |

  
  
