# Supplementary material for: Theme discovery from gene lists for identification and viewing of multiple functional groups
Source: BMC Bioinformatics. 2005 Jun 29;6:162. doi: 10.1186/1471-2105-6-162 (PMC1190153; doi:10.1186/1471-2105-6-162)
Supplement: Additional File 10 — GOToolBox outputs from analysis with H2O2 and itraconanzole datasets. Table 10 Files include the clustering results for H2O2 and itraconanzole datasets from GOToolBox. [file 1471-2105-6-162-S10.zip › gotbx-H2O2-data--CL-default.htm]

GOToolBox


|  |
| --- |
| GO-Proxy : GO-based Gene Clustering |
| Home | Create-Dataset | Store-Ref | GO-Stats | GO-Proxy | GO-Family | Help | |

**The program has found 22 Classes**

MATRIX\_FILE

|  |  |  |  |
| --- | --- | --- | --- |
| Class 1 | size: 62 gene products | | | |
| GAL11 |  SRB5 |  ROX3 |  SIN4 |  PAF1 |  SNF2 |  HFI1 |  AEP1 |  MEF2 |  MSE1 |  MTF2 |  YOR305W |  ISM1 |  GND1 |  DEM1 |  PET112 |  SOV1 |  PCP1 |  YGR102C |  YPR116W |  NAM2 |  MSM1 |  DIA4 |  IDP1 |  COQ1 |  YDR332W |  MSF1 |  YGR150C |  FMP53 |  RIM1 |  MEF1 |  MTO2 |  PIF1 |  MSS116 |  ATP12 |  HMI1 |  SUV3 |  MTF1 |  SSQ1 |  VPS75 |  SKN7 |  MBP1 |  STB5 |  RTT109 |  YAP1 |  ARV1 |  EUG1 |  CCC1 |  FAB1 |  GGC1 |  CBS1 |  COQ6 |  CBS2 |  PET309 |  AEP3 |  CBP3 |  SLS1 |  CYT1 |  QCR7 |  QCR8 |  COR1 |  ATP5 | | | |
| GO:0043231 | intracellular membrane-bound organelle | 2.739e-08 | E |
| GO:0043227 | membrane-bound organelle | 2.739e-08 | E |

  
  

|  |  |  |  |
| --- | --- | --- | --- |
| Class 2 | size: 32 gene products | | | |
| AEP1 |  MEF2 |  MSE1 |  MTF2 |  YOR305W |  ISM1 |  GND1 |  DEM1 |  PET112 |  SOV1 |  PCP1 |  YGR102C |  YPR116W |  NAM2 |  MSM1 |  DIA4 |  IDP1 |  COQ1 |  YDR332W |  MSF1 |  YGR150C |  FMP53 |  RIM1 |  MEF1 |  MTO2 |  PIF1 |  MSS116 |  ATP12 |  HMI1 |  SUV3 |  MTF1 |  SSQ1 | | | |
| GO:0005739 | mitochondrion | 4.129e-07 | E |
| GO:0005737 | cytoplasm | 0.000167 | E |

  
  

|  |  |  |  |
| --- | --- | --- | --- |
| Class 3 | size: 29 gene products | | | |
| MRPL35 |  YPL183W-A |  MRPL20 |  MRPL25 |  MRPL22 |  MRPL16 |  MRPL17 |  MRPL37 |  MRPL24 |  MRPL13 |  MRPL6 |  MRPL28 |  MRPL11 |  MRPL51 |  MRPL40 |  IMG1 |  MRPL7 |  MRPL9 |  MRPS16 |  MRPS8 |  YNR036C |  RSM19 |  MRP2 |  MRPS35 |  MRP10 |  RSM24 |  RSM25 |  MGM101 |  ABF2 | | | |
| GO:0005759 | mitochondrial matrix | 3.659e-20 | E |
| GO:0005739 | mitochondrion | 2.235e-06 | E |
| GO:0043231 | intracellular membrane-bound organelle | 0.002130 | E |
| GO:0043227 | membrane-bound organelle | 0.002130 | E |

  
  

|  |  |  |  |
| --- | --- | --- | --- |
| Class 4 | size: 33 gene products | | | |
| MRPL35 |  YPL183W-A |  MRPL20 |  MRPL25 |  MRPL22 |  MRPL16 |  MRPL17 |  MRPL37 |  MRPL24 |  MRPL13 |  MRPL6 |  MRPL28 |  MRPL11 |  MRPL51 |  MRPL40 |  IMG1 |  MRPL7 |  MRPL9 |  MRPS16 |  MRPS8 |  YNR036C |  RSM19 |  MRP2 |  MRPS35 |  MRP10 |  RSM24 |  RSM25 |  MGM101 |  ABF2 |  YGR054W |  RPL21A |  SLA1 |  DBF2 | | | |
| GO:0043228 | non-membrane-bound organelle | 1.134e-28 | E |
| GO:0043232 | intracellular non-membrane-bound organelle | 1.134e-28 | E |

  
  

|  |  |  |  |
| --- | --- | --- | --- |
| Class 5 | size: 4 gene products | | | |
| RPE1 |  TKL1 |  TVP38 |  GLO3 | | | |
| GO:0005737 | cytoplasm | 0.400107 | E |

  
  

|  |  |  |  |
| --- | --- | --- | --- |
| Class 6 | size: 7 gene products | | | |
| GAL11 |  SRB5 |  ROX3 |  SIN4 |  PAF1 |  SNF2 |  HFI1 | | | |
| GO:0005654 | nucleoplasm | 3.356e-11 | E |
| GO:0005634 | nucleus | 1.152e-07 | E |
| GO:0043234 | protein complex | 0.000905 | E |

  
  

|  |  |  |  |
| --- | --- | --- | --- |
| Class 7 | size: 18 gene products | | | |
| MRPL35 |  YPL183W-A |  MRPL20 |  MRPL25 |  MRPL22 |  MRPL16 |  MRPL17 |  MRPL37 |  MRPL24 |  MRPL13 |  MRPL6 |  MRPL28 |  MRPL11 |  MRPL51 |  MRPL40 |  IMG1 |  MRPL7 |  MRPL9 | | | |
| GO:0005762 | mitochondrial large ribosomal subunit | 5.995e-21 | E |
| GO:0000315 | organellar large ribosomal subunit | 5.995e-21 | E |
| GO:0015934 | large ribosomal subunit | 1.139e-19 | E |

  
  

|  |  |  |  |
| --- | --- | --- | --- |
| Class 8 | size: 6 gene products | | | |
| RPE1 |  TKL1 |  TVP38 |  GLO3 |  GSH1 |  REG1 | | | |
| GO:0005622 | intracellular | 0.625840 | E |

  
  

|  |  |  |  |
| --- | --- | --- | --- |
| Class 9 | size: 4 gene products | | | |
| CYT1 |  QCR7 |  QCR8 |  COR1 | | | |
| GO:0045275 | respiratory chain complex III | 1.798e-07 | E |
| GO:0005746 | mitochondrial electron transport chain | 1.798e-07 | E |
| GO:0005750 | respiratory chain complex III (sensu Eukaryota) | 1.798e-07 | E |
| GO:0005743 | mitochondrial inner membrane | 8.898e-05 | E |
| GO:0019866 | inner membrane | 8.898e-05 | E |
| GO:0043234 | protein complex | 0.020120 | E |

  
  

|  |  |  |  |
| --- | --- | --- | --- |
| Class 10 | size: 9 gene products | | | |
| MRPS16 |  MRPS8 |  YNR036C |  RSM19 |  MRP2 |  MRPS35 |  MRP10 |  RSM24 |  RSM25 | | | |
| GO:0005763 | mitochondrial small ribosomal subunit | 2.346e-13 | E |
| GO:0000314 | organellar small ribosomal subunit | 2.346e-13 | E |
| GO:0015935 | small ribosomal subunit | 2.346e-12 | E |

  
  

|  |  |  |  |
| --- | --- | --- | --- |
| Class 11 | size: 6 gene products | | | |
| VPS75 |  SKN7 |  MBP1 |  STB5 |  RTT109 |  YAP1 | | | |
| GO:0005634 | nucleus | 1.483e-06 | E |

  
  

|  |  |  |  |
| --- | --- | --- | --- |
| Class 12 | size: 4 gene products | | | |
| GAL11 |  SRB5 |  ROX3 |  SIN4 | | | |
| GO:0000119 | mediator complex | 1.798e-07 | E |

  
  

|  |  |  |  |
| --- | --- | --- | --- |
| Class 13 | size: 31 gene products | | | |
| MRPL35 |  YPL183W-A |  MRPL20 |  MRPL25 |  MRPL22 |  MRPL16 |  MRPL17 |  MRPL37 |  MRPL24 |  MRPL13 |  MRPL6 |  MRPL28 |  MRPL11 |  MRPL51 |  MRPL40 |  IMG1 |  MRPL7 |  MRPL9 |  MRPS16 |  MRPS8 |  YNR036C |  RSM19 |  MRP2 |  MRPS35 |  MRP10 |  RSM24 |  RSM25 |  MGM101 |  ABF2 |  YGR054W |  RPL21A | | | |
| GO:0005737 | cytoplasm | 0.000233 | E |

  
  

|  |  |  |  |
| --- | --- | --- | --- |
| Class 14 | size: 27 gene products | | | |
| MRPL35 |  YPL183W-A |  MRPL20 |  MRPL25 |  MRPL22 |  MRPL16 |  MRPL17 |  MRPL37 |  MRPL24 |  MRPL13 |  MRPL6 |  MRPL28 |  MRPL11 |  MRPL51 |  MRPL40 |  IMG1 |  MRPL7 |  MRPL9 |  MRPS16 |  MRPS8 |  YNR036C |  RSM19 |  MRP2 |  MRPS35 |  MRP10 |  RSM24 |  RSM25 | | | |
| GO:0005761 | mitochondrial ribosome | 3.585e-26 | E |
| GO:0000313 | organellar ribosome | 3.585e-26 | E |
| GO:0005840 | ribosome | 1.455e-23 | E |
| GO:0030529 | ribonucleoprotein complex | 1.455e-23 | E |
| GO:0043234 | protein complex | 3.537e-15 | E |

  
  

|  |  |  |  |
| --- | --- | --- | --- |
| Class 15 | size: 109 gene products | | | |
| MRPL35 |  YPL183W-A |  MRPL20 |  MRPL25 |  MRPL22 |  MRPL16 |  MRPL17 |  MRPL37 |  MRPL24 |  MRPL13 |  MRPL6 |  MRPL28 |  MRPL11 |  MRPL51 |  MRPL40 |  IMG1 |  MRPL7 |  MRPL9 |  MRPS16 |  MRPS8 |  YNR036C |  RSM19 |  MRP2 |  MRPS35 |  MRP10 |  RSM24 |  RSM25 |  MGM101 |  ABF2 |  YGR054W |  RPL21A |  SLA1 |  DBF2 |  GAL11 |  SRB5 |  ROX3 |  SIN4 |  PAF1 |  SNF2 |  HFI1 |  AEP1 |  MEF2 |  MSE1 |  MTF2 |  YOR305W |  ISM1 |  GND1 |  DEM1 |  PET112 |  SOV1 |  PCP1 |  YGR102C |  YPR116W |  NAM2 |  MSM1 |  DIA4 |  IDP1 |  COQ1 |  YDR332W |  MSF1 |  YGR150C |  FMP53 |  RIM1 |  MEF1 |  MTO2 |  PIF1 |  MSS116 |  ATP12 |  HMI1 |  SUV3 |  MTF1 |  SSQ1 |  VPS75 |  SKN7 |  MBP1 |  STB5 |  RTT109 |  YAP1 |  ARV1 |  EUG1 |  CCC1 |  FAB1 |  GGC1 |  CBS1 |  COQ6 |  CBS2 |  PET309 |  AEP3 |  CBP3 |  SLS1 |  CYT1 |  QCR7 |  QCR8 |  COR1 |  ATP5 |  RPE1 |  TKL1 |  TVP38 |  GLO3 |  GSH1 |  REG1 |  YNL080C |  BAP2 |  HSP31 |  URE2 |  MCK1 |  LCB5 |  YML036W |  YLR149C | | | |
| GO:0005575 | cellular\_component | 1.000000 | E |

  
  

|  |  |  |  |
| --- | --- | --- | --- |
| Class 16 | size: 13 gene products | | | |
| GGC1 |  CBS1 |  COQ6 |  CBS2 |  PET309 |  AEP3 |  CBP3 |  SLS1 |  CYT1 |  QCR7 |  QCR8 |  COR1 |  ATP5 | | | |
| GO:0005740 | mitochondrial membrane | 4.277e-17 | E |
| GO:0016020 | membrane | 1.018e-13 | E |
| GO:0005739 | mitochondrion | 0.005415 | E |
| GO:0005737 | cytoplasm | 0.043764 | E |

  
  

|  |  |  |  |
| --- | --- | --- | --- |
| Class 17 | size: 5 gene products | | | |
| GAL11 |  SRB5 |  ROX3 |  SIN4 |  PAF1 | | | |
| GO:0016591 | DNA-directed RNA polymerase II, holoenzyme | 8.560e-09 | E |

  
  

|  |  |  |  |
| --- | --- | --- | --- |
| Class 18 | size: 6 gene products | | | |
| GGC1 |  CBS1 |  COQ6 |  CBS2 |  PET309 |  AEP3 | | | |
| GO:0005743 | mitochondrial inner membrane | 4.563e-07 | E |
| GO:0019866 | inner membrane | 4.563e-07 | E |

  
  

|  |  |  |  |
| --- | --- | --- | --- |
| Class 19 | size: 12 gene products | | | |
| RPE1 |  TKL1 |  TVP38 |  GLO3 |  GSH1 |  REG1 |  YNL080C |  BAP2 |  HSP31 |  URE2 |  MCK1 |  LCB5 | | | |
| GO:0005623 | cell | 0.791030 | E |

  
  

|  |  |  |  |
| --- | --- | --- | --- |
| Class 20 | size: 6 gene products | | | |
| MSS116 |  ATP12 |  HMI1 |  SUV3 |  MTF1 |  SSQ1 | | | |
| GO:0005759 | mitochondrial matrix | 0.000962 | E |

  
  

|  |  |  |  |
| --- | --- | --- | --- |
| Class 21 | size: 4 gene products | | | |
| HSP31 |  URE2 |  MCK1 |  LCB5 | | | |
| GO:0005625 | soluble fraction | 1.798e-07 | E |
| GO:0000267 | cell fraction | 8.988e-07 | E |

  
  

|  |  |  |  |
| --- | --- | --- | --- |
| Class 22 | size: 95 gene products | | | |
| MRPL35 |  YPL183W-A |  MRPL20 |  MRPL25 |  MRPL22 |  MRPL16 |  MRPL17 |  MRPL37 |  MRPL24 |  MRPL13 |  MRPL6 |  MRPL28 |  MRPL11 |  MRPL51 |  MRPL40 |  IMG1 |  MRPL7 |  MRPL9 |  MRPS16 |  MRPS8 |  YNR036C |  RSM19 |  MRP2 |  MRPS35 |  MRP10 |  RSM24 |  RSM25 |  MGM101 |  ABF2 |  YGR054W |  RPL21A |  SLA1 |  DBF2 |  GAL11 |  SRB5 |  ROX3 |  SIN4 |  PAF1 |  SNF2 |  HFI1 |  AEP1 |  MEF2 |  MSE1 |  MTF2 |  YOR305W |  ISM1 |  GND1 |  DEM1 |  PET112 |  SOV1 |  PCP1 |  YGR102C |  YPR116W |  NAM2 |  MSM1 |  DIA4 |  IDP1 |  COQ1 |  YDR332W |  MSF1 |  YGR150C |  FMP53 |  RIM1 |  MEF1 |  MTO2 |  PIF1 |  MSS116 |  ATP12 |  HMI1 |  SUV3 |  MTF1 |  SSQ1 |  VPS75 |  SKN7 |  MBP1 |  STB5 |  RTT109 |  YAP1 |  ARV1 |  EUG1 |  CCC1 |  FAB1 |  GGC1 |  CBS1 |  COQ6 |  CBS2 |  PET309 |  AEP3 |  CBP3 |  SLS1 |  CYT1 |  QCR7 |  QCR8 |  COR1 |  ATP5 | | | |
| GO:0043226 | organelle | 6.237e-18 | E |
| GO:0043229 | intracellular organelle | 6.237e-18 | E |
| GO:0005622 | intracellular | 7.905e-09 | E |
| GO:0005623 | cell | 0.015460 | E |

  
  
