# Supplementary material for: Theme discovery from gene lists for identification and viewing of multiple functional groups
Source: BMC Bioinformatics. 2005 Jun 29;6:162. doi: 10.1186/1471-2105-6-162 (PMC1190153; doi:10.1186/1471-2105-6-162)
Supplement: Additional File 10 — GOToolBox outputs from analysis with H2O2 and itraconanzole datasets. Table 10 Files include the clustering results for H2O2 and itraconanzole datasets from GOToolBox. [file 1471-2105-6-162-S10.zip › gotbx-H2O2-data-default.htm]

GOToolBox


|  |
| --- |
| GO-Proxy : GO-based Gene Clustering |
| Home | Create-Dataset | Store-Ref | GO-Stats | GO-Proxy | GO-Family | Help | |

**The program has found 23 Classes**

MATRIX\_FILE

|  |  |  |  |
| --- | --- | --- | --- |
| Class 1 | size: 8 gene products | | | |
| RIM1 |  HMI1 |  ABF2 |  MGM101 |  FAB1 |  HFI1 |  SNF2 |  PIF1 | | | |
| GO:0016043 | cell organization and biogenesis | 7.905e-09 | E |
| GO:0006996 | organelle organization and biogenesis | 7.905e-09 | E |

  
  

|  |  |  |  |
| --- | --- | --- | --- |
| Class 2 | size: 5 gene products | | | |
| GAL11 |  SIN4 |  SRB5 |  ROX3 |  PAF1 | | | |
| GO:0006366 | transcription from Pol II promoter | 1.798e-07 | E |

  
  

|  |  |  |  |
| --- | --- | --- | --- |
| Class 3 | size: 3 gene products | | | |
| ATP12 |  CBP3 |  EUG1 | | | |
| GO:0019538 | protein metabolism | 0.099198 | E |
| GO:0043170 | macromolecule metabolism | 0.139377 | E |

  
  

|  |  |  |  |
| --- | --- | --- | --- |
| Class 4 | size: 45 gene products | | | |
| MTF2 |  CBS1 |  MSF1 |  MRPL16 |  MRPS16 |  MRPL37 |  IMG1 |  MRPL35 |  MRPL25 |  MRP2 |  MRPL28 |  YPL183W-A |  AEP1 |  MRPL11 |  YNR036C |  RPL21A |  MRPL24 |  RSM19 |  MRPL13 |  RSM25 |  MRP10 |  CBS2 |  MRPL17 |  MRPS8 |  MRPL7 |  MRPL40 |  MRPL6 |  YGR054W |  MEF2 |  MEF1 |  MRPL51 |  SLS1 |  MRPS35 |  RSM24 |  MRPL22 |  PET309 |  MRPL9 |  PET112 |  MRPL20 |  MSE1 |  MSM1 |  ISM1 |  NAM2 |  DIA4 |  IDP1 | | | |
| GO:0009058 | biosynthesis | 1.136e-28 | E |
| GO:0043170 | macromolecule metabolism | 7.435e-20 | E |

  
  

|  |  |  |  |
| --- | --- | --- | --- |
| Class 5 | size: 14 gene products | | | |
| YGR102C |  YML036W |  SOV1 |  YNL080C |  YOR305W |  HSP31 |  TVP38 |  YPR116W |  YDR332W |  DEM1 |  YGR150C |  FMP53 |  YLR149C |  MTO2 | | | |
| GO:0000004 | biological\_process unknown | 6.237e-18 | E |

  
  

|  |  |  |  |
| --- | --- | --- | --- |
| Class 6 | size: 5 gene products | | | |
| MSE1 |  MSM1 |  ISM1 |  NAM2 |  DIA4 | | | |
| GO:0006418 | tRNA aminoacylation for protein translation | 8.560e-09 | E |
| GO:0006400 | tRNA modification | 8.560e-09 | E |
| GO:0043039 | tRNA aminoacylation | 8.560e-09 | E |
| GO:0009451 | RNA modification | 8.560e-09 | E |
| GO:0006399 | tRNA metabolism | 8.560e-09 | E |
| GO:0043038 | amino acid activation | 8.560e-09 | E |
| GO:0006082 | organic acid metabolism | 5.136e-08 | E |
| GO:0009308 | amine metabolism | 5.136e-08 | E |
| GO:0006520 | amino acid metabolism | 5.136e-08 | E |
| GO:0006519 | amino acid and derivative metabolism | 5.136e-08 | E |
| GO:0019752 | carboxylic acid metabolism | 5.136e-08 | E |
| GO:0043037 | translation | 4.793e-07 | E |
| GO:0016070 | RNA metabolism | 2.157e-06 | E |
| GO:0006139 | nucleobase, nucleoside, nucleotide and nucleic acid metabolism | 0.001220 | E |

  
  

|  |  |  |  |
| --- | --- | --- | --- |
| Class 7 | size: 3 gene products | | | |
| MRPL9 |  PET112 |  MRPL20 | | | |
| GO:0006996 | organelle organization and biogenesis | 0.001734 | E |
| GO:0016043 | cell organization and biogenesis | 0.001734 | E |
| GO:0008151 | cell growth and/or maintenance | 0.010956 | E |
| GO:0050875 | cellular physiological process | 0.010956 | E |
| GO:0009987 | cellular process | 0.012385 | E |

  
  

|  |  |  |  |
| --- | --- | --- | --- |
| Class 8 | size: 3 gene products | | | |
| GND1 |  TKL1 |  RPE1 | | | |
| GO:0006006 | glucose metabolism | 4.763e-06 | E |
| GO:0019318 | hexose metabolism | 4.763e-06 | E |
| GO:0005996 | monosaccharide metabolism | 4.763e-06 | E |
| GO:0006066 | alcohol metabolism | 1.905e-05 | E |
| GO:0005975 | carbohydrate metabolism | 4.763e-05 | E |
| GO:0043170 | macromolecule metabolism | 0.139377 | E |

  
  

|  |  |  |  |
| --- | --- | --- | --- |
| Class 9 | size: 44 gene products | | | |
| MTF2 |  CBS1 |  MSF1 |  MRPL16 |  MRPS16 |  MRPL37 |  IMG1 |  MRPL35 |  MRPL25 |  MRP2 |  MRPL28 |  YPL183W-A |  AEP1 |  MRPL11 |  YNR036C |  RPL21A |  MRPL24 |  RSM19 |  MRPL13 |  RSM25 |  MRP10 |  CBS2 |  MRPL17 |  MRPS8 |  MRPL7 |  MRPL40 |  MRPL6 |  YGR054W |  MEF2 |  MEF1 |  MRPL51 |  SLS1 |  MRPS35 |  RSM24 |  MRPL22 |  PET309 |  MRPL9 |  PET112 |  MRPL20 |  MSE1 |  MSM1 |  ISM1 |  NAM2 |  DIA4 | | | |
| GO:0006412 | protein biosynthesis | 1.518e-31 | E |
| GO:0009059 | macromolecule biosynthesis | 1.518e-31 | E |
| GO:0043284 | biopolymer biosynthesis | 1.518e-31 | E |
| GO:0019538 | protein metabolism | 1.758e-23 | E |
| GO:0043283 | biopolymer metabolism | 8.152e-17 | E |

  
  

|  |  |  |  |
| --- | --- | --- | --- |
| Class 10 | size: 3 gene products | | | |
| YGR054W |  MEF2 |  MEF1 | | | |
| GO:0043037 | translation | 0.000267 | E |

  
  

|  |  |  |  |
| --- | --- | --- | --- |
| Class 11 | size: 3 gene products | | | |
| CYT1 |  QCR8 |  QCR7 | | | |
| GO:0006122 | mitochondrial electron transport, ubiquinol to cytochrome c | 4.763e-06 | E |
| GO:0042773 | ATP synthesis coupled electron transport | 4.763e-06 | E |
| GO:0042775 | ATP synthesis coupled electron transport (sensu Eukaryota) | 4.763e-06 | E |
| GO:0006118 | electron transport | 4.763e-06 | E |
| GO:0006119 | oxidative phosphorylation | 1.905e-05 | E |
| GO:0006796 | phosphate metabolism | 9.527e-05 | E |
| GO:0006793 | phosphorus metabolism | 9.527e-05 | E |
| GO:0016310 | phosphorylation | 9.527e-05 | E |

  
  

|  |  |  |  |
| --- | --- | --- | --- |
| Class 12 | size: 6 gene products | | | |
| STB5 |  YAP1 |  SKN7 |  URE2 |  GSH1 |  LCB5 | | | |
| GO:0009628 | response to abiotic stimulus | 4.938e-10 | E |
| GO:0009605 | response to external stimulus | 4.938e-10 | E |
| GO:0050896 | response to stimulus | 4.148e-08 | E |

  
  

|  |  |  |  |
| --- | --- | --- | --- |
| Class 13 | size: 68 gene products | | | |
| MTF1 |  GAL11 |  SIN4 |  SRB5 |  ROX3 |  PAF1 |  MSS116 |  AEP3 |  SUV3 |  ATP12 |  CBP3 |  EUG1 |  COQ6 |  COQ1 |  CYT1 |  QCR8 |  QCR7 |  COR1 |  GND1 |  TKL1 |  RPE1 |  MTF2 |  CBS1 |  MSF1 |  MRPL16 |  MRPS16 |  MRPL37 |  IMG1 |  MRPL35 |  MRPL25 |  MRP2 |  MRPL28 |  YPL183W-A |  AEP1 |  MRPL11 |  YNR036C |  RPL21A |  MRPL24 |  RSM19 |  MRPL13 |  RSM25 |  MRP10 |  CBS2 |  MRPL17 |  MRPS8 |  MRPL7 |  MRPL40 |  MRPL6 |  YGR054W |  MEF2 |  MEF1 |  MRPL51 |  SLS1 |  MRPS35 |  RSM24 |  MRPL22 |  PET309 |  MRPL9 |  PET112 |  MRPL20 |  MSE1 |  MSM1 |  ISM1 |  NAM2 |  DIA4 |  IDP1 |  RTT109 |  REG1 | | | |
| GO:0008152 | metabolism | 3.670e-14 | E |

  
  

|  |  |  |  |
| --- | --- | --- | --- |
| Class 14 | size: 95 gene products | | | |
| MTF1 |  GAL11 |  SIN4 |  SRB5 |  ROX3 |  PAF1 |  MSS116 |  AEP3 |  SUV3 |  ATP12 |  CBP3 |  EUG1 |  COQ6 |  COQ1 |  CYT1 |  QCR8 |  QCR7 |  COR1 |  GND1 |  TKL1 |  RPE1 |  MTF2 |  CBS1 |  MSF1 |  MRPL16 |  MRPS16 |  MRPL37 |  IMG1 |  MRPL35 |  MRPL25 |  MRP2 |  MRPL28 |  YPL183W-A |  AEP1 |  MRPL11 |  YNR036C |  RPL21A |  MRPL24 |  RSM19 |  MRPL13 |  RSM25 |  MRP10 |  CBS2 |  MRPL17 |  MRPS8 |  MRPL7 |  MRPL40 |  MRPL6 |  YGR054W |  MEF2 |  MEF1 |  MRPL51 |  SLS1 |  MRPS35 |  RSM24 |  MRPL22 |  PET309 |  MRPL9 |  PET112 |  MRPL20 |  MSE1 |  MSM1 |  ISM1 |  NAM2 |  DIA4 |  IDP1 |  RTT109 |  REG1 |  STB5 |  YAP1 |  SKN7 |  URE2 |  GSH1 |  LCB5 |  VPS75 |  PCP1 |  ARV1 |  BAP2 |  GLO3 |  RIM1 |  HMI1 |  ABF2 |  MGM101 |  FAB1 |  HFI1 |  SNF2 |  PIF1 |  CCC1 |  GGC1 |  MBP1 |  SSQ1 |  MCK1 |  DBF2 |  SLA1 |  ATP5 | | | |
| GO:0007582 | physiological process | 6.237e-18 | E |

  
  

|  |  |  |  |
| --- | --- | --- | --- |
| Class 15 | size: 5 gene products | | | |
| VPS75 |  PCP1 |  ARV1 |  BAP2 |  GLO3 | | | |
| GO:0051234 | establishment of localization | 4.793e-07 | E |
| GO:0006810 | transport | 4.793e-07 | E |
| GO:0051179 | localization | 1.079e-06 | E |

  
  

|  |  |  |  |
| --- | --- | --- | --- |
| Class 16 | size: 6 gene products | | | |
| MSS116 |  AEP3 |  SUV3 |  ATP12 |  CBP3 |  EUG1 | | | |
| GO:0043283 | biopolymer metabolism | 0.027420 | E |

  
  

|  |  |  |  |
| --- | --- | --- | --- |
| Class 17 | size: 6 gene products | | | |
| MRPL51 |  SLS1 |  MRPS35 |  RSM24 |  MRPL22 |  PET309 | | | |
| GO:0045333 | cellular respiration | 2.281e-07 | E |
| GO:0009060 | aerobic respiration | 2.281e-07 | E |
| GO:0015980 | energy derivation by oxidation of organic compounds | 2.472e-06 | E |
| GO:0006091 | energy pathways | 2.472e-06 | E |

  
  

|  |  |  |  |
| --- | --- | --- | --- |
| Class 18 | size: 109 gene products | | | |
| MTF1 |  GAL11 |  SIN4 |  SRB5 |  ROX3 |  PAF1 |  MSS116 |  AEP3 |  SUV3 |  ATP12 |  CBP3 |  EUG1 |  COQ6 |  COQ1 |  CYT1 |  QCR8 |  QCR7 |  COR1 |  GND1 |  TKL1 |  RPE1 |  MTF2 |  CBS1 |  MSF1 |  MRPL16 |  MRPS16 |  MRPL37 |  IMG1 |  MRPL35 |  MRPL25 |  MRP2 |  MRPL28 |  YPL183W-A |  AEP1 |  MRPL11 |  YNR036C |  RPL21A |  MRPL24 |  RSM19 |  MRPL13 |  RSM25 |  MRP10 |  CBS2 |  MRPL17 |  MRPS8 |  MRPL7 |  MRPL40 |  MRPL6 |  YGR054W |  MEF2 |  MEF1 |  MRPL51 |  SLS1 |  MRPS35 |  RSM24 |  MRPL22 |  PET309 |  MRPL9 |  PET112 |  MRPL20 |  MSE1 |  MSM1 |  ISM1 |  NAM2 |  DIA4 |  IDP1 |  RTT109 |  REG1 |  STB5 |  YAP1 |  SKN7 |  URE2 |  GSH1 |  LCB5 |  VPS75 |  PCP1 |  ARV1 |  BAP2 |  GLO3 |  RIM1 |  HMI1 |  ABF2 |  MGM101 |  FAB1 |  HFI1 |  SNF2 |  PIF1 |  CCC1 |  GGC1 |  MBP1 |  SSQ1 |  MCK1 |  DBF2 |  SLA1 |  ATP5 |  YGR102C |  YML036W |  SOV1 |  YNL080C |  YOR305W |  HSP31 |  TVP38 |  YPR116W |  YDR332W |  DEM1 |  YGR150C |  FMP53 |  YLR149C |  MTO2 | | | |
| GO:0008150 | biological\_process | 1.000000 | E |

  
  

|  |  |  |  |
| --- | --- | --- | --- |
| Class 19 | size: 6 gene products | | | |
| MTF1 |  GAL11 |  SIN4 |  SRB5 |  ROX3 |  PAF1 | | | |
| GO:0006351 | transcription, DNA-dependent | 1.383e-08 | E |
| GO:0006350 | transcription | 1.037e-07 | E |
| GO:0006139 | nucleobase, nucleoside, nucleotide and nucleic acid metabolism | 0.000293 | E |

  
  

|  |  |  |  |
| --- | --- | --- | --- |
| Class 20 | size: 4 gene products | | | |
| RIM1 |  HMI1 |  ABF2 |  MGM101 | | | |
| GO:0000002 | mitochondrial genome maintenance | 2.696e-06 | E |
| GO:0007005 | mitochondrion organization and biogenesis | 1.258e-05 | E |

  
  

|  |  |  |  |
| --- | --- | --- | --- |
| Class 21 | size: 3 gene products | | | |
| MSS116 |  AEP3 |  SUV3 | | | |
| GO:0016070 | RNA metabolism | 0.000572 | E |
| GO:0006139 | nucleobase, nucleoside, nucleotide and nucleic acid metabolism | 0.019339 | E |

  
  

|  |  |  |  |
| --- | --- | --- | --- |
| Class 22 | size: 21 gene products | | | |
| VPS75 |  PCP1 |  ARV1 |  BAP2 |  GLO3 |  RIM1 |  HMI1 |  ABF2 |  MGM101 |  FAB1 |  HFI1 |  SNF2 |  PIF1 |  CCC1 |  GGC1 |  MBP1 |  SSQ1 |  MCK1 |  DBF2 |  SLA1 |  ATP5 | | | |
| GO:0008151 | cell growth and/or maintenance | 8.303e-19 | E |
| GO:0050875 | cellular physiological process | 8.303e-19 | E |
| GO:0009987 | cellular process | 4.317e-18 | E |

  
  

|  |  |  |  |
| --- | --- | --- | --- |
| Class 23 | size: 3 gene products | | | |
| HFI1 |  SNF2 |  PIF1 | | | |
| GO:0007001 | chromosome organization and biogenesis (sensu Eukaryota) | 4.763e-06 | E |
| GO:0051276 | chromosome organization and biogenesis | 4.763e-06 | E |
| GO:0006259 | DNA metabolism | 0.000267 | E |
| GO:0006139 | nucleobase, nucleoside, nucleotide and nucleic acid metabolism | 0.019339 | E |
| GO:0043283 | biopolymer metabolism | 0.171435 | E |
| GO:0008152 | metabolism | 0.453876 | E |

  
  
