# Supplementary material for: Theme discovery from gene lists for identification and viewing of multiple functional groups
Source: BMC Bioinformatics. 2005 Jun 29;6:162. doi: 10.1186/1471-2105-6-162 (PMC1190153; doi:10.1186/1471-2105-6-162)
Supplement: Additional File 10 — GOToolBox outputs from analysis with H2O2 and itraconanzole datasets. Table 10 Files include the clustering results for H2O2 and itraconanzole datasets from GOToolBox. [file 1471-2105-6-162-S10.zip › gotbx-H2O2-data--MF-default.htm]

GOToolBox


|  |
| --- |
| GO-Proxy : GO-based Gene Clustering |
| Home | Create-Dataset | Store-Ref | GO-Stats | GO-Proxy | GO-Family | Help | |

**The program has found 24 Classes**

MATRIX\_FILE

|  |  |  |  |
| --- | --- | --- | --- |
| Class 1 | size: 28 gene products | | | |
| MRPL22 |  MRP2 |  MRPL25 |  MRPL11 |  MRPS35 |  MRPL17 |  MRPL7 |  MRPL35 |  MRPL13 |  RSM24 |  RSM19 |  MRPL40 |  MRPS8 |  MRPL20 |  MRPL51 |  MRPL24 |  YNR036C |  YPL183W-A |  MRPL28 |  MRP10 |  MRPL6 |  MRPL37 |  RSM25 |  IMG1 |  MRPS16 |  RPL21A |  MRPL16 |  MRPL9 | | | |
| GO:0003735 | structural constituent of ribosome | 1.224e-26 | E |
| GO:0005198 | structural molecule activity | 3.550e-25 | E |

  
  

|  |  |  |  |
| --- | --- | --- | --- |
| Class 2 | size: 3 gene products | | | |
| MTF1 |  HFI1 |  URE2 | | | |
| GO:0008134 | transcription factor binding | 4.763e-06 | E |
| GO:0003712 | transcription cofactor activity | 4.763e-06 | E |
| GO:0030528 | transcription regulator activity | 0.001362 | E |

  
  

|  |  |  |  |
| --- | --- | --- | --- |
| Class 3 | size: 6 gene products | | | |
| MSM1 |  DIA4 |  MSF1 |  MSE1 |  ISM1 |  NAM2 | | | |
| GO:0008452 | RNA ligase activity | 4.938e-10 | E |
| GO:0016876 | ligase activity, forming aminoacyl-tRNA and related compounds | 4.938e-10 | E |
| GO:0016886 | ligase activity, forming phosphoric ester bonds | 4.938e-10 | E |
| GO:0016875 | ligase activity, forming carbon-oxygen bonds | 4.938e-10 | E |
| GO:0004812 | tRNA ligase activity | 4.938e-10 | E |
| GO:0016874 | ligase activity | 3.457e-09 | E |
| GO:0003824 | catalytic activity | 0.000547 | E |

  
  

|  |  |  |  |
| --- | --- | --- | --- |
| Class 4 | size: 19 gene products | | | |
| SSQ1 |  ATP12 |  HSP31 |  SLA1 |  MTF1 |  HFI1 |  URE2 |  MBP1 |  STB5 |  YAP1 |  SKN7 |  MTF2 |  ABF2 |  MGM101 |  RIM1 |  MEF1 |  MEF2 |  CBS1 |  YGR054W | | | |
| GO:0005488 | binding | 2.503e-20 | E |

  
  

|  |  |  |  |
| --- | --- | --- | --- |
| Class 5 | size: 17 gene products | | | |
| PIF1 |  YDR332W |  MSS116 |  PCP1 |  COQ1 |  TKL1 |  GSH1 |  EUG1 |  RPE1 |  COQ6 |  GND1 |  IDP1 |  MCK1 |  DBF2 |  LCB5 |  FAB1 |  MTO2 | | | |
| GO:0003824 | catalytic activity | 3.575e-11 | E |

  
  

|  |  |  |  |
| --- | --- | --- | --- |
| Class 6 | size: 12 gene products | | | |
| MBP1 |  STB5 |  YAP1 |  SKN7 |  MTF2 |  ABF2 |  MGM101 |  RIM1 |  MEF1 |  MEF2 |  CBS1 |  YGR054W | | | |
| GO:0003676 | nucleic acid binding | 4.149e-15 | E |

  
  

|  |  |  |  |
| --- | --- | --- | --- |
| Class 7 | size: 7 gene products | | | |
| QCR7 |  QCR8 |  COR1 |  ATP5 |  BAP2 |  CYT1 |  GGC1 | | | |
| GO:0005215 | transporter activity | 3.356e-11 | E |

  
  

|  |  |  |  |
| --- | --- | --- | --- |
| Class 8 | size: 4 gene products | | | |
| QCR7 |  QCR8 |  COR1 |  ATP5 | | | |
| GO:0015078 | hydrogen ion transporter activity | 1.798e-07 | E |
| GO:0015075 | ion transporter activity | 1.798e-07 | E |
| GO:0008324 | cation transporter activity | 1.798e-07 | E |
| GO:0015077 | monovalent inorganic cation transporter activity | 1.798e-07 | E |

  
  

|  |  |  |  |
| --- | --- | --- | --- |
| Class 9 | size: 109 gene products | | | |
| MRPL22 |  MRP2 |  MRPL25 |  MRPL11 |  MRPS35 |  MRPL17 |  MRPL7 |  MRPL35 |  MRPL13 |  RSM24 |  RSM19 |  MRPL40 |  MRPS8 |  MRPL20 |  MRPL51 |  MRPL24 |  YNR036C |  YPL183W-A |  MRPL28 |  MRP10 |  MRPL6 |  MRPL37 |  RSM25 |  IMG1 |  MRPS16 |  RPL21A |  MRPL16 |  MRPL9 |  PIF1 |  YDR332W |  MSS116 |  PCP1 |  COQ1 |  TKL1 |  GSH1 |  EUG1 |  RPE1 |  COQ6 |  GND1 |  IDP1 |  MCK1 |  DBF2 |  LCB5 |  FAB1 |  MTO2 |  VPS75 |  PET112 |  YLR149C |  SLS1 |  YGR102C |  AEP1 |  SOV1 |  YML036W |  DEM1 |  YPR116W |  YGR150C |  CBP3 |  CCC1 |  AEP3 |  TVP38 |  FMP53 |  RTT109 |  CBS2 |  YOR305W |  YNL080C |  ARV1 |  PET309 |  PAF1 |  SRB5 |  ROX3 |  GAL11 |  SIN4 |  SNF2 |  SSQ1 |  ATP12 |  HSP31 |  SLA1 |  MTF1 |  HFI1 |  URE2 |  MBP1 |  STB5 |  YAP1 |  SKN7 |  MTF2 |  ABF2 |  MGM101 |  RIM1 |  MEF1 |  MEF2 |  CBS1 |  YGR054W |  MSM1 |  DIA4 |  MSF1 |  MSE1 |  ISM1 |  NAM2 |  SUV3 |  HMI1 |  REG1 |  GLO3 |  QCR7 |  QCR8 |  COR1 |  ATP5 |  BAP2 |  CYT1 |  GGC1 | | | |
| GO:0003674 | molecular\_function | 1.000000 | E |

  
  

|  |  |  |  |
| --- | --- | --- | --- |
| Class 10 | size: 7 gene products | | | |
| SSQ1 |  ATP12 |  HSP31 |  SLA1 |  MTF1 |  HFI1 |  URE2 | | | |
| GO:0005515 | protein binding | 3.356e-11 | E |

  
  

|  |  |  |  |
| --- | --- | --- | --- |
| Class 11 | size: 21 gene products | | | |
| VPS75 |  PET112 |  YLR149C |  SLS1 |  YGR102C |  AEP1 |  SOV1 |  YML036W |  DEM1 |  YPR116W |  YGR150C |  CBP3 |  CCC1 |  AEP3 |  TVP38 |  FMP53 |  RTT109 |  CBS2 |  YOR305W |  YNL080C |  ARV1 | | | |
| GO:0005554 | molecular\_function unknown | 6.563e-23 | E |

  
  

|  |  |  |  |
| --- | --- | --- | --- |
| Class 12 | size: 5 gene products | | | |
| SRB5 |  ROX3 |  GAL11 |  SIN4 |  SNF2 | | | |
| GO:0003702 | RNA polymerase II transcription factor activity | 8.560e-09 | E |
| GO:0016251 | general RNA polymerase II transcription factor activity | 8.560e-09 | E |

  
  

|  |  |  |  |
| --- | --- | --- | --- |
| Class 13 | size: 5 gene products | | | |
| MCK1 |  DBF2 |  LCB5 |  FAB1 |  MTO2 | | | |
| GO:0016740 | transferase activity | 2.157e-06 | E |

  
  

|  |  |  |  |
| --- | --- | --- | --- |
| Class 14 | size: 3 gene products | | | |
| PIF1 |  YDR332W |  MSS116 | | | |
| GO:0004386 | helicase activity | 4.763e-05 | E |

  
  

|  |  |  |  |
| --- | --- | --- | --- |
| Class 15 | size: 3 gene products | | | |
| SSQ1 |  ATP12 |  HSP31 | | | |
| GO:0051082 | unfolded protein binding | 4.763e-06 | E |

  
  

|  |  |  |  |
| --- | --- | --- | --- |
| Class 16 | size: 4 gene products | | | |
| MBP1 |  STB5 |  YAP1 |  SKN7 | | | |
| GO:0003700 | transcription factor activity | 1.798e-07 | E |
| GO:0003677 | DNA binding | 6.291e-06 | E |
| GO:0030528 | transcription regulator activity | 0.000129 | E |

  
  

|  |  |  |  |
| --- | --- | --- | --- |
| Class 17 | size: 6 gene products | | | |
| PAF1 |  SRB5 |  ROX3 |  GAL11 |  SIN4 |  SNF2 | | | |
| GO:0030528 | transcription regulator activity | 8.474e-07 | E |

  
  

|  |  |  |  |
| --- | --- | --- | --- |
| Class 18 | size: 4 gene products | | | |
| MCK1 |  DBF2 |  LCB5 |  FAB1 | | | |
| GO:0016772 | transferase activity, transferring phosphorus-containing groups | 1.798e-07 | E |
| GO:0016301 | kinase activity | 1.798e-07 | E |

  
  

|  |  |  |  |
| --- | --- | --- | --- |
| Class 19 | size: 4 gene products | | | |
| SRB5 |  ROX3 |  GAL11 |  SIN4 | | | |
| GO:0016455 | RNA polymerase II transcription mediator activity | 1.798e-07 | E |

  
  

|  |  |  |  |
| --- | --- | --- | --- |
| Class 20 | size: 4 gene products | | | |
| MEF1 |  MEF2 |  CBS1 |  YGR054W | | | |
| GO:0008135 | translation factor activity, nucleic acid binding | 1.798e-07 | E |
| GO:0045182 | translation regulator activity | 8.988e-07 | E |

  
  

|  |  |  |  |
| --- | --- | --- | --- |
| Class 21 | size: 3 gene products | | | |
| ABF2 |  MGM101 |  RIM1 | | | |
| GO:0003677 | DNA binding | 0.000167 | E |

  
  

|  |  |  |  |
| --- | --- | --- | --- |
| Class 22 | size: 3 gene products | | | |
| SUV3 |  HMI1 |  REG1 | | | |
| GO:0016787 | hydrolase activity | 4.763e-05 | E |
| GO:0003824 | catalytic activity | 0.025989 | E |

  
  

|  |  |  |  |
| --- | --- | --- | --- |
| Class 23 | size: 3 gene products | | | |
| QCR7 |  QCR8 |  COR1 | | | |
| GO:0015399 | primary active transporter activity | 4.763e-06 | E |
| GO:0016681 | oxidoreductase activity, acting on diphenols and related substances as donors, cytochrome as acceptor | 4.763e-06 | E |
| GO:0016679 | oxidoreductase activity, acting on diphenols and related substances as donors | 4.763e-06 | E |
| GO:0005386 | carrier activity | 4.763e-06 | E |
| GO:0008121 | ubiquinol-cytochrome-c reductase activity | 4.763e-06 | E |
| GO:0016491 | oxidoreductase activity | 9.527e-05 | E |
| GO:0003824 | catalytic activity | 0.025989 | E |

  
  

|  |  |  |  |
| --- | --- | --- | --- |
| Class 24 | size: 3 gene products | | | |
| COQ6 |  GND1 |  IDP1 | | | |
| GO:0016491 | oxidoreductase activity | 9.527e-05 | E |

  
  
