# Supplementary material for: Theme discovery from gene lists for identification and viewing of multiple functional groups
Source: BMC Bioinformatics. 2005 Jun 29;6:162. doi: 10.1186/1471-2105-6-162 (PMC1190153; doi:10.1186/1471-2105-6-162)
Supplement: Additional File 10 — GOToolBox outputs from analysis with H2O2 and itraconanzole datasets. Table 10 Files include the clustering results for H2O2 and itraconanzole datasets from GOToolBox. [file 1471-2105-6-162-S10.zip › gotbx-itra-data-BP-default.htm]

GOToolBox


|  |
| --- |
| GO-Proxy : GO-based Gene Clustering |
| Home | Create-Dataset | Store-Ref | GO-Stats | GO-Proxy | GO-Family | Help | |

**The program has found 32 Classes**

MATRIX\_FILE

|  |  |  |  |
| --- | --- | --- | --- |
| Class 1 | size: 249 gene products | | | |
| MSB3 |  HUA1 |  PIN3 |  PST1 |  CWP1 |  SED1 |  YHR138C |  SPC42 |  KRR1 |  HAS1 |  UTP30 |  UTP4 |  FYV7 |  FAP7 |  HTA2 |  YPL033C |  REC8 |  WTM1 |  PDS5 |  MCD1 |  SWE1 |  PCL5 |  SCM4 |  CTF13 |  BNI5 |  CHS1 |  NVJ1 |  TSA2 |  DAN1 |  AUS1 |  RSB1 |  VHT1 |  TPN1 |  PHO3 |  MCH1 |  GYP7 |  ERP3 |  HXT2 |  BAP2 |  AGP1 |  GAP1 |  PHO89 |  SSU1 |  ZRT1 |  ENB1 |  ENA5 |  SGE1 |  ATR1 |  SRO77 |  ECM1 |  PEX21 |  BSD2 |  YPT53 |  YFR055W |  ISU1 |  GGC1 |  HBT1 |  AGA1 |  AFR1 |  PRM4 |  MSB2 |  GIC2 |  CDC11 |  MAC1 |  UGA3 |  SPT21 |  CAF16 |  STP2 |  PDR8 |  HAC1 |  PIP2 |  RIM101 |  IRS4 |  MSN2 |  POL30 |  YNL311C |  CPS1 |  AAP1prime |  NTH1 |  YOR302W |  MNT4 |  SSB1 |  SSZ1 |  MRPL49 |  TUF1 |  TEF4 |  TIF35 |  HSP26 |  ULA1 |  YPS1 |  YPS3 |  PPT1 |  SLI15 |  IST3 |  RDS1 |  TIR2 |  TIR1 |  NCE103 |  PHO5 |  PET10 |  STR2 |  OPT1 |  YPL088W |  AAD16 |  GCV2 |  ADH5 |  GDH2 |  AMS1 |  FUR1 |  SRL3 |  TEA1 |  PDH1 |  PDX3 |  NCR1 |  PLB1 |  DAP1 |  ATF2 |  SRY1 |  YJL113W |  RTG3 |  CUP2 |  ERG2 |  ERG25 |  ERG27 |  ERG28 |  ERG3 |  ERG11 |  ERG5 |  ERG1 |  ERG6 |  CYB5 |  UPC2 |  HES1 |  CHO1 |  SUR2 |  GPI11 |  SUR4 |  ELO1 |  SIP4 |  PYC1 |  PSA1 |  URA10 |  SDT1 |  AAH1 |  ADE3 |  FOL2 |  RIB3 |  RIB1 |  RIB5 |  SNO1 |  BIO3 |  BNA1 |  STF1 |  ATP15 |  ADE17 |  ARO8 |  ARO4 |  ARO1 |  HOM3 |  SER3 |  LYS1 |  LYS2 |  ILV3 |  ILV2 |  LEU4 |  LEU1 |  BAT2 |  BAT1 |  HIS4 |  HIS5 |  HIS1 |  HIS3 |  SER1 |  BAS1 |  TRP3 |  MET16 |  MET22 |  ARO10 |  UGA2 |  CIT2 |  IDP1 |  AAT2 |  ARG4 |  CPA1 |  CPA2 |  ARG8 |  ARG3 |  ARG1 |  ECM40 |  ARG5 |  6 |  ORT1 |  YIL056W |  YPL282C |  YOR385W |  BOP2 |  RCR1 |  YER158C |  YIR041W |  YJL213W |  YNL208W |  YET2 |  YOR289W |  YKL121W |  YDR222W |  ICY1 |  YNR068C |  ALT1 |  YJR111C |  YOR394W |  YJL206C |  YLR194C |  YGL117W |  YJL016W |  YGR110W |  YET1 |  UBX6 |  YDR542W |  YGR043C |  YEH1 |  BSC5 |  HSP33 |  YPL272C |  BCP1 |  YMR009W |  FMP23 |  YIL158W |  FMP12 |  NIT1 |  YJL171C |  YKL224C |  YOR338W |  YBR147W |  YHR029C |  YHR127W |  BOP1 |  CRH1 |  DRE2 |  YLR152C |  YNL115C |  PHO12 |  ZTA1 |  GIR2 |  YER189W |  FMP43 |  YJL200C |  PRR2 |  COS111 |  DIA1 | | | |
| GO:0008150 | biological\_process | 0.000000 | E |

  
  

|  |  |  |  |
| --- | --- | --- | --- |
| Class 2 | size: 20 gene products | | | |
| ERG2 |  ERG25 |  ERG27 |  ERG28 |  ERG3 |  ERG11 |  ERG5 |  ERG1 |  ERG6 |  CYB5 |  UPC2 |  HES1 |  CHO1 |  SUR2 |  GPI11 |  SUR4 |  ELO1 |  SIP4 |  PYC1 |  PSA1 | | | |
| GO:0008610 | lipid biosynthesis | 0.000000 | E |
| GO:0006629 | lipid metabolism | 7.333e-26 | E |
| GO:0009059 | macromolecule biosynthesis | 2.145e-23 | E |
| GO:0043170 | macromolecule metabolism | 1.215e-17 | E |
| GO:0009058 | biosynthesis | 2.153e-12 | E |

  
  

|  |  |  |  |
| --- | --- | --- | --- |
| Class 3 | size: 12 gene products | | | |
| MAC1 |  UGA3 |  SPT21 |  CAF16 |  STP2 |  PDR8 |  HAC1 |  PIP2 |  RIM101 |  IRS4 |  MSN2 |  POL30 | | | |
| GO:0006355 | regulation of transcription, DNA-dependent | 0.000000 | E |
| GO:0045449 | regulation of transcription | 1.508e-19 | E |
| GO:0019219 | regulation of nucleobase, nucleoside, nucleotide and nucleic acid metabolism | 1.508e-19 | E |
| GO:0006351 | transcription, DNA-dependent | 1.055e-18 | E |
| GO:0019222 | regulation of metabolism | 1.055e-18 | E |
| GO:0050791 | regulation of physiological process | 5.276e-18 | E |
| GO:0050789 | regulation of biological process | 7.176e-17 | E |
| GO:0006350 | transcription | 7.176e-17 | E |
| GO:0006139 | nucleobase, nucleoside, nucleotide and nucleic acid metabolism | 9.160e-11 | E |
| GO:0008152 | metabolism | 0.000536 | E |

  
  

|  |  |  |  |
| --- | --- | --- | --- |
| Class 4 | size: 117 gene products | | | |
| YNL311C |  CPS1 |  AAP1prime |  NTH1 |  YOR302W |  MNT4 |  SSB1 |  SSZ1 |  MRPL49 |  TUF1 |  TEF4 |  TIF35 |  HSP26 |  ULA1 |  YPS1 |  YPS3 |  PPT1 |  SLI15 |  IST3 |  RDS1 |  TIR2 |  TIR1 |  NCE103 |  PHO5 |  PET10 |  STR2 |  OPT1 |  YPL088W |  AAD16 |  GCV2 |  ADH5 |  GDH2 |  AMS1 |  FUR1 |  SRL3 |  TEA1 |  PDH1 |  PDX3 |  NCR1 |  PLB1 |  DAP1 |  ATF2 |  SRY1 |  YJL113W |  RTG3 |  CUP2 |  ERG2 |  ERG25 |  ERG27 |  ERG28 |  ERG3 |  ERG11 |  ERG5 |  ERG1 |  ERG6 |  CYB5 |  UPC2 |  HES1 |  CHO1 |  SUR2 |  GPI11 |  SUR4 |  ELO1 |  SIP4 |  PYC1 |  PSA1 |  URA10 |  SDT1 |  AAH1 |  ADE3 |  FOL2 |  RIB3 |  RIB1 |  RIB5 |  SNO1 |  BIO3 |  BNA1 |  STF1 |  ATP15 |  ADE17 |  ARO8 |  ARO4 |  ARO1 |  HOM3 |  SER3 |  LYS1 |  LYS2 |  ILV3 |  ILV2 |  LEU4 |  LEU1 |  BAT2 |  BAT1 |  HIS4 |  HIS5 |  HIS1 |  HIS3 |  SER1 |  BAS1 |  TRP3 |  MET16 |  MET22 |  ARO10 |  UGA2 |  CIT2 |  IDP1 |  AAT2 |  ARG4 |  CPA1 |  CPA2 |  ARG8 |  ARG3 |  ARG1 |  ECM40 |  ARG5 |  6 |  ORT1 | | | |
| GO:0008152 | metabolism | 6.855e-52 | E |

  
  

|  |  |  |  |
| --- | --- | --- | --- |
| Class 5 | size: 15 gene products | | | |
| MSB3 |  HUA1 |  PIN3 |  PST1 |  CWP1 |  SED1 |  YHR138C |  SPC42 |  KRR1 |  HAS1 |  UTP30 |  UTP4 |  FYV7 |  FAP7 |  HTA2 | | | |
| GO:0006996 | organelle organization and biogenesis | 3.782e-20 | E |
| GO:0016043 | cell organization and biogenesis | 1.885e-17 | E |

  
  

|  |  |  |  |
| --- | --- | --- | --- |
| Class 6 | size: 28 gene products | | | |
| DAN1 |  AUS1 |  RSB1 |  VHT1 |  TPN1 |  PHO3 |  MCH1 |  GYP7 |  ERP3 |  HXT2 |  BAP2 |  AGP1 |  GAP1 |  PHO89 |  SSU1 |  ZRT1 |  ENB1 |  ENA5 |  SGE1 |  ATR1 |  SRO77 |  ECM1 |  PEX21 |  BSD2 |  YPT53 |  YFR055W |  ISU1 |  GGC1 | | | |
| GO:0051179 | localization | 6.027e-34 | E |
| GO:0006810 | transport | 6.027e-34 | E |
| GO:0051234 | establishment of localization | 6.027e-34 | E |

  
  

|  |  |  |  |
| --- | --- | --- | --- |
| Class 7 | size: 7 gene products | | | |
| HBT1 |  AGA1 |  AFR1 |  PRM4 |  MSB2 |  GIC2 |  CDC11 | | | |
| GO:0000003 | reproduction | 9.514e-14 | E |
| GO:0007275 | development | 3.140e-11 | E |

  
  

|  |  |  |  |
| --- | --- | --- | --- |
| Class 8 | size: 4 gene products | | | |
| HIS4 |  HIS5 |  HIS1 |  HIS3 | | | |
| GO:0009076 | histidine family amino acid biosynthesis | 3.250e-08 | E |
| GO:0009075 | histidine family amino acid metabolism | 3.250e-08 | E |
| GO:0006547 | histidine metabolism | 3.250e-08 | E |
| GO:0000105 | histidine biosynthesis | 3.250e-08 | E |

  
  

|  |  |  |  |
| --- | --- | --- | --- |
| Class 9 | size: 192 gene products | | | |
| MSB3 |  HUA1 |  PIN3 |  PST1 |  CWP1 |  SED1 |  YHR138C |  SPC42 |  KRR1 |  HAS1 |  UTP30 |  UTP4 |  FYV7 |  FAP7 |  HTA2 |  YPL033C |  REC8 |  WTM1 |  PDS5 |  MCD1 |  SWE1 |  PCL5 |  SCM4 |  CTF13 |  BNI5 |  CHS1 |  NVJ1 |  TSA2 |  DAN1 |  AUS1 |  RSB1 |  VHT1 |  TPN1 |  PHO3 |  MCH1 |  GYP7 |  ERP3 |  HXT2 |  BAP2 |  AGP1 |  GAP1 |  PHO89 |  SSU1 |  ZRT1 |  ENB1 |  ENA5 |  SGE1 |  ATR1 |  SRO77 |  ECM1 |  PEX21 |  BSD2 |  YPT53 |  YFR055W |  ISU1 |  GGC1 |  HBT1 |  AGA1 |  AFR1 |  PRM4 |  MSB2 |  GIC2 |  CDC11 |  MAC1 |  UGA3 |  SPT21 |  CAF16 |  STP2 |  PDR8 |  HAC1 |  PIP2 |  RIM101 |  IRS4 |  MSN2 |  POL30 |  YNL311C |  CPS1 |  AAP1prime |  NTH1 |  YOR302W |  MNT4 |  SSB1 |  SSZ1 |  MRPL49 |  TUF1 |  TEF4 |  TIF35 |  HSP26 |  ULA1 |  YPS1 |  YPS3 |  PPT1 |  SLI15 |  IST3 |  RDS1 |  TIR2 |  TIR1 |  NCE103 |  PHO5 |  PET10 |  STR2 |  OPT1 |  YPL088W |  AAD16 |  GCV2 |  ADH5 |  GDH2 |  AMS1 |  FUR1 |  SRL3 |  TEA1 |  PDH1 |  PDX3 |  NCR1 |  PLB1 |  DAP1 |  ATF2 |  SRY1 |  YJL113W |  RTG3 |  CUP2 |  ERG2 |  ERG25 |  ERG27 |  ERG28 |  ERG3 |  ERG11 |  ERG5 |  ERG1 |  ERG6 |  CYB5 |  UPC2 |  HES1 |  CHO1 |  SUR2 |  GPI11 |  SUR4 |  ELO1 |  SIP4 |  PYC1 |  PSA1 |  URA10 |  SDT1 |  AAH1 |  ADE3 |  FOL2 |  RIB3 |  RIB1 |  RIB5 |  SNO1 |  BIO3 |  BNA1 |  STF1 |  ATP15 |  ADE17 |  ARO8 |  ARO4 |  ARO1 |  HOM3 |  SER3 |  LYS1 |  LYS2 |  ILV3 |  ILV2 |  LEU4 |  LEU1 |  BAT2 |  BAT1 |  HIS4 |  HIS5 |  HIS1 |  HIS3 |  SER1 |  BAS1 |  TRP3 |  MET16 |  MET22 |  ARO10 |  UGA2 |  CIT2 |  IDP1 |  AAT2 |  ARG4 |  CPA1 |  CPA2 |  ARG8 |  ARG3 |  ARG1 |  ECM40 |  ARG5 |  6 |  ORT1 | | | |
| GO:0007582 | physiological process | 0.000000 | E |

  
  

|  |  |  |  |
| --- | --- | --- | --- |
| Class 10 | size: 4 gene products | | | |
| ECM1 |  PEX21 |  BSD2 |  YPT53 | | | |
| GO:0006605 | protein targeting | 6.501e-09 | E |
| GO:0045184 | establishment of protein localization | 6.501e-09 | E |
| GO:0015031 | protein transport | 6.501e-09 | E |
| GO:0006886 | intracellular protein transport | 6.501e-09 | E |
| GO:0008104 | protein localization | 6.501e-09 | E |
| GO:0046907 | intracellular transport | 2.275e-07 | E |

  
  

|  |  |  |  |
| --- | --- | --- | --- |
| Class 11 | size: 6 gene products | | | |
| YPL033C |  REC8 |  WTM1 |  PDS5 |  MCD1 |  SWE1 | | | |
| GO:0000280 | nuclear division | 2.302e-11 | E |
| GO:0000279 | M phase | 9.210e-11 | E |
| GO:0007049 | cell cycle | 5.644e-09 | E |

  
  

|  |  |  |  |
| --- | --- | --- | --- |
| Class 12 | size: 5 gene products | | | |
| URA10 |  SDT1 |  AAH1 |  ADE3 |  FOL2 | | | |
| GO:0009112 | nucleobase metabolism | 7.993e-10 | E |
| GO:0046483 | heterocycle metabolism | 1.678e-08 | E |
| GO:0006725 | aromatic compound metabolism | 1.055e-07 | E |
| GO:0006139 | nucleobase, nucleoside, nucleotide and nucleic acid metabolism | 9.983e-05 | E |

  
  

|  |  |  |  |
| --- | --- | --- | --- |
| Class 13 | size: 6 gene products | | | |
| ILV3 |  ILV2 |  LEU4 |  LEU1 |  BAT2 |  BAT1 | | | |
| GO:0009082 | branched chain family amino acid biosynthesis | 3.289e-12 | E |
| GO:0009081 | branched chain family amino acid metabolism | 2.302e-11 | E |

  
  

|  |  |  |  |
| --- | --- | --- | --- |
| Class 14 | size: 13 gene products | | | |
| CIT2 |  IDP1 |  AAT2 |  ARG4 |  CPA1 |  CPA2 |  ARG8 |  ARG3 |  ARG1 |  ECM40 |  ARG5 |  6 |  ORT1 | | | |
| GO:0009064 | glutamine family amino acid metabolism | 6.388e-22 | E |

  
  

|  |  |  |  |
| --- | --- | --- | --- |
| Class 15 | size: 8 gene products | | | |
| YOR302W |  MNT4 |  SSB1 |  SSZ1 |  MRPL49 |  TUF1 |  TEF4 |  TIF35 | | | |
| GO:0043284 | biopolymer biosynthesis | 1.421e-13 | E |
| GO:0006412 | protein biosynthesis | 1.421e-13 | E |
| GO:0009059 | macromolecule biosynthesis | 9.816e-09 | E |
| GO:0009058 | biosynthesis | 3.780e-05 | E |

  
  

|  |  |  |  |
| --- | --- | --- | --- |
| Class 16 | size: 75 gene products | | | |
| MSB3 |  HUA1 |  PIN3 |  PST1 |  CWP1 |  SED1 |  YHR138C |  SPC42 |  KRR1 |  HAS1 |  UTP30 |  UTP4 |  FYV7 |  FAP7 |  HTA2 |  YPL033C |  REC8 |  WTM1 |  PDS5 |  MCD1 |  SWE1 |  PCL5 |  SCM4 |  CTF13 |  BNI5 |  CHS1 |  NVJ1 |  TSA2 |  DAN1 |  AUS1 |  RSB1 |  VHT1 |  TPN1 |  PHO3 |  MCH1 |  GYP7 |  ERP3 |  HXT2 |  BAP2 |  AGP1 |  GAP1 |  PHO89 |  SSU1 |  ZRT1 |  ENB1 |  ENA5 |  SGE1 |  ATR1 |  SRO77 |  ECM1 |  PEX21 |  BSD2 |  YPT53 |  YFR055W |  ISU1 |  GGC1 |  HBT1 |  AGA1 |  AFR1 |  PRM4 |  MSB2 |  GIC2 |  CDC11 |  MAC1 |  UGA3 |  SPT21 |  CAF16 |  STP2 |  PDR8 |  HAC1 |  PIP2 |  RIM101 |  IRS4 |  MSN2 |  POL30 | | | |
| GO:0008151 | cell growth and/or maintenance | 0.000000 | E |
| GO:0050875 | cellular physiological process | 5.162e-62 | E |
| GO:0009987 | cellular process | 4.241e-58 | E |

  
  

|  |  |  |  |
| --- | --- | --- | --- |
| Class 17 | size: 10 gene products | | | |
| ARG4 |  CPA1 |  CPA2 |  ARG8 |  ARG3 |  ARG1 |  ECM40 |  ARG5 |  6 |  ORT1 | | | |
| GO:0006525 | arginine metabolism | 0.000000 | E |
| GO:0000051 | urea cycle intermediate metabolism | 0.000000 | E |
| GO:0006526 | arginine biosynthesis | 0.000000 | E |
| GO:0009084 | glutamine family amino acid biosynthesis | 5.451e-17 | E |
| GO:0006807 | nitrogen metabolism | 1.417e-15 | E |

  
  

|  |  |  |  |
| --- | --- | --- | --- |
| Class 18 | size: 12 gene products | | | |
| ERG2 |  ERG25 |  ERG27 |  ERG28 |  ERG3 |  ERG11 |  ERG5 |  ERG1 |  ERG6 |  CYB5 |  UPC2 |  HES1 | | | |
| GO:0016126 | sterol biosynthesis | 0.000000 | E |
| GO:0016125 | sterol metabolism | 1.160e-20 | E |
| GO:0006066 | alcohol metabolism | 2.111e-17 | E |

  
  

|  |  |  |  |
| --- | --- | --- | --- |
| Class 19 | size: 6 gene products | | | |
| RIB3 |  RIB1 |  RIB5 |  SNO1 |  BIO3 |  BNA1 | | | |
| GO:0009110 | vitamin biosynthesis | 3.289e-12 | E |
| GO:0042364 | water-soluble vitamin biosynthesis | 3.289e-12 | E |
| GO:0006767 | water-soluble vitamin metabolism | 2.302e-11 | E |
| GO:0006766 | vitamin metabolism | 2.302e-11 | E |

  
  

|  |  |  |  |
| --- | --- | --- | --- |
| Class 20 | size: 7 gene products | | | |
| KRR1 |  HAS1 |  UTP30 |  UTP4 |  FYV7 |  FAP7 |  HTA2 | | | |
| GO:0016072 | rRNA metabolism | 0.000000 | E |
| GO:0006364 | rRNA processing | 0.000000 | E |
| GO:0007046 | ribosome biogenesis | 9.514e-14 | E |
| GO:0016070 | RNA metabolism | 9.514e-14 | E |
| GO:0007028 | cytoplasm organization and biogenesis | 9.514e-14 | E |
| GO:0006396 | RNA processing | 9.514e-14 | E |
| GO:0042254 | ribosome biogenesis and assembly | 9.514e-14 | E |
| GO:0043283 | biopolymer metabolism | 3.202e-07 | E |
| GO:0006139 | nucleobase, nucleoside, nucleotide and nucleic acid metabolism | 2.139e-06 | E |
| GO:0008152 | metabolism | 0.013171 | E |

  
  

|  |  |  |  |
| --- | --- | --- | --- |
| Class 21 | size: 13 gene products | | | |
| YPL033C |  REC8 |  WTM1 |  PDS5 |  MCD1 |  SWE1 |  PCL5 |  SCM4 |  CTF13 |  BNI5 |  CHS1 |  NVJ1 |  TSA2 | | | |
| GO:0008283 | cell proliferation | 1.520e-18 | E |

  
  

|  |  |  |  |
| --- | --- | --- | --- |
| Class 22 | size: 57 gene products | | | |
| YIL056W |  YPL282C |  YOR385W |  BOP2 |  RCR1 |  YER158C |  YIR041W |  YJL213W |  YNL208W |  YET2 |  YOR289W |  YKL121W |  YDR222W |  ICY1 |  YNR068C |  ALT1 |  YJR111C |  YOR394W |  YJL206C |  YLR194C |  YGL117W |  YJL016W |  YGR110W |  YET1 |  UBX6 |  YDR542W |  YGR043C |  YEH1 |  BSC5 |  HSP33 |  YPL272C |  BCP1 |  YMR009W |  FMP23 |  YIL158W |  FMP12 |  NIT1 |  YJL171C |  YKL224C |  YOR338W |  YBR147W |  YHR029C |  YHR127W |  BOP1 |  CRH1 |  DRE2 |  YLR152C |  YNL115C |  PHO12 |  ZTA1 |  GIR2 |  YER189W |  FMP43 |  YJL200C |  PRR2 |  COS111 |  DIA1 | | | |
| GO:0000004 | biological\_process unknown | 0.000000 | E |

  
  

|  |  |  |  |
| --- | --- | --- | --- |
| Class 23 | size: 14 gene products | | | |
| URA10 |  SDT1 |  AAH1 |  ADE3 |  FOL2 |  RIB3 |  RIB1 |  RIB5 |  SNO1 |  BIO3 |  BNA1 |  STF1 |  ATP15 |  ADE17 | | | |
| GO:0009058 | biosynthesis | 1.137e-08 | E |

  
  

|  |  |  |  |
| --- | --- | --- | --- |
| Class 24 | size: 37 gene products | | | |
| ARO8 |  ARO4 |  ARO1 |  HOM3 |  SER3 |  LYS1 |  LYS2 |  ILV3 |  ILV2 |  LEU4 |  LEU1 |  BAT2 |  BAT1 |  HIS4 |  HIS5 |  HIS1 |  HIS3 |  SER1 |  BAS1 |  TRP3 |  MET16 |  MET22 |  ARO10 |  UGA2 |  CIT2 |  IDP1 |  AAT2 |  ARG4 |  CPA1 |  CPA2 |  ARG8 |  ARG3 |  ARG1 |  ECM40 |  ARG5 |  6 |  ORT1 | | | |
| GO:0008652 | amino acid biosynthesis | 0.000000 | E |
| GO:0009309 | amine biosynthesis | 0.000000 | E |
| GO:0006520 | amino acid metabolism | 0.000000 | E |
| GO:0009308 | amine metabolism | 0.000000 | E |
| GO:0006519 | amino acid and derivative metabolism | 5.918e-45 | E |
| GO:0019752 | carboxylic acid metabolism | 2.268e-37 | E |
| GO:0006082 | organic acid metabolism | 2.268e-37 | E |
| GO:0009058 | biosynthesis | 2.548e-24 | E |

  
  

|  |  |  |  |
| --- | --- | --- | --- |
| Class 25 | size: 19 gene products | | | |
| YNL311C |  CPS1 |  AAP1prime |  NTH1 |  YOR302W |  MNT4 |  SSB1 |  SSZ1 |  MRPL49 |  TUF1 |  TEF4 |  TIF35 |  HSP26 |  ULA1 |  YPS1 |  YPS3 |  PPT1 |  SLI15 |  IST3 | | | |
| GO:0019538 | protein metabolism | 1.659e-26 | E |
| GO:0043283 | biopolymer metabolism | 2.745e-20 | E |
| GO:0043170 | macromolecule metabolism | 1.113e-16 | E |

  
  

|  |  |  |  |
| --- | --- | --- | --- |
| Class 26 | size: 4 gene products | | | |
| HBT1 |  AGA1 |  AFR1 |  PRM4 | | | |
| GO:0000746 | conjugation | 3.250e-08 | E |
| GO:0019953 | sexual reproduction | 3.250e-08 | E |
| GO:0000747 | conjugation with cellular fusion | 3.250e-08 | E |

  
  

|  |  |  |  |
| --- | --- | --- | --- |
| Class 27 | size: 4 gene products | | | |
| YNL311C |  CPS1 |  AAP1prime |  NTH1 | | | |
| GO:0009056 | catabolism | 3.218e-06 | E |

  
  

|  |  |  |  |
| --- | --- | --- | --- |
| Class 28 | size: 6 gene products | | | |
| UGA3 |  SPT21 |  CAF16 |  STP2 |  PDR8 |  HAC1 | | | |
| GO:0006357 | regulation of transcription from Pol II promoter | 9.210e-11 | E |
| GO:0006366 | transcription from Pol II promoter | 1.520e-09 | E |

  
  

|  |  |  |  |
| --- | --- | --- | --- |
| Class 29 | size: 5 gene products | | | |
| RDS1 |  TIR2 |  TIR1 |  NCE103 |  PHO5 | | | |
| GO:0006950 | response to stress | 2.667e-07 | E |
| GO:0050896 | response to stimulus | 3.508e-06 | E |

  
  

|  |  |  |  |
| --- | --- | --- | --- |
| Class 30 | size: 11 gene products | | | |
| ERG2 |  ERG25 |  ERG27 |  ERG28 |  ERG3 |  ERG11 |  ERG5 |  ERG1 |  ERG6 |  CYB5 |  UPC2 | | | |
| GO:0008204 | ergosterol metabolism | 0.000000 | E |
| GO:0006696 | ergosterol biosynthesis | 0.000000 | E |

  
  

|  |  |  |  |
| --- | --- | --- | --- |
| Class 31 | size: 15 gene products | | | |
| ERG2 |  ERG25 |  ERG27 |  ERG28 |  ERG3 |  ERG11 |  ERG5 |  ERG1 |  ERG6 |  CYB5 |  UPC2 |  HES1 |  CHO1 |  SUR2 |  GPI11 | | | |
| GO:0008202 | steroid metabolism | 0.000000 | E |
| GO:0006694 | steroid biosynthesis | 0.000000 | E |

  
  

|  |  |  |  |
| --- | --- | --- | --- |
| Class 32 | size: 6 gene products | | | |
| PDH1 |  PDX3 |  NCR1 |  PLB1 |  DAP1 |  ATF2 | | | |
| GO:0006629 | lipid metabolism | 4.427e-07 | E |

  
  
