# Supplementary material for: Theme discovery from gene lists for identification and viewing of multiple functional groups
Source: BMC Bioinformatics. 2005 Jun 29;6:162. doi: 10.1186/1471-2105-6-162 (PMC1190153; doi:10.1186/1471-2105-6-162)
Supplement: Additional File 10 — GOToolBox outputs from analysis with H2O2 and itraconanzole datasets. Table 10 Files include the clustering results for H2O2 and itraconanzole datasets from GOToolBox. [file 1471-2105-6-162-S10.zip › gotbx-itra-data-CL-default.htm]

GOToolBox


|  |
| --- |
| GO-Proxy : GO-based Gene Clustering |
| Home | Create-Dataset | Store-Ref | GO-Stats | GO-Proxy | GO-Family | Help | |

**The program has found 33 Classes**

MATRIX\_FILE

|  |  |  |  |
| --- | --- | --- | --- |
| Class 1 | size: 5 gene products | | | |
| YNL115C |  CPS1 |  PHO12 |  YIL158W |  BOP1 | | | |
| GO:0000324 | vacuole (sensu Fungi) | 2.797e-09 | E |
| GO:0000322 | storage vacuole | 2.797e-09 | E |
| GO:0000323 | lytic vacuole | 2.797e-09 | E |

  
  

|  |  |  |  |
| --- | --- | --- | --- |
| Class 2 | size: 3 gene products | | | |
| BNI5 |  CDC11 |  GIC2 | | | |
| GO:0005938 | cell cortex | 3.982e-07 | E |
| GO:0015629 | actin cytoskeleton | 3.982e-07 | E |
| GO:0005933 | bud | 7.963e-06 | E |
| GO:0030427 | site of polarized growth | 3.345e-05 | E |

  
  

|  |  |  |  |
| --- | --- | --- | --- |
| Class 3 | size: 5 gene products | | | |
| FYV7 |  KRR1 |  UTP30 |  ECM1 |  UTP4 | | | |
| GO:0005730 | nucleolus | 7.993e-10 | E |

  
  

|  |  |  |  |
| --- | --- | --- | --- |
| Class 4 | size: 39 gene products | | | |
| YER189W |  SRY1 |  ULA1 |  COS111 |  YHR029C |  MNT4 |  YNR068C |  YJL213W |  YIR041W |  AAD16 |  PRR2 |  UBX6 |  YHR138C |  YOR394W |  YPL088W |  YDR542W |  YKL121W |  HES1 |  YPL272C |  YER158C |  SDT1 |  RCR1 |  YKL224C |  YLR152C |  BSC5 |  YPL033C |  FUR1 |  YJL206C |  HSP33 |  RDS1 |  YGR110W |  YOR338W |  BOP2 |  NIT1 |  YPL282C |  YFR055W |  YGL117W |  YBR147W |  PDX3 | | | |
| GO:0008372 | cellular\_component unknown | 1.979e-46 | E |

  
  

|  |  |  |  |
| --- | --- | --- | --- |
| Class 5 | size: 39 gene products | | | |
| PIN3 |  YMR009W |  BCP1 |  NCE103 |  STR2 |  PPT1 |  AAP1prime |  YIL056W |  ADE3 |  RIB1 |  HSP26 |  FOL2 |  RTG3 |  ADH5 |  ZTA1 |  UPC2 |  AAH1 |  ARO4 |  BAT2 |  YOR289W |  MSN2 |  WTM1 |  YGR043C |  CUP2 |  BAS1 |  PIP2 |  SPT21 |  UGA3 |  FAP7 |  TEA1 |  STP2 |  MAC1 |  RIM101 |  TSA2 |  YHR127W |  HAC1 |  SIP4 |  PDR8 |  YJL113W | | | |
| GO:0005634 | nucleus | 5.888e-33 | E |
| GO:0043231 | intracellular membrane-bound organelle | 1.120e-14 | E |
| GO:0043227 | membrane-bound organelle | 1.120e-14 | E |
| GO:0043229 | intracellular organelle | 1.092e-13 | E |
| GO:0043226 | organelle | 1.092e-13 | E |
| GO:0005622 | intracellular | 2.317e-07 | E |
| GO:0005623 | cell | 0.000674 | E |
| GO:0005575 | cellular\_component | 1.000000 | E |

  
  

|  |  |  |  |
| --- | --- | --- | --- |
| Class 6 | size: 20 gene products | | | |
| ERG25 |  ERG28 |  ERG27 |  SUR4 |  CYB5 |  CHS1 |  ENB1 |  OPT1 |  RSB1 |  YNL115C |  CPS1 |  PHO12 |  YIL158W |  BOP1 |  NCR1 |  ICY1 |  AMS1 |  ATP15 |  STF1 |  GGC1 | | | |
| GO:0043231 | intracellular membrane-bound organelle | 2.033e-07 | E |
| GO:0043227 | membrane-bound organelle | 2.033e-07 | E |
| GO:0043229 | intracellular organelle | 5.870e-07 | E |
| GO:0043226 | organelle | 5.870e-07 | E |
| GO:0005737 | cytoplasm | 8.257e-07 | E |
| GO:0005622 | intracellular | 0.000590 | E |
| GO:0005623 | cell | 0.028054 | E |
| GO:0005575 | cellular\_component | 1.000000 | E |

  
  

|  |  |  |  |
| --- | --- | --- | --- |
| Class 7 | size: 12 gene products | | | |
| CHO1 |  BSD2 |  YET1 |  ERG3 |  ATF2 |  ERG5 |  SUR2 |  ERG2 |  ERG11 |  GPI11 |  ERG6 |  ERG1 | | | |
| GO:0005783 | endoplasmic reticulum | 5.843e-16 | E |

  
  

|  |  |  |  |
| --- | --- | --- | --- |
| Class 8 | size: 3 gene products | | | |
| PRM4 |  ERP3 |  YET2 | | | |
| GO:0016021 | integral to membrane | 8.760e-05 | E |

  
  

|  |  |  |  |
| --- | --- | --- | --- |
| Class 9 | size: 21 gene products | | | |
| PIN3 |  YMR009W |  BCP1 |  NCE103 |  STR2 |  PPT1 |  AAP1prime |  YIL056W |  ADE3 |  RIB1 |  HSP26 |  FOL2 |  RTG3 |  ADH5 |  ZTA1 |  UPC2 |  AAH1 |  ARO4 |  BAT2 |  YOR289W |  MSN2 | | | |
| GO:0005737 | cytoplasm | 3.911e-07 | E |

  
  

|  |  |  |  |
| --- | --- | --- | --- |
| Class 10 | size: 83 gene products | | | |
| SGE1 |  GAP1 |  ZRT1 |  MSB2 |  PRM4 |  ERP3 |  YET2 |  DAP1 |  ELO1 |  MCH1 |  AGP1 |  TPN1 |  SRO77 |  SSU1 |  ENA5 |  VHT1 |  YPS3 |  HXT2 |  BAP2 |  PHO89 |  ATR1 |  HBT1 |  AFR1 |  MSB3 |  PHO5 |  PLB1 |  YPS1 |  CWP1 |  YLR194C |  DAN1 |  TIR1 |  PST1 |  AGA1 |  TIR2 |  CRH1 |  PHO3 |  CPA2 |  LEU1 |  NTH1 |  YOR302W |  ARG4 |  ARG3 |  CPA1 |  ARG1 |  ADE17 |  PYC1 |  SSZ1 |  YOR385W |  BNA1 |  SER1 |  SER3 |  ARO10 |  DRE2 |  ARO8 |  SNO1 |  LYS2 |  YDR222W |  UGA2 |  PSA1 |  HUA1 |  DIA1 |  HOM3 |  GIR2 |  URA10 |  SRL3 |  ARO1 |  YJL016W |  BIO3 |  MET22 |  GYP7 |  TRP3 |  YEH1 |  PET10 |  HIS1 |  HIS5 |  MET16 |  HIS3 |  HIS4 |  YNL311C |  PCL5 |  TIF35 |  RIB5 |  SSB1 | | | |
| GO:0005623 | cell | 2.219e-08 | E |

  
  

|  |  |  |  |
| --- | --- | --- | --- |
| Class 11 | size: 8 gene products | | | |
| YNL115C |  CPS1 |  PHO12 |  YIL158W |  BOP1 |  NCR1 |  ICY1 |  AMS1 | | | |
| GO:0005773 | vacuole | 3.158e-15 | E |

  
  

|  |  |  |  |
| --- | --- | --- | --- |
| Class 12 | size: 11 gene products | | | |
| PHO5 |  PLB1 |  YPS1 |  CWP1 |  YLR194C |  DAN1 |  TIR1 |  PST1 |  AGA1 |  TIR2 |  CRH1 | | | |
| GO:0005618 | cell wall | 8.337e-17 | E |
| GO:0030312 | external encapsulating structure | 8.337e-17 | E |
| GO:0009277 | cell wall (sensu Fungi) | 8.337e-17 | E |

  
  

|  |  |  |  |
| --- | --- | --- | --- |
| Class 13 | size: 3 gene products | | | |
| TEF4 |  MRPL49 |  SED1 | | | |
| GO:0005840 | ribosome | 3.982e-07 | E |
| GO:0030529 | ribonucleoprotein complex | 7.963e-06 | E |
| GO:0043234 | protein complex | 0.000181 | E |
| GO:0043228 | non-membrane-bound organelle | 0.000454 | E |
| GO:0043232 | intracellular non-membrane-bound organelle | 0.000454 | E |
| GO:0043229 | intracellular organelle | 0.129604 | E |
| GO:0043226 | organelle | 0.129604 | E |
| GO:0005737 | cytoplasm | 0.135925 | E |
| GO:0005622 | intracellular | 0.343590 | E |
| GO:0005623 | cell | 0.597168 | E |
| GO:0005575 | cellular\_component | 1.000000 | E |

  
  

|  |  |  |  |
| --- | --- | --- | --- |
| Class 14 | size: 3 gene products | | | |
| ATP15 |  STF1 |  GGC1 | | | |
| GO:0005743 | mitochondrial inner membrane | 3.982e-07 | E |
| GO:0019866 | inner membrane | 3.982e-07 | E |
| GO:0005740 | mitochondrial membrane | 1.593e-06 | E |
| GO:0005739 | mitochondrion | 0.002383 | E |
| GO:0016020 | membrane | 0.004244 | E |

  
  

|  |  |  |  |
| --- | --- | --- | --- |
| Class 15 | size: 21 gene products | | | |
| SGE1 |  GAP1 |  ZRT1 |  MSB2 |  PRM4 |  ERP3 |  YET2 |  DAP1 |  ELO1 |  MCH1 |  AGP1 |  TPN1 |  SRO77 |  SSU1 |  ENA5 |  VHT1 |  YPS3 |  HXT2 |  BAP2 |  PHO89 |  ATR1 | | | |
| GO:0016020 | membrane | 1.711e-19 | E |

  
  

|  |  |  |  |
| --- | --- | --- | --- |
| Class 16 | size: 6 gene products | | | |
| FYV7 |  KRR1 |  UTP30 |  ECM1 |  UTP4 |  POL30 | | | |
| GO:0043228 | non-membrane-bound organelle | 1.275e-07 | E |
| GO:0043232 | intracellular non-membrane-bound organelle | 1.275e-07 | E |

  
  

|  |  |  |  |
| --- | --- | --- | --- |
| Class 17 | size: 37 gene products | | | |
| CPA2 |  LEU1 |  NTH1 |  YOR302W |  ARG4 |  ARG3 |  CPA1 |  ARG1 |  ADE17 |  PYC1 |  SSZ1 |  YOR385W |  BNA1 |  SER1 |  SER3 |  ARO10 |  DRE2 |  ARO8 |  SNO1 |  LYS2 |  YDR222W |  UGA2 |  PSA1 |  HUA1 |  DIA1 |  HOM3 |  GIR2 |  URA10 |  SRL3 |  ARO1 |  YJL016W |  BIO3 |  MET22 |  GYP7 |  TRP3 |  YEH1 |  PET10 | | | |
| GO:0005737 | cytoplasm | 1.226e-12 | E |

  
  

|  |  |  |  |
| --- | --- | --- | --- |
| Class 18 | size: 10 gene products | | | |
| CPA2 |  LEU1 |  NTH1 |  YOR302W |  ARG4 |  ARG3 |  CPA1 |  ARG1 |  ADE17 |  PYC1 | | | |
| GO:0005829 | cytosol | 1.417e-15 | E |

  
  

|  |  |  |  |
| --- | --- | --- | --- |
| Class 19 | size: 122 gene products | | | |
| YER189W |  SRY1 |  ULA1 |  COS111 |  YHR029C |  MNT4 |  YNR068C |  YJL213W |  YIR041W |  AAD16 |  PRR2 |  UBX6 |  YHR138C |  YOR394W |  YPL088W |  YDR542W |  YKL121W |  HES1 |  YPL272C |  YER158C |  SDT1 |  RCR1 |  YKL224C |  YLR152C |  BSC5 |  YPL033C |  FUR1 |  YJL206C |  HSP33 |  RDS1 |  YGR110W |  YOR338W |  BOP2 |  NIT1 |  YPL282C |  YFR055W |  YGL117W |  YBR147W |  PDX3 |  SGE1 |  GAP1 |  ZRT1 |  MSB2 |  PRM4 |  ERP3 |  YET2 |  DAP1 |  ELO1 |  MCH1 |  AGP1 |  TPN1 |  SRO77 |  SSU1 |  ENA5 |  VHT1 |  YPS3 |  HXT2 |  BAP2 |  PHO89 |  ATR1 |  HBT1 |  AFR1 |  MSB3 |  PHO5 |  PLB1 |  YPS1 |  CWP1 |  YLR194C |  DAN1 |  TIR1 |  PST1 |  AGA1 |  TIR2 |  CRH1 |  PHO3 |  CPA2 |  LEU1 |  NTH1 |  YOR302W |  ARG4 |  ARG3 |  CPA1 |  ARG1 |  ADE17 |  PYC1 |  SSZ1 |  YOR385W |  BNA1 |  SER1 |  SER3 |  ARO10 |  DRE2 |  ARO8 |  SNO1 |  LYS2 |  YDR222W |  UGA2 |  PSA1 |  HUA1 |  DIA1 |  HOM3 |  GIR2 |  URA10 |  SRL3 |  ARO1 |  YJL016W |  BIO3 |  MET22 |  GYP7 |  TRP3 |  YEH1 |  PET10 |  HIS1 |  HIS5 |  MET16 |  HIS3 |  HIS4 |  YNL311C |  PCL5 |  TIF35 |  RIB5 |  SSB1 | | | |
| GO:0005575 | cellular\_component | 1.000000 | E |

  
  

|  |  |  |  |
| --- | --- | --- | --- |
| Class 20 | size: 5 gene products | | | |
| BNI5 |  CDC11 |  GIC2 |  SPC42 |  SLI15 | | | |
| GO:0005856 | cytoskeleton | 1.332e-10 | E |
| GO:0043228 | non-membrane-bound organelle | 2.065e-06 | E |
| GO:0043232 | intracellular non-membrane-bound organelle | 2.065e-06 | E |
| GO:0043229 | intracellular organelle | 0.032533 | E |
| GO:0043226 | organelle | 0.032533 | E |
| GO:0005622 | intracellular | 0.167082 | E |
| GO:0005623 | cell | 0.421853 | E |
| GO:0005575 | cellular\_component | 1.000000 | E |

  
  

|  |  |  |  |
| --- | --- | --- | --- |
| Class 21 | size: 45 gene products | | | |
| CPA2 |  LEU1 |  NTH1 |  YOR302W |  ARG4 |  ARG3 |  CPA1 |  ARG1 |  ADE17 |  PYC1 |  SSZ1 |  YOR385W |  BNA1 |  SER1 |  SER3 |  ARO10 |  DRE2 |  ARO8 |  SNO1 |  LYS2 |  YDR222W |  UGA2 |  PSA1 |  HUA1 |  DIA1 |  HOM3 |  GIR2 |  URA10 |  SRL3 |  ARO1 |  YJL016W |  BIO3 |  MET22 |  GYP7 |  TRP3 |  YEH1 |  PET10 |  HIS1 |  HIS5 |  MET16 |  HIS3 |  HIS4 |  YNL311C |  PCL5 |  TIF35 | | | |
| GO:0005622 | intracellular | 1.617e-08 | E |

  
  

|  |  |  |  |
| --- | --- | --- | --- |
| Class 22 | size: 9 gene products | | | |
| FYV7 |  KRR1 |  UTP30 |  ECM1 |  UTP4 |  POL30 |  NVJ1 |  HAS1 |  SWE1 | | | |
| GO:0005634 | nucleus | 7.530e-07 | E |
| GO:0043231 | intracellular membrane-bound organelle | 0.001238 | E |
| GO:0043227 | membrane-bound organelle | 0.001238 | E |
| GO:0043229 | intracellular organelle | 0.001950 | E |
| GO:0043226 | organelle | 0.001950 | E |
| GO:0005622 | intracellular | 0.038663 | E |
| GO:0005623 | cell | 0.208538 | E |
| GO:0005575 | cellular\_component | 1.000000 | E |

  
  

|  |  |  |  |
| --- | --- | --- | --- |
| Class 23 | size: 4 gene products | | | |
| CTF13 |  MCD1 |  REC8 |  PDS5 | | | |
| GO:0000794 | condensed nuclear chromosome | 6.501e-09 | E |
| GO:0000793 | condensed chromosome | 6.501e-09 | E |

  
  

|  |  |  |  |
| --- | --- | --- | --- |
| Class 24 | size: 13 gene products | | | |
| YPT53 |  CHO1 |  BSD2 |  YET1 |  ERG3 |  ATF2 |  ERG5 |  SUR2 |  ERG2 |  ERG11 |  GPI11 |  ERG6 |  ERG1 | | | |
| GO:0043231 | intracellular membrane-bound organelle | 5.595e-05 | E |
| GO:0043227 | membrane-bound organelle | 5.595e-05 | E |
| GO:0043229 | intracellular organelle | 0.000109 | E |
| GO:0043226 | organelle | 0.000109 | E |
| GO:0005737 | cytoplasm | 0.000135 | E |
| GO:0005622 | intracellular | 0.008684 | E |
| GO:0005623 | cell | 0.101757 | E |
| GO:0005575 | cellular\_component | 1.000000 | E |

  
  

|  |  |  |  |
| --- | --- | --- | --- |
| Class 25 | size: 3 gene products | | | |
| NCR1 |  ICY1 |  AMS1 | | | |
| GO:0005774 | vacuolar membrane | 3.982e-07 | E |
| GO:0016020 | membrane | 0.004244 | E |

  
  

|  |  |  |  |
| --- | --- | --- | --- |
| Class 26 | size: 4 gene products | | | |
| SGE1 |  GAP1 |  ZRT1 |  MSB2 | | | |
| GO:0005887 | integral to plasma membrane | 3.250e-08 | E |
| GO:0016021 | integral to membrane | 3.218e-06 | E |
| GO:0005886 | plasma membrane | 2.520e-05 | E |

  
  

|  |  |  |  |
| --- | --- | --- | --- |
| Class 27 | size: 15 gene products | | | |
| ALT1 |  YNL208W |  IDP1 |  GCV2 |  ILV3 |  ILV2 |  LEU4 |  SCM4 |  FMP12 |  FMP23 |  YJR111C |  FMP43 |  YJL200C |  PDH1 |  IRS4 | | | |
| GO:0005739 | mitochondrion | 4.528e-15 | E |
| GO:0043231 | intracellular membrane-bound organelle | 1.154e-05 | E |
| GO:0043227 | membrane-bound organelle | 1.154e-05 | E |
| GO:0043229 | intracellular organelle | 2.510e-05 | E |
| GO:0043226 | organelle | 2.510e-05 | E |
| GO:0005737 | cytoplasm | 3.224e-05 | E |
| GO:0005622 | intracellular | 0.004068 | E |
| GO:0005623 | cell | 0.070725 | E |
| GO:0005575 | cellular\_component | 1.000000 | E |

  
  

|  |  |  |  |
| --- | --- | --- | --- |
| Class 28 | size: 5 gene products | | | |
| CTF13 |  MCD1 |  REC8 |  PDS5 |  HTA2 | | | |
| GO:0000228 | nuclear chromosome | 1.332e-10 | E |
| GO:0005694 | chromosome | 7.993e-10 | E |
| GO:0043228 | non-membrane-bound organelle | 2.065e-06 | E |
| GO:0043232 | intracellular non-membrane-bound organelle | 2.065e-06 | E |
| GO:0005634 | nucleus | 0.000463 | E |
| GO:0043231 | intracellular membrane-bound organelle | 0.025387 | E |
| GO:0043227 | membrane-bound organelle | 0.025387 | E |
| GO:0043229 | intracellular organelle | 0.032533 | E |
| GO:0043226 | organelle | 0.032533 | E |
| GO:0005622 | intracellular | 0.167082 | E |
| GO:0005623 | cell | 0.421853 | E |
| GO:0005575 | cellular\_component | 1.000000 | E |

  
  

|  |  |  |  |
| --- | --- | --- | --- |
| Class 29 | size: 5 gene products | | | |
| ERG25 |  ERG28 |  ERG27 |  SUR4 |  CYB5 | | | |
| GO:0042175 | nuclear envelope-endoplasmic reticulum network | 1.332e-10 | E |
| GO:0005789 | endoplasmic reticulum membrane | 1.332e-10 | E |
| GO:0012505 | endomembrane system | 2.797e-09 | E |
| GO:0005783 | endoplasmic reticulum | 1.549e-06 | E |

  
  

|  |  |  |  |
| --- | --- | --- | --- |
| Class 30 | size: 4 gene products | | | |
| PEX21 |  LYS1 |  AAT2 |  CIT2 | | | |
| GO:0005777 | peroxisome | 6.501e-09 | E |
| GO:0042579 | microbody | 6.501e-09 | E |
| GO:0043231 | intracellular membrane-bound organelle | 0.053401 | E |
| GO:0043227 | membrane-bound organelle | 0.053401 | E |
| GO:0043229 | intracellular organelle | 0.065067 | E |
| GO:0043226 | organelle | 0.065067 | E |
| GO:0005737 | cytoplasm | 0.069350 | E |
| GO:0005622 | intracellular | 0.239812 | E |
| GO:0005623 | cell | 0.502108 | E |
| GO:0005575 | cellular\_component | 1.000000 | E |

  
  

|  |  |  |  |
| --- | --- | --- | --- |
| Class 31 | size: 9 gene products | | | |
| ERG25 |  ERG28 |  ERG27 |  SUR4 |  CYB5 |  CHS1 |  ENB1 |  OPT1 |  RSB1 | | | |
| GO:0016020 | membrane | 4.149e-08 | E |

  
  

|  |  |  |  |
| --- | --- | --- | --- |
| Class 32 | size: 11 gene products | | | |
| AGP1 |  TPN1 |  SRO77 |  SSU1 |  ENA5 |  VHT1 |  YPS3 |  HXT2 |  BAP2 |  PHO89 |  ATR1 | | | |
| GO:0005886 | plasma membrane | 1.731e-14 | E |

  
  

|  |  |  |  |
| --- | --- | --- | --- |
| Class 33 | size: 3 gene products | | | |
| HBT1 |  AFR1 |  MSB3 | | | |
| GO:0030427 | site of polarized growth | 3.345e-05 | E |

  
  
