# Supplementary material for: Theme discovery from gene lists for identification and viewing of multiple functional groups
Source: BMC Bioinformatics. 2005 Jun 29;6:162. doi: 10.1186/1471-2105-6-162 (PMC1190153; doi:10.1186/1471-2105-6-162)
Supplement: Additional File 3 — H2O2 dataset analysed with SGD DAG View. Figure 8 The figure presents the three DAG tree figures obtained from SGD GO term finder with the H2O2 data. The reported classes are colour coded according the reported p-value. We have marked the classes that were reported by some cluster in GENERATOR results by adding the number of corresponding cluster. Note that many classes that were not reported by GENERATOR are usually close in the hierarchy to already reported classes. [file 1471-2105-6-162-S3.pdf]

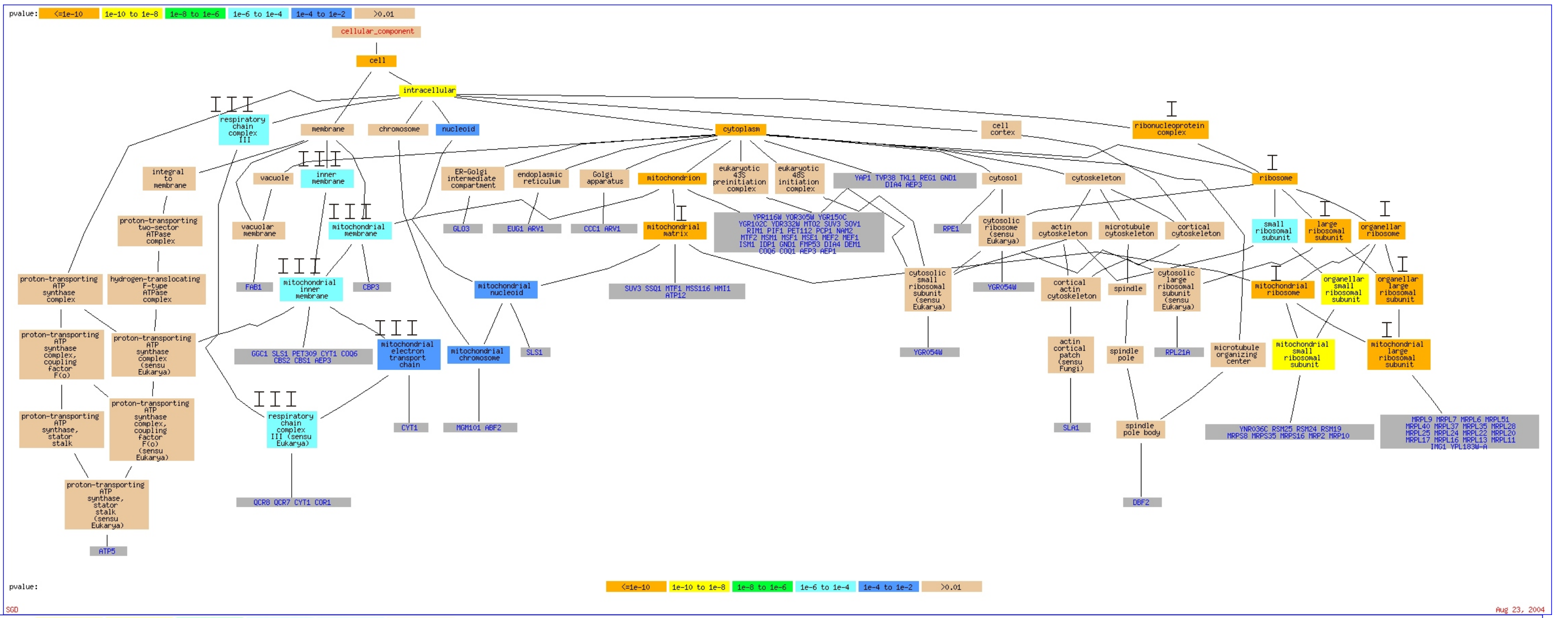

pvalue:
<=1e-10
1e-10 to 1e-8
1e-8 to 1e-6
1e-6 to 1e-4
1e-4 to 1e-2
>0.01
cellular_component
cell
intracellular
respiratory chain complex III
membrane
chromosome
nucleoid
cytoplasm
cell cortex
ribonucleoprotein complex
integral to membrane
vacuole
inner membrane
ER-Golgi intermediate compartment
endoplasmic reticulum
Golgi apparatus
mitochondrion
eukaryotic 43S preinitiation complex
eukaryotic 48S initiation complex
YAP1 TYP38 TKL1 REG1 GND1 DIA4 AEP3
cytosol
cytoskeleton
ribosome
proton-transporting two-sector ATPase complex
vacuolar membrane
mitochondrial membrane
GLO3
EUG1 ARV1
CCC1 ARV1
mitochondrial matrix
YPR116W YDR309W YGR150C YGR102C YDR332W HTD2 SUV3 SOV1 RIM1 PIF1 PET122 PCP1 NAM2 MTF2 MSM1 MSF1 MSE1 MEF2 MEF1 ISM1 IDP1 GND1 FMP53 DIA4 DEM1 COQ6 COQ1 AEP3 AEP1
RPE1
cytosolic ribosome (sensu Eukarya)
actin cytoskeleton
microtubule cytoskeleton
cortical cytoskeleton
small ribosomal subunit
large ribosomal subunit
organellar ribosome
proton-transporting ATP synthase complex
hydrogen-translocating F-type ATPase complex
FAB1
mitochondrial inner membrane
CBP3
mitochondrial nucleoid
SUV3 SSQ1 MTF1 MSS116 HMI1 ATP12
cytosolic small ribosomal subunit (sensu Eukarya)
YGR054W
cortical actin cytoskeleton
spindle
cytosolic large ribosomal subunit (sensu Eukarya)
mitochondrial ribosome
organellar small ribosomal subunit
organellar large ribosomal subunit
proton-transporting ATP synthase complex, coupling factor F(o)
proton-transporting ATP synthase complex (sensu Eukarya)
GGC1 SLS1 PET309 CYT1 COQ6 CBS2 CBS1 AEP3
mitochondrial electron transport chain
mitochondrial chromosome
SLS1
YGR054W
actin cortical patch (sensu Fungi)
spindle pole
RPL21A
microtubule organizing center
mitochondrial small ribosomal subunit
mitochondrial large ribosomal subunit
proton-transporting ATP synthase, stator stalk
proton-transporting ATP synthase complex, coupling factor F(o) (sensu Eukarya)
respiratory chain complex III (sensu Eukarya)
CYT1
MGM101 ABF2
SLA1
spindle pole body
YNR036C RSM25 RSM24 RSM19 MRPS8 MRPS35 MRPS16 MRP2 MRP10
MRPL9 MRPL7 MRPL6 MRPL51 MRPL40 MRPL37 MRPL35 MRPL28 MRPL25 MRPL24 MRPL22 MRPL20 MRPL17 MRPL16 MRPL13 MRPL11 IMG1 YPL183W-A
proton-transporting ATP synthase, stator stalk (sensu Eukarya)
QCR8 QCR7 CYT1 COR1
DBF2
ATP5
SGD
Aug 23, 2004

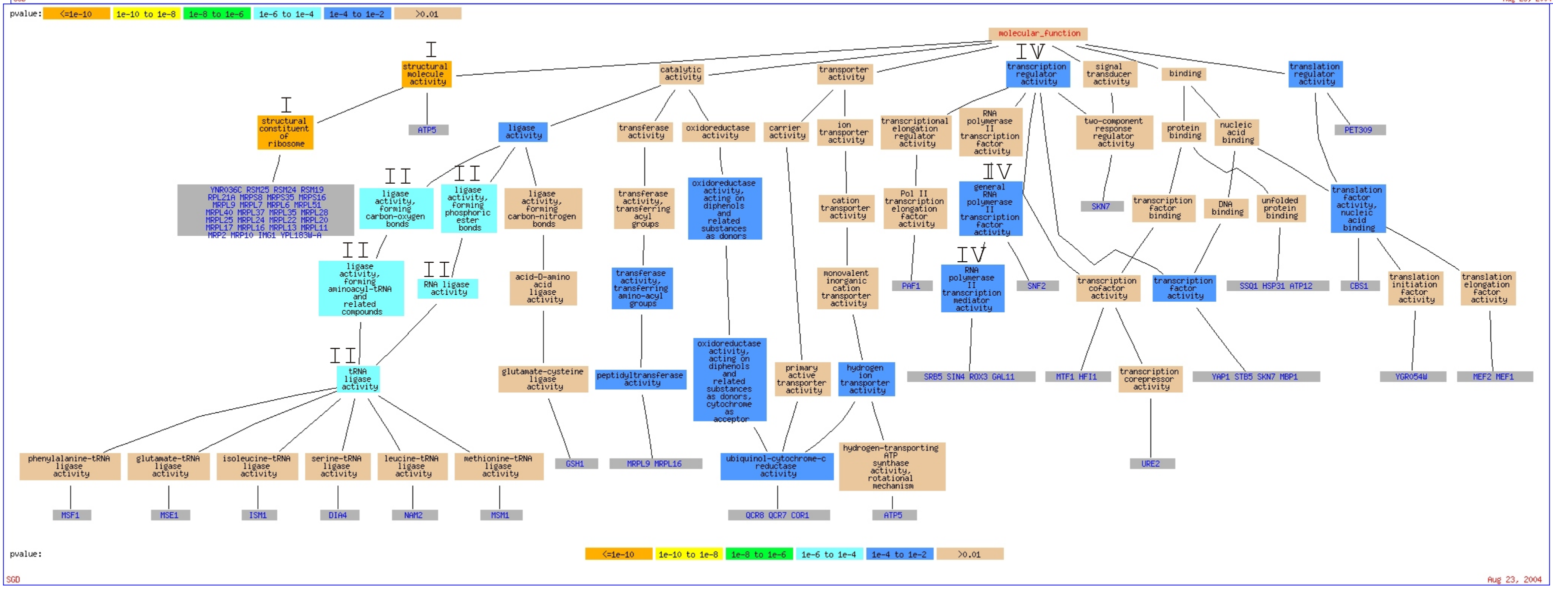

pvalue:
<=1e-10
1e-10 to 1e-8
1e-8 to 1e-6
1e-6 to 1e-4
1e-4 to 1e-2
>0.01
molecular_function
structural molecule activity
catalytic activity
transporter activity
transcription regulator activity
signal transducer activity
binding
translation regulator activity
structural constituent of ribosome
ATP5
ligase activity
transferase activity
oxidoreductase activity
carrier activity
ion transporter activity
transcriptional elongation regulator activity
RNA polymerase II transcription factor activity
two-component response regulator activity
protein binding
nucleic acid binding
PET309
YNR036C RSM25 RSM24 RSM19 RPL21A MRPS8 MRPS35 MRPS16 MRPL9 MRPL7 MRPL6 MRPL51 MRPL40 MRPL37 MRPL35 MRPL28 MRPL25 MRPL24 MRPL22 MRPL20 MRPL17 MRPL16 MRPL13 MRPL11 MRP2 MRP10 IMG1 YPL183W-A
ligase activity, forming carbon-oxygen bonds
ligase activity, forming phosphoric ester bonds
ligase activity, forming carbon-nitrogen bonds
transferase activity, transferring acyl groups
oxidoreductase activity, acting on diphenols and related substances as donors
cation transporter activity
Pol II transcription elongation factor activity
general RNA polymerase II transcription factor activity
SKN7
transcription factor binding
DNA binding
unfolded protein binding
translation factor activity, nucleic acid binding
ligase activity, forming aminoacyl-tRNA and related compounds
RNA ligase activity
acid-D-amino acid ligase activity
transferase activity, transferring amino-acyl groups
monovalent inorganic cation transporter activity
PAF1
RNA polymerase II transcription mediator activity
SNF2
transcription cofactor activity
transcription factor activity
SSQ1 HSP31 ATP12
CBS1
translation initiation factor activity
translation elongation factor activity
tRNA ligase activity
glutamate-cysteine ligase activity
peptidyltransferase activity
oxidoreductase activity, acting on diphenols and related substances as donors, cytochrome as acceptor
primary active transporter activity
hydrogen ion transporter activity
SRB5 SIN4 ROX3 GAL11
MTF1 HFI1
transcription corepressor activity
YAP1 STB5 SKN7 MBP1
YGR054W
MEF2 MEF1
phenylalanine-tRNA ligase activity
glutamate-tRNA ligase activity
isoleucine-tRNA ligase activity
serine-tRNA ligase activity
leucine-tRNA ligase activity
methionine-tRNA ligase activity
GSH1
MRPL9 MRPL16
ubiquinol-cytochrome-c reductase activity
hydrogen-transporting ATP synthase activity, rotational mechanism
URE2
MSF1
MSE1
ISM1
DIA4
NAM2
MSM1
QCR8 QCR7 COR1
ATP5
SGD
Aug 23, 2004

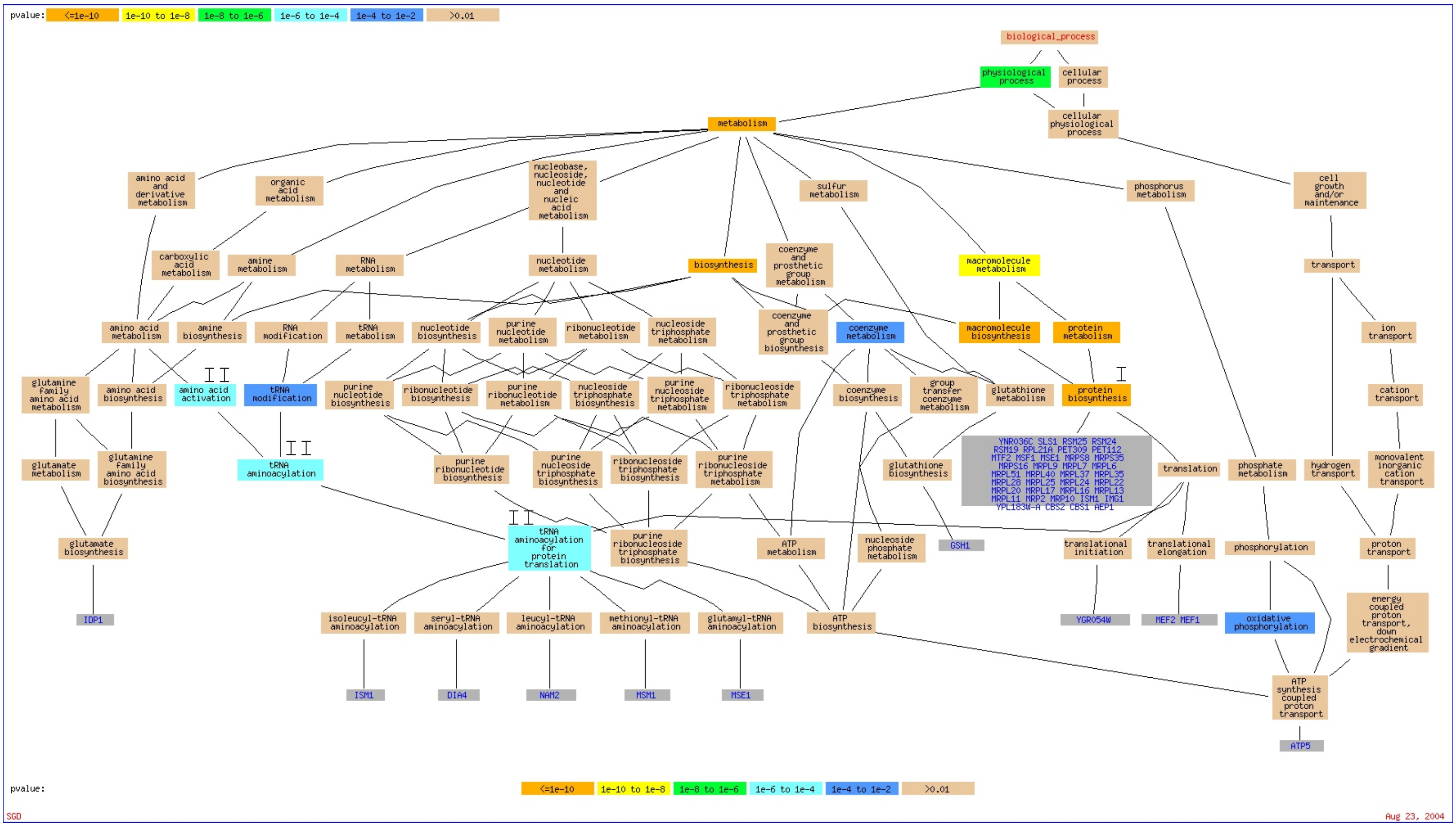

pvalue:
<=1e-10
1e-10 to 1e-8
1e-8 to 1e-6
1e-6 to 1e-4
1e-4 to 1e-2
>0.01
biological_process
physiological process
cellular process
metabolism
cellular physiological process
amino acid and derivative metabolism
organic acid metabolism
nucleobase, nucleoside, nucleotide and nucleic acid metabolism
sulfur metabolism
phosphorus metabolism
cell growth and/or maintenance
carboxylic acid metabolism
amine metabolism
RNA metabolism
nucleotide metabolism
biosynthesis
coenzyme and prosthetic group metabolism
macromolecule metabolism
transport
amino acid metabolism
amine biosynthesis
RNA modification
tRNA metabolism
nucleotide biosynthesis
purine nucleotide metabolism
ribonucleotide metabolism
nucleoside triphosphate metabolism
coenzyme and prosthetic group biosynthesis
coenzyme metabolism
macromolecule biosynthesis
protein metabolism
ion transport
glutamine family amino acid metabolism
amino acid biosynthesis
amino acid activation
tRNA modification
purine nucleotide biosynthesis
ribonucleotide biosynthesis
purine ribonucleotide metabolism
nucleoside triphosphate biosynthesis
purine nucleoside triphosphate metabolism
ribonucleoside triphosphate metabolism
coenzyme biosynthesis
group transfer coenzyme metabolism
glutathione metabolism
protein biosynthesis
cation transport
glutamate metabolism
glutamine family amino acid biosynthesis
tRNA aminoacylation
purine ribonucleotide biosynthesis
purine nucleoside triphosphate biosynthesis
ribonucleoside triphosphate biosynthesis
purine ribonucleoside triphosphate metabolism
glutathione biosynthesis
YNR036C SLS1 RSM25 RSM24 RSM19 RPL21A PET309 PET112 MTF2 MSF1 MSE1 MRPS8 MRPS35 MRPS16 MRPL9 MRPL7 MRPL6 MRPL51 MRPL40 MRPL37 MRPL35 MRPL28 MRPL25 MRPL24 MRPL22 MRPL20 MRPL17 MRPL16 MRPL13 MRPL11 MRP2 MRP10 ISM1 IMG1 YPL183W-A CBS2 CBS1 AEP1
translation
phosphate metabolism
hydrogen transport
monovalent inorganic cation transport
glutamate biosynthesis
tRNA aminoacylation for protein translation
purine ribonucleoside triphosphate biosynthesis
ATP metabolism
nucleoside phosphate metabolism
GSH1
translational initiation
translational elongation
phosphorylation
proton transport
energy coupled proton transport, down electrochemical gradient
IDP1
isoleucyl-tRNA aminoacylation
seryl-tRNA aminoacylation
leucyl-tRNA aminoacylation
methionyl-tRNA aminoacylation
glutamyl-tRNA aminoacylation
ATP biosynthesis
YGR054W
MEF2 MEF1
oxidative phosphorylation
ATP synthesis coupled proton transport
ISM1
DIA4
NAM2
MSM1
MSE1
ATP5
SGD
Aug 23, 2004
